# On the Schwartz Distribution Approach to Local Volatility for European Option in a Generalised Jump Model with Stochastic Interest Rate

**DOI:** 10.3390/e28101111

**Published:** 2026-10-12

**Authors:** Piotr Nowak, Dariusz Gatarek

**Affiliations:** Systems Research Institute, Polish Academy of Sciences, Newelska 6, 01-447 Warsaw, Poland; gatarek@ibspan.waw.pl

**Keywords:** Dupire formula, European option, Lévy-type stochastic integrals, local volatility, Schwartz distributions

## Abstract

The Dupire formula is commonly regarded as a convenient analytical tool for the valuation of financial derivatives. In this paper, we continue the analytical treatment developed in our previous papers, deriving the aforementioned formula for the European call option in the space of distributions. We consider asset prices driven by Lévy-type stochastic integrals in a framework that allows flexible jump structures and contains a class of stochastic short-rate models, including the Vasicek model. We prove existence and uniqueness for the underlying SDE and establish the Dupire formula in this general setting. More explicit versions of the Dupire formulas are obtained for two models: Merton’s model with the Vasicek short rate, and a model with time-dependent jump scaling.

## 1. Introduction

Local volatility models were brought into option pricing in the 1990s, when the shortcomings of the standard derivative-pricing formulas became apparent. In particular, the Black–Scholes model (see [1]) was found to be inadequate, as markets exhibited, inter alia, the volatility smile, which contradicts the model’s assumption of constant volatility (see, e.g., [2]). To address this phenomenon, Dupire [3] and Derman and Kani [4] initialised the class of local volatility models, introducing a deterministic function of two arguments: the spot price of the underlying asset *S* and time to the Black–Scholes model in order to describe the instantaneous volatility of *S*. The proposed form of instantaneous volatility preserves the favourable properties of the Black–Scholes approach.

Let Q be a risk-neutral probability measure and $E^{Q}$ be the expected value with respect to Q. In the classical version, the Dupire formula, which makes it possible to recover the volatility function from market prices of options (see, e.g., [5,6]), has the following form (see, e.g., [7])

(1) $$\begin{array}{c} \frac{\partial c}{\partial t}=\frac{1}{2}\sigma^{2}\left(t,k\right)k^{2}\frac{\partial^{2}c}{\partial k^{2}}-r_{t}k\frac{\partial c}{\partial k}, \end{array}$$

where *c* denotes the no-arbitrage price of the European call option with maturity *t* and strike price *k*, which at time zero is given by $c=c\left(t,k\right)=\exp\left(-\int_{0}^{t}r_{u}du\right)E^{Q}\left[{\left(S_{t}-k\right)}_{+}\right]$, *r* is a deterministic instantaneous risk-free interest rate, *S* is the price of a traded stock evolving according to the following equation with respect to Q

$$S_{t}=S_{0}+\int_{0}^{t}r_{u}S_{u}du+\int_{0}^{t}\sigma \left(u,S_{u}\right)S_{u}dW_{u},$$

σ is a deterministic local volatility function, and *W* is the standard Q-Brownian motion.

Equation (1) was extended by Andersen and Andreasen [8] to Markov jump-diffusions and by Carr et al. [9] for local Lévy models. An important development was achieved in [10], where Bentata and Cont derived the Dupire formula for European options in a semimartingale model of the underlying asset under a deterministic interest rate. The paper also contains references to earlier contributions concerning this formula. In many extensions of (1), the underlying-asset model is continuous (see, e.g., [11,12,13]); however, Benhamou et al. [14] considered a discontinuous asset-price process. Dupire’s formula for asset exchange options (see, e.g., [15] and also [16] and references therein) was derived by Gatarek and Jabłecki [17] and Nowak and Gatarek [18,19] in cases where the underlying assets are described either by Itô processes or by Lévy-type stochastic integrals. A similar approach was applied to the case of a European option and to both Merton models, the 1973 model for the instantaneous interest rate and the 1976 model for the underlying asset, by Nowak and Gatarek [20]. Other important contributions include the work of Hambly et al. [21] for barrier options and the paper by Hainaut and Leonenko [22], which considers the fractional version of the Dupire formula and a model based on invertible Lévy subordinators.

In many instances, the Dupire formula was derived in a heuristic manner, without verifying the required assumptions. This led to questions about the correctness and applicability of the resulting formula in numerous concrete situations. Among practitioners, it created a need for a mathematically rigorous justification of the formula. Our approach, developed in this and our earlier papers and carried out in the space of distributions, addresses this need. An equation for this formula in that space was also derived in [10].

In this paper, we continue our analytical treatment by providing a mathematically rigorous proof of the Dupire formula for European call options in the space of distributions, based on a generalised Dupire equation for asset exchange options, derived in [19] through a new stochastic approach. We assume that asset prices are described by Lévy-type stochastic integrals. In [20], we obtained the Dupire equation for an underlying financial instrument with dynamics modelled by the Merton equation (see, e.g., [8]) and the Merton interest rate model of 1973 (see, e.g., [23]). A simplified version of the proof of the Dupire formula, without using distributions, was presented by Benhamou et al. [14]. In their treatment, the stochastic short rate was not specified; instead, they worked with Merton’s model for the underlying asset. In contrast to [14,20], which focus on a fixed short-rate specification or the Merton jump structure for the asset price, the main result of this paper concerns a more general setting, as it covers a class of stochastic short-rate models including the Vasicek model and, at the same time, enables a jump structure for the asset price richer than in Merton’s exponential jump-diffusion. An essential contribution of this paper is a proof of existence and uniqueness for the SDE that describes the general dynamics of the underlying-asset price with an exogenous stochastic short rate under the risk-neutral measure. The solution lies in a class of processes that makes it possible to establish the Dupire formula in this more general setting. There is earlier literature on SDEs with jumps and random non-Lipschitz coefficients (for example, [24]). The equation considered in the mentioned paper is very general, but the assumptions include almost sure continuity of the coefficients. The formulation studied in the present paper, with the drift $b\left(t,x,\omega \right)=xr_{t-}\left(\omega \right)$, the tail condition on the adapted càdlàg short rate *r*, the finite-activity jump structure, and the moment class needed for the Dupire result, is of a different nature.

Additionally, we apply the general Dupire formula, established in Theorem 3, to two modelling settings. The first combines the Merton asset model with the Vasicek interest rate, while the second involves an interest rate satisfying a tail condition together with an asset model that incorporates time-dependent jump scaling. In both settings, we derive the Dupire formulas explicitly. We further show that without the tail condition on the interest rate, the main result of Theorem 3 cannot be used: the solution of the corresponding SDE fails to satisfy the regularity assumptions required for the general Dupire equation. In our opinion, the broad area of derivative pricing under discontinuous models for the underlying-asset dynamics, within which the Dupire formula naturally falls, merits investigation, since such models reflect market phenomena associated with asset-price jumps in a more realistic way than continuous stochastic models. In this paper, we verify all the modelling assumptions and apply advanced methods of mathematical finance and stochastic analysis, including the theory of SDEs.

The Dupire equations in this paper, similarly to [18,19,20], are obtained in the space of distributions. It follows from the fact that there are difficulties in the application of classical methods of analysis due to the non-differentiability of the option payoff function. The use of the distributional approach, however, required high-level stochastic-analysis techniques, especially in the proofs of the most general versions of the Dupire formulas (see [18,19]).

Our results are purely theoretical. The formulas we obtain are derived rigorously from a mathematical standpoint and can be applied directly to the valuation of European options under the assumption of no arbitrage. However, since they are established in the space of distributions, their numerical implementation requires further investigation. In [20], we showed that the minimal entropy martingale measure can be used as a risk-neutral probability measure. For Lévy-type stochastic integrals, the notion of a minimal entropy martingale measure provides a natural reference framework, and its role in relation to the present setting is discussed in Section 4.3.

Our previous papers [19,20], like the present one, were devoted to the derivation of the Dupire formula for options in the space of distributions. In [19], we derive and prove a generalised version of the mentioned formula for asset exchange options. We apply Lévy-type stochastic integrals to model the prices of the underlying assets, thus capturing jumps in their dynamics. Our approach is general and does not assume any specific SDEs governing price dynamics, and in particular, it allows the derivation of the formula for European options. In that paper, as an example, we apply the main theorem to derive the Dupire formula for a European option in the Merton (1976) model with a constant interest rate. In [20], we use the theory developed in [19] (citing the preprint of that article) to derive the Dupire equation for a European option, where the underlying financial instrument follows the model proposed by Merton in 1976 and the short-rate dynamics are given by the Merton interest-rate model of 1973. In that paper, we do not study the existence and uniqueness of the SDE for the undiscounted price of the underlying asset; instead, we consider the discounted process and the specific properties of both models. In this paper, we derive a general version of the Dupire formula for a European option within the undiscounted asset-price model (13), using the theory developed in [19]. The jump structure in (13) is flexible and extends the classical Merton model, while the moment properties of the solution follow from Assumption 2 together with a tail condition on the stochastic short rate, satisfied, for example, by the Vasicek model. We also show that the discounted price process is a martingale, ensuring the absence of arbitrage. As illustrations, we present the corresponding forms of the Dupire equation for the Merton jump-diffusion with Vasicek interest-rate dynamics and for an asset model with time-dependent jump scaling.

The structure of this paper is as follows. Section 2 provides the preliminaries together with the theoretical results required in the following sections, with particular emphasis on distributions, stochastic integrals with respect to a compensated random measure, and the generalised version of the Dupire equation for asset exchange options. Section 3 establishes existence and uniqueness for the stochastic differential equation describing the general version of the underlying-asset model used for the option under consideration, and identifies the regularity property of its solution, together with the conditions imposed on the interest rate and the behaviour of the discount factor within the model. Section 4 presents the main results of this paper, including the derivation and proof of the general version of the Dupire formula, as well as its two particular versions: for the Merton asset model with the Vasicek interest rate and for an interest rate satisfying a tail condition together with an asset model featuring time-dependent jump scaling. Finally, Section 5 summarises the findings.

## 2. Preliminaries

This section introduces the notation, definitions, and foundational results needed in the sequel. We collect the basic facts concerning random measures, stochastic integrals with respect to random and compensated random measures, and Lévy-type stochastic integrals. In addition, we present the main theorem obtained in [19], concerning the Dupire formula in the space of distributions, which will be used in the main part of this paper. We also introduce the basic notation used throughout the paper, including the notation arising from the theory of distributions.

### 2.1. Notation and Basic Concepts

We use the following notation: R for the set of real numbers, $R_{+}$ for the set of non-negative real numbers, Z for the set of integers, N for the set of positive integers, T for the interval [0,T] with 0<T∈R, and [k:l] for the set {k,k+1,…,l}, with k,l∈Z, k≤l.

We write B(E) for the Borel σ-algebra on a measurable space *E*, $1_{A}$ for the indicator function of a set *A*, l(dt)=dt for the Lebesgue measure on T, and L(T) for the Lebesgue σ-algebra on T.

Given closed interval I=[a,b]⊆T, a function f:[a,b]→R is called càdlàg if it is right-continuous on [a,b) and has left limits on (a,b]. Throughout, we adopt the convention $Z_{0-}=Z_{0}$ for every càdlàg, adapted stochastic process *Z*.

We also use the notation $L_{loc}^{1}\left(\Upsilon \right)$ for the space of locally integrable functions on a non-empty open set $\Upsilon \subset R^{2}$, D(Υ) for the space of $C^{\infty }$-functions of compact support in Υ, which is the space of test functions of general (often called Schwartz) distributions, and $D^{\prime }\left(\Upsilon \right)$ for the space of general distributions, being the space of all continuous linear functionals on D(Υ). For background concerning the topologies on D(Υ) and $D^{\prime }\left(\Upsilon \right)$, see [25].

Let $\emptyset \neq \Upsilon_{1}\subset \Upsilon_{2}\subset R^{2}$ be open and $\iota_{\Upsilon_{2},\Upsilon_{1}}:D\left(\Upsilon_{1}\right)\to D\left(\Upsilon_{2}\right)$ be a continuous linear function which assigns to an element of $D(\Upsilon_{1})$ its continuation by 0 to $\Upsilon_{2}$. Then, we introduce the function $\iota_{\Upsilon_{1},\Upsilon_{2}}:D^{\prime }\left(\Upsilon_{2}\right)\to D^{\prime }\left(\Upsilon_{1}\right)$ by the formula

$$\iota_{\Upsilon_{1},\Upsilon_{2}}\left(\varphi \right)=\varphi \circ \iota_{\Upsilon_{2},\Upsilon_{1}},\;\;\varphi \in D^{\prime }\left(\Upsilon_{2}\right).$$

In addition, we introduce the notations for the following spaces and functions.

We write

$$\begin{array}{cc} \; & D=\iota_{R^{2},\left(0,T\right)\times R}\left(D\left(\left(0,T\right)\times R\right)\right),\;D^{\prime }=\iota_{\left(0,T\right)\times R,R^{2}}\left(D^{\prime }\left(R^{2}\right)\right), \end{array}$$

and

$$\begin{array}{cc} \; & g_{a}:R\to R,\;k\mapsto g_{a}\left(k\right)=1_{\left(0,\infty \right)}\left(a-k\right),\\ \; & j\left(s,h\right):R\to R,\;k\mapsto j\left(s,h\right)\left(k\right)={\left(s+h-k\right)}_{+}-{\left(s-k\right)}_{+}-hg_{s}\left(k\right) \end{array}$$

for a,s,h∈R.

We now recall the definition of the notion of a finite transition kernel.

**Definition** **1.** *Let $\left(\Omega_{1},A_{1}\right)$ and $\left(\Omega_{2},A_{2}\right)$ be measurable spaces. A function $\kappa :\Omega_{1}\times A_{2}\to \left[0,\infty \right)$ is called a finite transition kernel (from $\left(\Omega_{1},A_{1}\right)$ to $\left(\Omega_{2},A_{2}\right)$) if it obeys the following conditions:*

*(i)*  *$\omega_{1}\mapsto \kappa \left(\omega_{1},A_{2}\right)$ is $A_{1}$-measurable for each $A_{2}\in A_{2}$;*
*(ii)*  *$A_{2}\mapsto \kappa \left(\omega_{1},A_{2}\right)$ is a finite measure on $\left(\Omega_{2},A_{2}\right)$ for each $\omega_{1}\in \Omega_{1}$.*

### 2.2. The Class of Lévy-Type Stochastic Integrals

Real stochastic processes are discussed, for example, in [26].

Throughout this subsection, we use a filtered probability space $\left(\Omega ,F,{\left(F_{t}\right)}_{t\in T},Q\right)$ on which the usual conditions are imposed. We write $E^{Q}$ (or simply E) to denote the expected value with respect to Q.

In this paper, Lévy-type stochastic integrals are used to model the underlying assets under consideration. For this reason, we recall selected elements of the theory related to these processes.

The notion of a Lévy process and the associated measures are introduced using the definitions from [27].

Let $X={\left(X_{t}\right)}_{t\in T}$ taking values in R be a Lévy process, meaning a càdlàg, stochastically continuous, $F_{t}$-adapted process with stationary and independent increments and satisfying $X_{0}=0$ a.s. We denote by N(dtdx) the measure on B(T×(R∖{0})), referred to as *the jump measure of* X (or *the Poisson random measure associated to* X), expressed as

$$\begin{array}{cc} N(\left(t_{1},t_{2}\right]\times A)= & \;\mathrm{the}\;\mathrm{number}\;\mathrm{of}\;\mathrm{jumps}\;\mathrm{of}\;X\;\mathrm{in}\;\left(t_{1},t_{2}\right]\;\mathrm{for}\;\mathrm{which}\;\Delta X_{s}\in A,\;s\in \left(t_{1},t_{2}\right], \end{array}$$

where $0\leq t_{1}<t_{2}\leq T$ and A∈B(R∖{0}). In turn, the measure $\bar{\nu }$ on B(R∖{0}) given by

$$\begin{array}{c} \bar{\nu }\left(A\right):=E^{Q}N\left(\left(0,1\right]\times A\right), \end{array}$$

where A∈B(R∖{0}), is referred to as *the Lévy measure of* X. Next, *the compensated jump measure of* X (also called *the compensated Poisson random measure associated to *X) is given by

$$\begin{array}{c} q\left(dt\;dx\right)=N\left(dt\;dx\right)-\bar{\nu }\left(dx\right)dt. \end{array}$$

Throughout this paper, we assume that measure $\bar{\nu }$ is finite.

We present several definitions and facts from the theory of stochastic integration of real-valued functions with respect to a compensated Poisson random measure, as discussed in [28], and adapt them to the finite time horizon T.

We denote by $\hat{P}$ the σ-algebra on T×Ω×(R∖{0}) generated by the maps g:T×Ω×(R∖{0})→R such that:

(a) for each t∈T, the map Ω×(R∖{0})∋(ω,z)↦g(t,ω,z)∈R is $B\left(R\setminus \left\{0\right\}\right)⊗F_{t}/B\left(R\right)$-measurable;
(b) for each (ω,z)∈Ω×(R∖{0}), the map T∋t↦g(t,ω,z)∈R is left-continuous.

A process g:T×Ω×(R∖{0})→R is called predictable provided it is $\hat{P}/B\left(R\right)$-measurable (see [28] (Def. 3.2.1)).

We use the symbol $M_{loc}^{2}\left(\hat{P},\bar{\nu },R\right)$ to denote the space of predictable processes V:T×Ω×(R∖{0})→R such that for each T∈(0,T]

$$E^{Q}\int_{0}^{T}\int_{R\setminus \{0\}}{\left|V\left(t,\omega ,z\right)\right|}^{2}\bar{\nu }\left(dz\right)dt<\infty .$$

If $V\in M_{loc}^{2}\left(\hat{P},\bar{\nu },R\right)$, then by [28] (Thm. 3.2.27), for each T∈(0,T], the stochastic integral of *V* with respect to *q* is well defined, and we use the notation $\int_{0}^{T}\int_{R\setminus \{0\}}V\left(t,\omega ,z\right)q\left(dt\;dz\right)$, $\int_{0}^{T}\int_{R\setminus \{0\}}V\left(t,z\right)q\left(dt\;dz\right)$, or, in shortened form, $\int_{0}^{T}\int_{R}V\left(t,z\right)q\left(dt\;dz\right)$. Moreover, this integral defines a random variable, which is square-integrable.

For our purposes, we take T=T. If $V\in M_{loc}^{2}\left(\hat{P},\bar{\nu },R\right)$, 0≤a≤b≤T, and B∈B(R∖{0}), then $1_{\left(a,b\right]}1_{B}V\in M_{loc}^{2}\left(\hat{P},\bar{\nu },R\right)$ and we write

$$\int_{a}^{b}\int_{B}V\left(t,z\right)q\left(dt\;dz\right):=\int_{0}^{T}\int_{R\setminus \{0\}}1_{\left(a,b\right]}1_{B}V\left(t,z\right)q\left(dt\;dz\right).$$

From [28] (Thm. 3.3.2), it follows that for each t∈T, $V_{1},V_{2}\in M_{loc}^{2}\left(\hat{P},\bar{\nu },R\right)$, and α,β∈R,

(i) $\begin{array}{l} \int_{0}^{t}\int_{R\setminus \{0\}}\left(\alpha V_{1}\left(t,z\right)+\beta V_{2}\left(t,z\right)\right)q\left(dt\;dz\right)\\ =\alpha \int_{0}^{t}\int_{R\setminus \{0\}}V_{1}\left(t,z\right)q\left(dt\;dz\right)+\beta \int_{0}^{t}\int_{R\setminus \{0\}}V_{2}\left(t,z\right)q\left(dt\;dz\right); \end{array}$
(ii) (2) $$E^{Q}\int_{0}^{t}\int_{R\setminus \{0\}}V_{1}\left(t,z\right)q\left(dt\;dz\right)=0;$$
(iii) $E^{Q}|\int_{0}^{t}\int_{R\setminus \{0\}}V_{1}{\left(t,z\right)q\left(dt\;dz\right)|}^{2}=E^{Q}\int_{0}^{t}\int_{R\setminus \{0\}}{\left|V_{1}\left(t,z\right)\right|}^{2}\bar{\nu }\left(dz\right)dt$;
(iv) ${\left(\int_{0}^{t}\int_{R\setminus \{0\}}V_{1}\left(t,z\right)q\left(dt\;dz\right)\right)}_{t\in T}$ admits a càdlàg modification that is a square-integrable martingale.

In what follows, the integrals of $V\in M_{loc}^{2}\left(\hat{P},\bar{\nu },R\right)$ with respect to *q* will be regarded as càdlàg processes.

Additionally, if one assumes that $\bar{\nu }\left(D\right)<\infty$ for D∈B(R∖{0}) (which always holds in the following part of this paper), by [28] (Proposition 3.4.5), one can then consider the integral of $V\in M_{loc}^{2}\left(\hat{P},\bar{\nu },R\right)$ with respect to N and for each t∈T,

(3) $$\begin{array}{c} \begin{array}{cc} \int_{0}^{t}\int_{D} & V\left(s,z\right)N\left(ds\;dz\right):=\sum_{s\in \left(0,t\right]:|\Delta X_{s}|>0}V\left(s,\Delta X_{s}\right)1_{D}\left(\Delta X_{s}\right)\\ \; & =\int_{0}^{t}\int_{D}V\left(s,z\right)q\left(ds\;dz\right)+\int_{0}^{t}\int_{D}V\left(s,z\right)\bar{\nu }\left(dz\right)ds. \end{array} \end{array}$$

We call a real semimartingale $X={\left(X\right)}_{t\in T}$ a Lévy-type stochastic integral (for its more general form we refer the reader to [29]) if it is given by the formula

(4) $$\begin{array}{cc} dX_{t} & =G_{s}ds+\sum_{j=1}^{m}F_{s}^{j}dW_{s}^{j}+\int_{|x|<1}H\left(t,x\right)q\left(dt\;dx\right)+\int_{|x|\geq 1}K\left(t,x\right)N\left(dt\;dx\right), \end{array}$$

where $G,F^{j}\in M\left(T\right)$, 1≤j≤m, $H1_{\left\{|x|<1\right\}}\left(x\right)$, $K1_{\left\{|x|\geq 1\right\}}\left(x\right)\in M_{loc}^{2}\left(\hat{P},\bar{\nu },R\right)$, $B=(W^{1},W^{2},\ldots ,W^{m})$ is the *m*-dimensional Brownian motion independent of N, and M(T) is the space of predictable processes U:T×Ω→R such that $E^{Q}\int_{0}^{T}{\left|U\left(t\right)\right|}^{2}dt<\infty$.

### 2.3. A Generalised Form of the Dupire Formula for Asset Exchange Options

We now turn to the assumptions and the structure of the generalised Dupire formula, established for asset exchange options in the Lévy-type stochastic integrals setting in [19].

All stochastic processes and random variables in this subsection are defined on the same filtered probability space as in the preceding subsection. We also assume that the probability measure Q is risk-neutral.

Let $R_{t}=\exp\left(-\int_{0}^{t}r_{u}du\right),\;t\in T$ be a stochastic process modelling a numeraire, which represents the time value of money, with $r={\left(r_{t}\right)}_{t\in T}$ denoting the short rate, that is, the instantaneous risk-free interest rate.

We additionally introduce stochastic processes $N={\left(N_{t}\right)}_{t\in T}$, $Y={\left(Y_{t}\right)}_{t\in T}$, $\tilde{N}={\left({\tilde{N}}_{t}\right)}_{t\in T}$, $\tilde{Y}={\left({\tilde{Y}}_{t}\right)}_{t\in T}$, and $S={\left(S_{t}\right)}_{t\in T}$, employed to model asset prices, with $N_{t}>0$ a.s., t∈T, ${\tilde{N}}_{t}=N_{t}R_{t},\;{\tilde{Y}}_{t}=Y_{t}R_{t},\;S_{t}=Y_{t}/N_{t}={\tilde{Y}}_{t}/{\tilde{N}}_{t},\;t\in T.$ Hence, $\tilde{N}$ and $\tilde{Y}$ describe the discounted values of *N* and *Y*, respectively, and *S* represents “the exchange rate” between them.

Furthermore, in [19], it is assumed that $\tilde{N}={\left({\tilde{N}}_{t}\right)}_{t\in T}$ and $S={\left(S_{t}\right)}_{t\in T}$ are described by Lévy-type stochastic integrals of the following form when considered under Q

(5) $$\begin{array}{cc} \; & d{\tilde{N}}_{t}=\alpha_{t}dW_{t}^{\left(1\right)}+\beta_{t}dt+\int_{|y|<1}{\hat{h}}_{1}\left(t,y\right)q\left(dt\;dy\right)+\int_{|y|\geq 1}{\hat{k}}_{1}\left(t,y\right)N\left(dt\;dy\right),\;{\tilde{N}}_{0}>0, \end{array}$$

(6) $$\begin{array}{cc} \; & dS_{t}=\sigma_{t}dW_{t}^{\left(2\right)}+\gamma_{t}dt+\int_{|y|<1}{\hat{h}}_{2}\left(t,y\right)q\left(dt\;dy\right)+\int_{|y|\geq 1}{\hat{k}}_{2}\left(t,y\right)N\left(dt\;dy\right),\;S_{0}\in R_{+}, \end{array}$$

where $W^{\left(i\right)}={\left(W_{t}^{\left(i\right)}\right)}_{t\in T},\;i\in \left\{1,2\right\}$, are the standard Brownian motions independent of N, ${\left[W_{t}^{\left(1\right)},W_{t}^{\left(2\right)}\right]}_{t}=\rho t,\;t\in T,\left|\rho \right|\leq 1$, β,γ,α,σ∈M(T), ${\hat{h}}_{1}1_{|y|<1}\left(y\right),{\hat{h}}_{2}1_{|y|<1}\left(y\right),{\hat{k}}_{1}1_{|y|\geq 1}\left(y\right)$, ${\hat{k}}_{2}1_{|y|\geq 1}\left(y\right)\in M_{loc}^{2}\left(\hat{P},\bar{\nu },R\right)$, X, N, *q*, $\bar{\nu }$ are introduced in Section 2.2, and the inequality below holds

(7) $$\begin{array}{c} \begin{array}{cc} E^{Q}\int_{0}^{T} & \left[\alpha_{t}^{2}+\beta_{t}^{2}+\sigma_{t}^{2}+\gamma_{t}^{2}+\int_{|y|<1}\left({\hat{h}}_{1}^{4}\left(t,y\right)+{\hat{h}}_{2}^{4}\left(t,y\right)\right)\bar{\nu }\left(dy\right)\right.\\ \; & \left.+\int_{|y|\geq 1}\left({\hat{k}}_{1}^{4}\left(t,y\right)+{\hat{k}}_{2}^{4}\left(t,y\right)\right)\bar{\nu }\left(dy\right)+S_{t-}^{2}\left(1+\alpha_{t}^{2}+\int_{|y|<1}{\hat{h}}_{1}^{2}\left(t,y\right)\bar{\nu }\left(dy\right)\right.\right.\\ \; & \left.\left.+\int_{|y|\geq 1}{\hat{k}}_{1}^{2}\left(t,y\right)\bar{\nu }\left(dy\right)\right)+{\tilde{N}}_{t-}^{2}\left(1+S_{t-}^{2}+\sigma_{t}^{2}+\int_{|y|<1}{\hat{h}}_{2}^{2}\left(t,y\right)\bar{\nu }\left(dy\right)\right.\right.\\ \; & \left.\left.+\int_{|y|\geq 1}{\hat{k}}_{2}^{2}\left(t,y\right)\bar{\nu }\left(dy\right)\right)\right]dt<\infty . \end{array} \end{array}$$

The differential of the process

(8) $${\tilde{Y}}_{t}={\tilde{N}}_{t}S_{t},\;t\in T,$$

takes the form

$$d{\tilde{Y}}_{t}=\phi_{t}^{\left(1\right)}dW_{t}^{\left(1\right)}+\phi_{t}^{\left(2\right)}dW_{t}^{\left(2\right)}+\lambda_{t}dt+\int_{|y|<1}\xi^{\left(1\right)}\left(t,y\right)q\left(dt\;dy\right)+\int_{|y|\geq 1}\xi^{\left(2\right)}\left(t,y\right)N\left(dt\;dy\right),$$

with

$$\begin{array}{cc} \; & \phi_{t}^{\left(1\right)}=S_{t-}\alpha_{t},\;\phi_{t}^{\left(2\right)}={\tilde{N}}_{t-}\sigma_{t},\;\lambda_{t}={\tilde{N}}_{t-}\gamma_{t}+S_{t-}\beta_{t}+\rho \alpha_{t}\sigma_{t}+\int_{|y|<1}{\hat{h}}_{1}\left(t,y\right){\hat{h}}_{2}\left(t,y\right)\bar{\nu }\left(dy\right),\\ \; & \xi^{\left(1\right)}\left(t,y\right)={\tilde{N}}_{t-}{\hat{h}}_{2}\left(t,y\right)+S_{t-}{\hat{h}}_{1}\left(t,y\right)+{\hat{h}}_{1}\left(t,y\right){\hat{h}}_{2}\left(t,y\right),\\ \; & \xi^{\left(2\right)}\left(t,y\right)={\tilde{N}}_{t-}{\hat{k}}_{2}\left(t,y\right)+S_{t-}{\hat{k}}_{1}\left(t,y\right)+{\hat{k}}_{1}\left(t,y\right){\hat{k}}_{2}\left(t,y\right). \end{array}$$

We introduce the following auxiliary functions.

$$\begin{array}{cc} \; & {\tilde{\hat{h}}}_{1}\left(t,y\right)={\hat{h}}_{1}\left(t,y\right)1_{|y|<1}\left(y\right),{\tilde{\hat{h}}}_{2}\left(t,y\right)={\hat{h}}_{2}\left(t,y\right)1_{|y|<1}\left(y\right),\;{\tilde{\hat{k}}}_{1}\left(t,y\right)={\hat{k}}_{1}\left(t,y\right)1_{|y|\geq 1}\left(y\right),\\ \; & {\tilde{\hat{k}}}_{2}\left(t,y\right)={\hat{k}}_{2}\left(t,y\right)1_{|y|\geq 1}\left(y\right),\;{\tilde{\xi }}^{\left(2\right)}\left(t,y\right)=\xi^{\left(2\right)}\left(t,y\right)1_{|y|\geq 1}\left(y\right). \end{array}$$

The integrals appearing in the expression for $\tilde{Y}$ were shown in [19] to be well defined.

Derivative-pricing theory implies that under the risk-neutral measure Q, the no-arbitrage time-zero value of a contingent claim Ξ is given by the $E^{Q}$-expectation of its pay-off discounted by the process $R_{t}$. In the considered case of the asset exchange option, the claim Ξ is interpreted as the European option to exchange the asset with price dynamics given by *Y* for *k* units of the asset described by the price process *N*. Thus, the no-arbitrage time-zero price c(t,k) of the claim Ξ maturing at t∈T is given by

(9) $$\begin{array}{c} \begin{array}{cc} c\left(t,k\right) & =E^{Q}\left[R_{t}{\left(Y_{t}-kN_{t}\right)}_{+}\right]=E^{Q}\left[{\left({\tilde{Y}}_{t}-k{\tilde{N}}_{t}\right)}_{+}\right]=E^{Q}\left[R_{t}N_{t}{\left(S_{t}-k\right)}_{+}\right]\\ \; & =E^{Q}\left[{\tilde{N}}_{t}{\left(S_{t}-k\right)}_{+}\right],\;k\in R. \end{array} \end{array}$$

Additionally, ref. [19] (Lemma 3.1) implies that the function $c:R^{2}\to R$ defined by

$$c\left(t,k\right)=c\left(t,k\right)1_{T\times R}\left(t,k\right)$$

is an element of $L_{loc}^{1}\left(R^{2}\right)$ and, thus, $C=\iota_{\left(0,T\right)\times R,R^{2}}\left(c\right)$ belongs to $D^{\prime }$.

The following theorem, derived and proved in [19], presents the generalised version of the Dupire formula.

**Theorem** **1.** *If the processes $\tilde{N}={\left({\tilde{N}}_{t}\right)}_{t\in T}$, $S={\left(S_{t}\right)}_{t\in T}$, and $\tilde{Y}={\left({\tilde{Y}}_{t}\right)}_{t\in T}$ are given by (5), (6), (8) and the inequality (7) holds, then*

(10) $$\begin{array}{c} \begin{array}{cc} \frac{\partial C}{\partial t}= & \frac{1}{2}E^{t}\left(\sigma_{t}^{2}|S_{t-}=k\right)\frac{\partial^{2}C}{\partial k^{2}}+\int_{k}^{\infty }E^{t}\left(\left.\frac{\lambda_{t}}{{\tilde{N}}_{t-}}-\frac{\beta_{t}}{{\tilde{N}}_{t-}}k\right|S_{t-}=x\right)\Phi \left(t,dx\right)\\ \; & +\int_{R}\int_{k}^{\infty }E^{t}\left(\left.\frac{{\tilde{\xi }}^{\left(2\right)}\left(t,y\right)}{{\tilde{N}}_{t-}}-\frac{{\tilde{\hat{k}}}_{1}\left(t,y\right)}{{\tilde{N}}_{t-}}k\right|S_{t-}=x\right)\Phi \left(t,dx\right)\bar{\nu }\left(dy\right)\\ \; & +\int_{R}\int_{R}E^{t}\left(\frac{{\tilde{\hat{h}}}_{1}\left(t,y\right)j\left(S_{t-},{\tilde{\hat{h}}}_{2}\left(t,y\right)\right)\left(k\right)+{\tilde{\hat{k}}}_{1}\left(t,y\right)j\left(S_{t-},{\tilde{\hat{k}}}_{2}\left(t,y\right)\right)\left(k\right)}{{\tilde{N}}_{t-}}\right.\\ \; & \;\;\left.\left.+j\left(S_{t-},{\tilde{\hat{h}}}_{2}\left(t,y\right)\right)\left(k\right)+j\left(S_{t-},{\tilde{\hat{k}}}_{2}\left(t,y\right)\right)\left(k\right)\right|S_{t-}=x\right)\Phi \left(t,dx\right)\bar{\nu }\left(dy\right) \end{array} \end{array}$$

*in $D^{\prime }$, where Φ(t,dx) denotes the finite transition kernel from (T,L(T)) to (R,B(R)) defined by (11), $\frac{\partial^{2}C}{\partial k^{2}}=l⊗\Phi$ is interpreted as the finite measure on (T×R,L(T)⊗B(R)) satisfying (12). The notation $E^{t}$ refers to expectations taken under the probability measures $Q^{t}$ given by*

$$Q^{t}\left(A\right)=E^{Q}\left({\tilde{N}}_{t-}1_{A}\right)/E^{Q}{\tilde{N}}_{t-},\;A\in F,\;t\in T.$$

Moreover, if $\mu_{S_{t-}}^{t}$ is the probability distribution of $S_{t-}$ with respect to $Q^{t}$, then in $D^{\prime }$,

$$\begin{array}{cc} \; & \frac{\partial C}{\partial k}=-E^{Q}{\tilde{N}}_{t-}E^{t}1_{S_{t-}>k}, \end{array}$$

(11) $$\Phi \left(t,dx\right)=E^{Q}{\tilde{N}}_{t-}\mu_{S_{t-}}^{t}\left(dx\right),$$

(12) $$l⊗\Phi \left(A_{1}\times A_{2}\right)=\int_{A_{1}}\Phi \left(t,A_{2}\right)dt,\;A_{1}\in L\left(T\right),\;A_{2}\in B\left(R\right),$$

is a finite measure on (T×R,L(T)⊗B(R)), and $\frac{\partial^{2}C}{\partial k^{2}}=l⊗\Phi$.

**Remark** **1.** *Equation (10) in $D^{\prime }$ can be expressed as follows:*

$$\begin{array}{c} \begin{array}{cc} \; & -\int_{0}^{T}\int_{-\infty }^{\infty }\;f_{t}^{\prime }\left(t,k\right)C\left(t,k\right)dk\;dt\\ \; & =\frac{1}{2}\int_{0}^{T}\int_{-\infty }^{\infty }f\left(t,k\right)E^{t}\left(\sigma_{t}^{2}|S_{t-}=k\right)\frac{\partial^{2}C}{\partial k^{2}}\left(dt\;dk\right)\\ \; & +\int_{0}^{T}\int_{-\infty }^{\infty }f\left(t,k\right)\int_{k}^{\infty }E^{t}\left(\left.\frac{\lambda_{t}}{{\tilde{N}}_{t-}}-\frac{\beta_{t}}{{\tilde{N}}_{t-}}k\right|S_{t-}=x\right)\Phi \left(t,dx\right)\;dk\;dt\\ \; & +\int_{0}^{T}\int_{-\infty }^{\infty }f\left(t,k\right)\int_{R}\int_{k}^{\infty }E^{t}\\ \; & \;\;\;\;\;\;\;\;\;\;\;\;\left(\left.\frac{{\tilde{\xi }}^{\left(2\right)}\left(t,y\right)}{{\tilde{N}}_{t-}}-\frac{{\tilde{\hat{k}}}_{1}\left(t,y\right)}{{\tilde{N}}_{t-}}k\right|S_{t-}=x\right)\Phi \left(t,dx\right)\bar{\nu }\left(dy\right)\;dk\;dt\\ \; & +\int_{0}^{T}\int_{-\infty }^{\infty }f\left(t,k\right)\int_{R}\int_{R}E^{t}\\ \; & \;\;\;\;\;\;\;\;\;\;\;\;\left(\frac{{\tilde{\hat{h}}}_{1}\left(t,y\right)j\left(S_{t-},{\tilde{\hat{h}}}_{2}\left(t,y\right)\right)\left(k\right)+{\tilde{\hat{k}}}_{1}\left(t,y\right)j\left(S_{t-},{\tilde{\hat{k}}}_{2}\left(t,y\right)\right)\left(k\right)}{{\tilde{N}}_{t-}}\right.\\ \; & \;\;\;\;\;\;\;\;\;\;\;\;\;\left.\left.+j\left(S_{t-},{\tilde{\hat{h}}}_{2}\left(t,y\right)\right)\left(k\right)+j\left(S_{t-},{\tilde{\hat{k}}}_{2}\left(t,y\right)\right)\left(k\right)\right|S_{t-}=x\right)\Phi \left(t,dx\right)\bar{\nu }\left(dy\right)\;dk\;dt \end{array} \end{array}$$

*for every R-valued $C^{\infty }$-function f on $R^{2}$ of compact support in (0,T)×R.*

## 3. The Existence and Uniqueness of a Solution of a Generalised Stochastic Differential Equation

This section is devoted to the proof of the existence and uniqueness of the solution of a general stochastic differential equation describing the underlying asset price. We consider the same filtered probability space as in the previous section. The probability measure Q will be interpreted as a risk-neutral measure in the next section. Simultaneously, the drift term of the SDE under consideration contains the short rate to ensure that the discounted price process is a martingale under the risk-neutral probability measure. This structure will be used in the arbitrage-free valuation of a European call option in the following part of the paper.

### 3.1. Functional Framework

We will assume that p≥2 and p∈N, a convention that simplifies several estimates appearing in the proofs.

Let $\Lambda^{p}$ be the space of càdlàg and adapted stochastic processes ${\left(X\right)}_{t\in T}$, identified up to indistinguishability, such that $E[\sup_{t\in T}|X_{t}{|}^{p}]<\infty$.

$\Lambda^{p}$ with the norm ${∥X∥}_{\Lambda^{p}}=(E[\sup_{t\in T}|X_{t}{|}^{p}{])}^{1/p}$ is a Banach space, which is a consequence of the Minkowski inequality, the Hölder inequality, the Lebesgue monotone convergence theorem, and preserving the càdlàg property by uniform limits on T.

Similarly, for every closed interval I=[a,b]⊂T, we define the space $\Lambda^{p}\left(I\right)$ of càdlàg and adapted stochastic processes ${\left(X\right)}_{t\in I}$, identified up to indistinguishability, such that $E[\sup_{t\in I}|X_{t}{|}^{p}]<\infty$. Then, the space $\Lambda^{p}\left(I\right)$ with the norm ${∥X∥}_{\Lambda^{p}\left(I\right)}^{p}=(E[\sup_{t\in I}|X_{t}{|}^{p}{])}^{1/p}$ is a Banach space (since it is isometrically isomorphic to a closed subspace of $\Lambda^{p}$).

We will use the following form of the Gronwall inequality (see [29], Proposition 6.1.4).

**Proposition** **1.** *Let [a,b]⊂R, α:[a,b]→R be non-negative and locally bounded, β:[a,b]→R be non-negative and integrable, C≥0 be a constant, and $\alpha \left(t\right)\leq C+\int_{a}^{t}\alpha \left(s\right)\beta \left(s\right)ds,\;t\in \left[a,b\right]$. Then, $\alpha \left(t\right)\leq Ce^{\int_{a}^{t}\beta \left(s\right)ds},\;t\in \left[a,b\right]$.*

### 3.2. Assumptions

Throughout this section, the considered processes and measures X, $W^{\left(2\right)}$, N, *q*, and $\bar{\nu }$ are those defined in Section 2. The SDE introduced below is formulated for a real-valued state process *S*. An interpretation of *S* as an asset price requires additional positivity assumptions, which are satisfied in the exponential-jump models of Section 4.

We consider the following SDE

(13) $$\begin{array}{c} \begin{array}{cc} dS_{t} & =S_{t-}r_{t-}dt+S_{t-}\sigma \left(t,S_{t-}\right)dW_{t}^{\left(2\right)}+\int_{R}\tilde{h}\left(t,S_{t-},y\right)q\left(dt\;dy\right)+\int_{R}\tilde{k}\left(t,S_{t-},y\right)q\left(dt\;dy\right), \end{array} \end{array}$$

with initial condition $S_{0}>0$, where $\tilde{h}=h1_{\left\{|y|<1\right\}}$ and $\tilde{k}=k1_{\left\{|y|\geq 1\right\}}$. We impose the following assumptions.

(i) The Lévy measure $\bar{\nu }$ is finite.
(ii) The map (t,x)↦σ(t,x):T×R→R is Borel measurable. Moreover, there exists a constant $L_{\sigma }>0$ such that for all $x,x_{1}\in R$,
  (14) $$|x\sigma \left(t,x\right)-x_{1}\sigma \left(t,x_{1}\right)|\leq L_{\sigma }\left|x-x_{1}\right|.$$
  In addition, we assume one of the following growth conditions:
  (a) There exists a constant $C_{\sigma }>0$ such that
    (15) $${\left|x\sigma \left(t,x\right)\right|}^{p}\leq C_{\sigma }\left(1+{\left|x\right|}^{p}\right).$$
  (b) There exists a constant $C_{\sigma }^{\prime }>0$ such that
    (16) $${\left|x\sigma \left(t,x\right)\right|}^{2p}\leq C_{\sigma }^{\prime }\left(1+{\left|x\right|}^{2p}\right).$$
    Since for $C_{\sigma }={\left(C_{\sigma }^{\prime }\right)}^{1/2}$,
    $$\begin{array}{c} {\left|x\sigma \left(t,x\right)\right|}^{p}={\left({\left|x\sigma \left(t,x\right)\right|}^{2p}\right)}^{1/2}\leq {\left(C_{\sigma }^{\prime }\right)}^{1/2}{\left(1+{\left|x\right|}^{2p}\right)}^{1/2}\leq C_{\sigma }\left(1+{\left|x\right|}^{p}\right), \end{array}$$
    (b) implies (a).
(iii) The functions $h,k:T\times R^{2}\times \Omega \to R$,(t,x,y,ω)↦h(t,x,y,ω),k(t,x,y,ω) are predictable, and thus, their truncated versions $\tilde{h}\left(t,x,y,\omega \right)=h\left(t,x,y,\omega \right)1_{\left\{|y|<1\right\}}$
  and $\tilde{k}\left(t,x,y,\omega \right)$$=k\left(t,x,y,\omega \right)1_{\left\{|y|\geq 1\right\}}$ are also predictable. Furthermore, there exist Borel-measurable functions $K^{\left(h\right)},K^{\left(k\right)},L^{\left(h\right)},L^{\left(k\right)}:R\to \left[0,\infty \right)$ such that for each $x,x_{1},y\in R$, and (l⊗Q)-a.e.(t,ω),
  (17) $$\left|h\left(t,x,y,\omega \right)\right|\leq K^{\left(h\right)}\left(y\right)(1+|x|),\;|h\left(t,x,y,\omega \right)-h\left(t,x_{1},y,\omega \right)|\leq L^{\left(h\right)}\left(y\right)\left|x-x_{1}\right|,$$
  (18) $$\left|k\left(t,x,y,\omega \right)\right|\leq K^{\left(k\right)}\left(y\right)(1+|x|),\;|k\left(t,x,y,\omega \right)-k\left(t,x_{1},y,\omega \right)|\leq L^{\left(k\right)}\left(y\right)\left|x-x_{1}\right|.$$
  We assume one of the following integrability conditions:
  (a) (19) $$K^{\left(h\right)},K^{\left(k\right)},L^{\left(h\right)},L^{\left(k\right)}\in L^{p}\left(\bar{\nu }\right).$$
  (b) (20) $$K^{\left(h\right)},K^{\left(k\right)},L^{\left(h\right)},L^{\left(k\right)}\in L^{2p}\left(\bar{\nu }\right),$$
    and therefore, (b) implies (a).
  Throughout this section, we suppress the argument ω and write h(t,x,y), $\tilde{h}\left(t,x,y\right)$, k(t,x,y), $\tilde{k}\left(t,x,y\right)$ whenever no confusion may arise.
(iv) The short rate process $r={\left(r_{t}\right)}_{t\in T}$ is adapted and càdlàg.

**Assumption** **1.** *Assumption 1 consists of items (i)–(iv) above, with the growth condition of order p in (ii.a) and the integrability condition of order p in (iii.a).*

**Assumption** **2.** *Assumption 2 consists of items (i)–(iv) above, with the growth condition of order 2p in (ii.b) and the integrability condition of order 2p in (iii.b).*

Additionally, we will write $b\left(x,s\right)=xr_{s-}$,f(x,s)=xσ(s,x),g=h+k, and $\tilde{g}=\tilde{h}+\tilde{k}$, and consider the following tail condition

(21) $$Q\left(\underset{t\leq T}{\sup}\left|r_{t}\right|>x\right)\leq C_{r}e^{-c_{r}x^{\beta }},\;x\geq 0,$$

for some constants $C_{r}>0$, $c_{r}>0$, and β>1.

Throughout this paper, we do not assume any specific dependence or independence structure between the short-rate process *r* and the other stochastic processes appearing in the model (the Brownian motions $W^{\left(1\right)}$, $W^{\left(2\right)}$ or the Lévy process X).

### 3.3. Preparatory Results

We also introduce weaker versions of the tail condition, stated in the next proposition.

**Proposition** **2.** *Assumption *(21)* implies that for every q≥1,*

(22) $$E\left[\underset{t\leq T}{\sup}{\left|r_{t}\right|}^{q}\right]<\infty$$

*and*

(23) $$\int_{0}^{T}E{\left|r_{t}\right|}^{q}dt<\infty .$$

Tail assumption (21) is a sufficient condition used to obtain the exponential-type integrability needed in the moment estimates.

The following lemma will be useful in the next section.

**Lemma** **1.** *Let ${\left(r_{t}\right)}_{t\in T}$ be an adapted càdlàg process satisfying *(21)* and $R_{t}=\exp\left(-\int_{0}^{t}r_{s}ds\right)$, t∈T. Then, for every q≥1, ${\left(R_{t}\right)}_{t\in T}\in \Lambda^{q}$.*

For the proofs of Proposition 2 and Lemma 1, we refer the reader to Appendix A. Equation (13) can also be written in the following form using Lévy-type stochastic integrals considered in [29], where one of the compensators of jumps is absorbed into the drift. Both formulations are equivalent.

(24) $$\begin{array}{c} \begin{array}{cc} S_{t} & =S_{0}+\int_{0}^{t}S_{s-}r_{s-}ds+\int_{0}^{t}S_{s-}\sigma \left(s,S_{s-}\right)dW_{s}^{\left(2\right)}+\int_{0}^{t}\int_{R}\tilde{h}\left(s,S_{s-},y,\omega \right)q\left(ds\;dy\right)\\ \; & +\int_{0}^{t}\int_{R}\tilde{k}\left(s,S_{s-},y,\omega \right)N\left(dsdy\right)-\int_{0}^{t}\int_{R}\tilde{k}\left(s,S_{s-},y,\omega \right)\bar{\nu }\left(dy\right)ds \end{array} \end{array}$$

with initial condition $S_{0}>0$.

We will also use the notation (cf. [30])

$$\int_{0}^{t}X\left(\theta_{s-},ds\right)=\int_{0}^{t}b\left(\theta_{s-},s\right)ds+\int_{0}^{t}f\left(\theta_{s-},s\right)dW_{s}^{\left(2\right)}+\int_{0}^{t}\int_{R}\tilde{g}\left(s,\theta_{s-},y\right)q\left(ds\;dy\right),$$

where the process θ is adapted and càdlàg. The following lemma will be useful in our further considerations.

**Lemma** **2.** *Let Assumption 1 and *(22)* with q=1 be satisfied, and θ be an adapted and càdlàg stochastic process. Then, the stochastic process $\int_{0}^{t}X\left(\theta_{s-},ds\right)$, t∈T is well defined, and it is a semimartingale on T.*

For the proof, we refer the reader to Appendix A.

The preceding lemma enables writing (13) in the form

(25) $$\begin{array}{c} S_{t}=S_{0}+\int_{0}^{t}X\left(S_{s-},ds\right) \end{array}$$

with initial condition $S_{0}>0$. If Assumption 1 is satisfied, then for all x∈R and (l⊗Q)-a.e. (t,ω),

(26) $$\begin{array}{c} \begin{array}{cc} \; & {\left|x\sigma \left(t,x\right)\right|}^{p}+\int_{R}{|\tilde{h}\left(t,x,y\right)|}^{p}\bar{\nu }\left(dy\right)+\int_{R}{\left|\tilde{k}\left(t,x,y\right)\right|}^{p}\bar{\nu }\left(dy\right)\leq C_{\sigma }\left(1+{\left|x\right|}^{p}\right)\\ \; & +\int_{R}{\left(K^{\left(h\right)}\left(y\right)\right)}^{p}{\left(1+|x|\right)}^{p}\bar{\nu }\left(dy\right)+\int_{R}{\left(K^{\left(k\right)}\left(y\right)\right)}^{p}{\left(1+|x|\right)}^{p}\bar{\nu }\left(dy\right)\leq C_{\sigma }\left(1+{\left|x\right|}^{p}\right)\\ \; & +\int_{R}2^{p-1}{\left(K^{\left(h\right)}\left(y\right)\right)}^{p}{\left(1+|x\right|}^{p})\bar{\nu }\left(dy\right)+\int_{R}2^{p-1}{\left(K^{\left(k\right)}\left(y\right)\right)}^{p}{\left(1+|x\right|}^{p})\bar{\nu }\left(dy\right)\leq C_{p}\left(1+{\left|x\right|}^{p}\right), \end{array} \end{array}$$

where $C_{p}=C_{\sigma }+C_{h}+C_{k}$, $C_{h}=2^{p-1}\int_{R}{\left(K^{\left(h\right)}\left(y\right)\right)}^{p}\bar{\nu }\left(dy\right)$, and $C_{k}=2^{p-1}\int_{R}{\left(K^{\left(k\right)}\left(y\right)\right)}^{p}\bar{\nu }\left(dy\right)$. In turn, if Assumption 2 holds, then for all x∈R and (l⊗Q)-a.e. (t,ω),

(27) $$\begin{array}{c} \begin{array}{cc} \; & {\left|x\sigma \left(t,x\right)\right|}^{2p}+\int_{R}{|\tilde{h}\left(t,x,y\right)|}^{2p}\bar{\nu }\left(dy\right)+\int_{R}{\left|\tilde{k}\left(t,x,y\right)\right|}^{2p}\bar{\nu }\left(dy\right)\leq C_{2p}\left(1+{\left|x\right|}^{2p}\right), \end{array} \end{array}$$

with $C_{2p}=C_{\sigma }^{\prime }+C_{h}^{\prime }+C_{k}^{\prime }$, $C_{h}^{\prime }=2^{2p-1}\int_{R}{\left(K^{\left(h\right)}\left(y\right)\right)}^{2p}\bar{\nu }\left(dy\right)$, $C_{k}^{\prime }=2^{2p-1}\int_{R}{\left(K^{\left(k\right)}\left(y\right)\right)}^{2p}\bar{\nu }\left(dy\right)$.

We present the lemmas used in the following part of this section.

**Lemma** **3.** *Let $M={\left(M_{t}\right)}_{t\in T}$ be a càdlàg process. Then, $\sup_{s_{1}\leq t\leq s_{2}}|M_{t}|=\sup_{s_{1}<t\leq s_{2}}\left|M_{t}\right|$ a.s. for any deterministic times $0\leq s_{1}<s_{2}\leq T$.*

In all formulas in the next two lemmas and remarks, every upper limit *t* in integrals and in expressions of the form $\sup_{t_{*}<s\leq t}$ or $\sup_{t_{*}\leq s\leq t}$ is assumed to satisfy 0<t≤T.

We now state the version of [30] (Lemma 2.11) adapted to our setting.

**Lemma** **4.** *Let p≥2 and $Y\left(t\right)=\int_{0}^{t}\int_{R}g\left(r,z\right)q\left(dr\;dz\right)$, where g is predictable and*

$$E\left[{\left(\int_{0}^{t}\int_{R}{\left|g\left(r,z\right)\right|}^{2}\bar{\nu }\left(dz\right)dr\right)}^{p/2}\right]+E\left[\int_{0}^{t}\int_{R}{\left|g\left(r,z\right)\right|}^{p}\bar{\nu }\left(dz\right)dr\right]<\infty .$$

*Then, there exist constants $c_{7},c_{8}>0$ depending only on p such that*

(28) $$\begin{array}{c} E\left[\underset{0<s\leq t}{\sup}{\left|Y\left(s\right)\right|}^{p}\right]\leq c_{7}E\left[{\left(\int_{0}^{t}\int_{R}{\left|g\left(r,z\right)\right|}^{2}\nu \left(dz\right)dr\right)}^{p/2}\right]+c_{8}E\left[\int_{0}^{t}\int_{R}{\left|g\left(r,z\right)\right|}^{p}\nu \left(dz\right)dr\right]. \end{array}$$

**Remark** **2.** *The inequality *(28)* remains valid under stopping and under deterministic time-shift, in the following sense.*

*(i)*  *Let for any stopping time τ, $Y^{\tau }\left(t\right)=\int_{0}^{t}\int_{R}g\left(r,z\right)1_{\left\{r\leq \tau \right\}}q\left(dr\;dz\right)=\int_{0}^{t\wedge \tau }\int_{R}g\left(r,z\right)q\left(dr\;dz\right)$. The process $r\mapsto 1_{\left\{r\leq \tau \right\}}$ is predictable (adapted and left-continuous); hence, $g\left(r,z\right)1_{\left\{r\leq \tau \right\}}$ is predictable. Additionally, $\int_{0}^{t}\int_{R}{\left|g\left(r,z\right)\right|}^{2}1_{\left\{r\leq \tau \right\}}\bar{\nu }\left(dz\right)dr\leq \int_{0}^{t}\int_{R}{\left|g\left(r,z\right)\right|}^{2}\bar{\nu }\left(dz\right)dr$, and similarly for the p-th power. Therefore, inequality *(28)* applies to $Y^{\tau }$ with the same constants.*
*(ii)*  Let $t^{*}\geq 0$, $t>t^{*}$. We define the process $Y_{s}^{\left(t^{*}\right)}=\int_{t^{*}}^{s}\int_{R}g\left(r,z\right)q\left(dr\;dz\right),\;s\geq t^{*}$. Equivalently,
$$Y_{s}^{\left(t^{*}\right)}=\int_{0}^{s}\int_{R}g\left(r,z\right)1_{\left(t^{*},T\right]}\left(r\right)q\left(dr\;dz\right),$$
  and the integrand $g\left(r,z\right)1_{\left(t^{*},T\right]}\left(r\right)$ is predictable. Since $Y^{\left(t^{*}\right)}$ is càdlàg, Lemma 3 implies
$$\underset{t^{*}<s\leq t}{\sup}\left|Y_{s}^{\left(t^{*}\right)}\right|=\underset{t^{*}\leq s\leq t}{\sup}\left|Y_{s}^{\left(t^{*}\right)}\right|\;a.s.$$
By applying Lemma 4 to $Y^{\left(t^{*}\right)}$, we obtain
$$\begin{array}{cc} E\left[\underset{t^{*}<s\leq t}{\sup}{\left|Y_{s}^{\left(t^{*}\right)}\right|}^{p}\right]\; & =E\left[\underset{t^{*}\leq s\leq t}{\sup}{\left|Y_{s}^{\left(t^{*}\right)}\right|}^{p}\right]\leq c_{7}E\left[{\left(\int_{t^{*}}^{t}\int_{R}{\left|g(r,z)\right|}^{2}\bar{\nu }\left(dz\right)dr\right)}^{p/2}\right]\\ \; & +c_{8}E\left[\int_{t^{*}}^{t}\int_{R}{\left|g(r,z)\right|}^{p}\bar{\nu }\left(dz\right)dr\right]. \end{array}$$

The following form of the Kunita-type inequality is also used in our considerations.

**Lemma** **5.** *Let p≥2 and g be a predictable function such that*

$$\int_{0}^{t}\int_{R}{\left|g\left(s,y,\omega \right)\right|}^{2}\bar{\nu }\left(dy\right)ds<\infty ,\;\int_{0}^{t}\int_{R}{\left|g\left(s,y,\omega \right)\right|}^{p}\bar{\nu }\left(dy\right)ds<\infty \;a.s.$$

*and $Y\left(s\right)=\int_{0}^{s}\int_{R}g\left(r,y\right)q\left(dr\;dy\right)$,s∈T. Then, there exist constants $c_{7},c_{8}>0$ depending only on p such that the following inequality holds in [0,∞]*

(29) $$E\left[\underset{0<s\leq t}{\sup}{\left|Y\left(s\right)\right|}^{p}\right]\leq c_{7}E\left[{\left(\int_{0}^{t}\int_{R}{\left|g\left(r,y\right)\right|}^{2}\bar{\nu }\left(dy\right)dr\right)}^{p/2}\right]+c_{8}E\left[\int_{0}^{t}\int_{R}{\left|g\left(r,y\right)\right|}^{p}\bar{\nu }\left(dy\right)dr\right].$$

**Remark** **3.** *The estimate of Lemma 5 can be used to stopped and time-shifted versions of the stochastic integral, as follows.*

*(i)*  *If τ is a stopping time, then $Y^{\tau }\left(s\right)=\int_{0}^{s}\int_{R}g\left(r,y\right)1_{\left\{r\leq \tau \right\}}q\left(dr\;dy\right)$ is also a process of the same form, and Lemma 5 applies to $Y^{\tau }$ with the same constants.*
*(ii)*  Let $t^{*}\geq 0$, $t>t^{*}$. We consider the process defined for $s\geq t^{*}$ by
$$Y_{s}^{\left(t^{*}\right)}=\int_{t^{*}}^{s}\int_{R}g\left(r,z\right)q\left(dr\;dz\right).$$
*Equivalently,*
$$Y_{s}^{\left(t^{*}\right)}=\int_{0}^{s}\int_{R}g\left(r,z\right)1_{\left(t^{*},T\right]}\left(r\right)q\left(dr\;dz\right),$$
  where the integrand $g\left(r,z\right)1_{\left(t^{*},T\right]}\left(r\right)$ is predictable. Since $Y^{\left(t^{*}\right)}$ is càdlàg, Lemma 3 yields
$$\underset{t^{*}<s\leq t}{\sup}\left|Y_{s}^{\left(t^{*}\right)}\right|=\underset{t^{*}\leq s\leq t}{\sup}\left|Y_{s}^{\left(t^{*}\right)}\right|\;a.s.$$
Applying Lemma 5 to the process $Y^{\left(t^{*}\right)}$ gives
$$\begin{array}{cc} E\left[\underset{t^{*}<s\leq t}{\sup}{\left|Y_{s}^{\left(t^{*}\right)}\right|}^{p}\right]\; & =E\left[\underset{t^{*}\leq s\leq t}{\sup}{\left|Y_{s}^{\left(t^{*}\right)}\right|}^{p}\right]\leq c_{7}E\left[{\left(\int_{t^{*}}^{t}\int_{R}{\left|g(r,z)\right|}^{2}\bar{\nu }\left(dz\right)dr\right)}^{p/2}\right]\\ \; & +c_{8}E\left[\int_{t^{*}}^{t}\int_{R}{\left|g(r,z)\right|}^{p}\bar{\nu }\left(dz\right)dr\right]. \end{array}$$

**Lemma** **6.** *Let Assumption 1 be satisfied. Then, there exists a constant ${\hat{C}}_{p}>0$ such that for x∈R and (l⊗Q)-a.e. (s,ω),*

$$\begin{array}{c} \begin{array}{cc} & \int_{R}{\left|x\right|}^{p-2}{|\tilde{h}(s,x,y,\omega )|}^{2}\bar{\nu }\left(dy\right)\vee \int_{R}{\left|x\right|}^{p-2}{\left|\tilde{k}\left(s,x,y,\omega \right)\right|}^{2}\bar{\nu }\left(dy\right)\leq {\hat{C}}_{p}\left(1+{\left|x\right|}^{p}\right). \end{array} \end{array}$$

*If Assumption 2 holds, then there exists a constant ${\hat{C}}_{2p}>0$ such that for x∈R and (l⊗Q)-a.e. (s,ω),*

$$\begin{array}{c} \begin{array}{cc} & \int_{R}{\left|x\right|}^{2p-2}{|\tilde{h}(s,x,y,\omega )|}^{2}\bar{\nu }\left(dy\right)\vee \int_{R}{\left|x\right|}^{2p-2}{\left|\tilde{k}\left(s,x,y,\omega \right)\right|}^{2}\bar{\nu }\left(dy\right)\leq {\hat{C}}_{2p}\left(1+{\left|x\right|}^{2p}\right). \end{array} \end{array}$$

**Lemma** **7.** *Let q≥2. Then, there exists a constant ${\overset{ˇ}{C}}_{q}>0$ such that for all x,u∈R,*

$$\left|{\left|x+u\right|}^{q}-{\left|x\right|}^{q}\right|\leq {\overset{ˇ}{C}}_{q}\left({\left|x\right|}^{q-1}\left|u\right|+{\left|u\right|}^{q}\right).$$

For the proofs of Lemmas 3, 5, 6, and 7, we refer the reader to Appendix A.

We employ the technique of Picard iteration (see, e.g., [29]), considering the stopping times $\rho_{M}=\inf\{t\geq 0:|r_{t}\left|\geq M\right\}\wedge T$,M∈N, fixing a time step δ>0, and defining the time partition points: $t_{k}=\min\left(k\delta ,T\right)$,k∈[0:N], where $N=\left\lceil \frac{T}{\delta }\right\rceil$. Moreover, for k∈[0:N−1], we chose $Y_{k}\in \Lambda^{p}\left(\left[0,t_{k}\right]\right)$ with the convention $Y_{k}\left(0-\right)=Y_{k}\left(0\right)$, and introduce the notation $\xi_{k}=Y_{k}\left(t_{k}\right)$. In particular, $\xi_{0}\left(\omega \right)=Y_{0}\left(t_{0},\omega \right)=S_{0},\;\omega \in \Omega$. For k∈[1:N−1], the process $Y_{k}$ will be introduced later in the proof, as part of a recursive construction in which $Y_{k}$ is glued to the Picard limit obtained on the next interval $\left[t_{k},t_{k+1}\right]$. We construct the solution by a successive approximation on the above partition. We define recursively Picard iterates on $\left[0,t_{k+1}\right]$, for k∈[0:N−1],

$$\begin{array}{cc} \; & S_{t}^{\left(0,M,k\right)}=Y_{k}\left(t\right),\;t\in \left[0,t_{k}\right)\;\mathrm{and}\;S_{t}^{\left(0,M,k\right)}=\xi_{k},\;t\in \left[t_{k},t_{k+1}\right],\\ \; & S_{t}^{\left(n+1,M,k\right)}=Y_{k}\left(t\right),\;t\in \left[0,t_{k}\right),\;S_{t}^{\left(n+1,M,k\right)}=\xi_{k}+\int_{t_{k}}^{t\wedge \rho_{M}}X\left(S_{s-}^{\left(n,M,k\right)},ds\right),\;t\in \left[t_{k},t_{k+1}\right],\;n\geq 0, \end{array}$$

where $\int_{t_{k}}^{t\wedge \rho_{M}}X\left(S_{s-}^{\left(n,M,k\right)},ds\right)=A_{t}^{\left(n,M,k\right)}+M_{t}^{\left(B,n,M,k\right)}+M_{t}^{\left(J,n,M,k\right)}+M_{t}^{\left(K,n,M,k\right)}$,

$$\begin{array}{cc} \; & A_{t}^{\left(n,M,k\right)}=\int_{t_{k}}^{t\wedge \rho_{M}}b\left(S_{s-}^{\left(n,M,k\right)},s\right)ds,\;M_{t}^{\left(B,n,M,k\right)}=\int_{t_{k}}^{t\wedge \rho_{M}}f\left(S_{s-}^{\left(n,M,k\right)},s\right)dW_{s}^{\left(2\right)},\\ \; & M_{t}^{\left(J,n,M,k\right)}=\int_{t_{k}}^{t\wedge \rho_{M}}\int_{R}\tilde{h}\left(s,S_{s-}^{\left(n,M,k\right)},y\right)q\left(ds\;dy\right),\\ \; & M_{t}^{\left(K,n,M,k\right)}=\int_{t_{k}}^{t\wedge \rho_{M}}\int_{R}\tilde{k}\left(s,S_{s-}^{\left(n,M,k\right)},y\right)q\left(ds\;dy\right),\;t\in \left[t_{k},t_{k+1}\right],\;k,n\geq 0, \end{array}$$

and thus, $S_{t}^{\left(n+1,M,k\right)}=S_{t\wedge \rho_{M}}^{\left(n+1,M,k\right)},\;t\in \left[t_{k},t_{k+1}\right],\;S_{t_{k}-}^{\left(n,M,k\right)}=Y_{k}\left(t_{k}-\right)$, n≥0,k∈[0:N−1]. We also write ${\hat{S}}^{\left(n,M,k\right)}=S^{\left(n,M,k\right)}{|}_{\left[t_{k},t_{k+1}\right]}$,n≥0,k∈[0:N−1].

For k∈[0:N−1] and n≥0, we define the Picard differences $D_{t}^{\left(n,M,k\right)}=$$S_{t\wedge \rho_{M}}^{\left(n+1,M,k\right)}-S_{t\wedge \rho_{M}}^{\left(n,M,k\right)}$,$t\in [t_{k},t_{k+1}]$, adopting the convention $D_{t_{k}-}^{\left(n,M,k\right)}=D_{t_{k}}^{\left(n,M,k\right)}=0$.

Thus, $D_{t}^{\left(0,M,k\right)}=S_{t}^{\left(1,M,k\right)}-S_{t}^{\left(0,M,k\right)}$, $D_{t}^{\left(n,M,k\right)}=A_{t}^{\left(D,n,M,k\right)}+M_{t}^{\left(B,D,n,M,k\right)}+M_{t}^{\left(J,D,n,M,k\right)}$$+M_{t}^{\left(K,D,n,M,k\right)}$$=A_{t}^{\left(D,n,M,k\right)}+M_{t}^{\left(B,D,n,M,k\right)}+M_{t}^{\left(J,K,D,n,M,k\right)}$, $t\in [t_{k},t_{k+1}]$, n≥1, with

$$A_{t}^{\left(D,n,M,k\right)}=\int_{t_{k}}^{t\wedge \rho_{M}}\left[b\left(S_{s-}^{\left(n\right)},s\right)-b\left(S_{s-}^{\left(n-1\right)},s\right)\right]ds,$$

$$M_{t}^{\left(B,D,n\right)}=\int_{t_{k}}^{t\wedge \rho_{M}}\left[f\left(S_{s-}^{\left(n\right)},s\right)-f\left(S_{s-}^{\left(n-1\right)},s\right)\right]dW_{s}^{\left(2\right)},$$

$$M_{t}^{\left(J,D,n,M,k\right)}=\int_{t_{k}}^{t\wedge \rho_{M}}\int_{R}\left[\tilde{h}\left(s,S_{s-}^{\left(n\right)},y\right)-\tilde{h}\left(s,S_{s-}^{\left(n-1\right)},y\right)\right]q\left(ds\;dy\right),$$

$$M_{t}^{\left(K,D,n,M,k\right)}=\int_{t_{k}}^{t\wedge \rho_{M}}\int_{R}\left[\tilde{k}\left(s,S_{s-}^{\left(n\right)},y\right)-\tilde{k}\left(s,S_{s-}^{\left(n-1\right)},y\right)\right]q\left(ds\;dy\right),$$

$$M_{t}^{\left(J,K,D,n,M,k\right)}=\int_{t_{k}}^{t\wedge \rho_{M}}\int_{R}H^{\left(J,K,D,n,M,k\right)}\left(s,y\right)q\left(ds\;dy\right),$$

$$\begin{array}{cc} H^{\left(J,K,D,n,M,k\right)}\left(s,y\right) & =\tilde{g}\left(s,S_{s-}^{\left(n\right)},y\right)-\tilde{g}\left(s,S_{s-}^{\left(n-1\right)},y\right). \end{array}$$

For notational convenience, when *M* and *k* are fixed, we write $S^{\left(n\right)}$, ${\hat{S}}^{\left(n\right)}$, $A^{\left(n\right)}$, $M^{\left(B,n\right)}$, $M^{\left(J,n\right)}$, $M^{\left(K,n\right)}$, $D^{\left(n\right)}$, $A^{\left(D,n\right)}$, $M^{\left(B,D,n\right)}$, $M^{\left(J,D,n\right)}$, $M^{\left(K,D,n\right)}$, $M^{\left(J,K,D,n\right)}$, and $H^{\left(J,K,D,n\right)}$ instead of $S^{\left(n,M,k\right)}$, ${\hat{S}}^{\left(n,M,k\right)}$, $A^{\left(n,M,k\right)}$, $M^{\left(B,n,M,k\right)}$, $M^{\left(J,n,M,k\right)}$, $M^{\left(K,n,M,k\right)}$, $D^{\left(n,M,k\right)}$, $D^{\left(n,M,k\right)}$, $A^{\left(D,n,M,k\right)}$, $M^{\left(B,D,n,M,k\right)}$, $M^{\left(J,D,n,M,k\right)}$, $M^{\left(K,D,n,M,k\right)}$, $M^{\left(J,K,D,n,M,k\right)}$, and $H^{\left(J,K,D,n,M,k\right)}$, n≥1.

### 3.4. Existence, Uniqueness and $\Lambda^{p}$-Properties

Under the assumptions formulated in the lemma below, the Picard iteration is constructed in the fixed filtration ${\left(F_{t}\right)}_{t\in T}$ using the given Brownian motion and compensated Poisson random measure. Hence, the resulting solution is strong, and the usual contraction argument yields pathwise uniqueness.

**Lemma** **8.** *Let Assumption 1 and *(22)* with q=1 be satisfied. Then, *(13)* admits a unique adapted càdlàg solution S on T. Moreover, for each M∈N, the process $S^{\left(M\right)}$ constructed in the proof is the adapted càdlàg solution to the SDE *(13)* stopped at the localisation time $\rho_{M}$, and it satisfies $S^{\left(M\right)}\in \Lambda^{p}$.*

**Proof.**  For n≥1 and $t\in [t_{k},t_{k+1}]$, by the Jensen inequality,

$$\begin{array}{c} \begin{array}{cc} |A_{t}^{\left(n\right)}{|}^{p} & ={\left|\int_{t_{k}}^{t\wedge \rho_{M}}S_{s-}^{\left(n\right)}r_{s-}ds\right|}^{p}\leq M^{p}\delta^{p-1}\int_{t_{k}}^{t_{k+1}}\left(1+{\left|S_{s-}^{\left(n\right)}\right|}^{p}\right)ds=M^{p}\delta^{p-1}\\ \; & \cdot \int_{t_{k}}^{t_{k+1}}1_{\left(t_{k},t_{k+1}\right]}\left(s\right)\left(1+{\left|S_{s-}^{\left(n\right)}\right|}^{p}\right)ds\leq M^{p}\delta^{p}\left(1+\underset{t_{k}\leq u\leq t_{k+1}}{\sup}{\left|{\hat{S}}_{u}^{\left(n\right)}\right|}^{p}\right), \end{array} \end{array}$$

where the last inequality follows from the fact that for each $s\in [t_{k},t_{k+1}]$, we have $1_{\left(t_{k},t_{k+1}\right]}\left(s\right)(1+|S_{s-}^{\left(n\right)}{|}^{p})\leq 1+\sup_{t_{k}\leq u\leq t_{k+1}}{\left|{\hat{S}}_{u}^{\left(n\right)}\right|}^{p}$ a.s. Therefore,

(30) $$\begin{array}{c} \begin{array}{c} E\left[\underset{t_{k}\leq t\leq t_{k+1}}{\sup}{\left|A_{t}^{\left(n\right)}\right|}^{p}\right]\leq M^{p}\delta^{p}\left(1+B_{M,k}^{\left(n\right)}\right), \end{array} \end{array}$$

where $B_{M,k}^{\left(n\right)}=E[\sup_{t_{k}\leq t\leq t_{k+1}}{|{\hat{S}}_{t}^{\left(n,M,k\right)}|}^{p}]$ and expectations are understood as taking values in [0,∞]. At this stage, finiteness of $B_{M,k}^{\left(n\right)}$ is not assumed (but proved recursively below). Since $S^{\left(n,M,k\right)}$ is càdlàg, $\sup_{t_{k}\leq s\leq t_{k+1}}\left(1+\right|S_{s-}^{\left(n,M,k\right)}\left(\omega \right){|}^{p})<\infty$ a.s. and by (26), $\int_{t_{k}}^{t_{k+1}}|S_{s-}^{\left(n,M,k\right)}\sigma \left(s,S_{s-}^{\left(n,M,k\right)}\left(\omega \right)\right){|}^{2}ds\leq C_{p}^{2/p}\int_{t_{k}}^{t_{k+1}}\left(1+\right|S_{s-}^{\left(n,M,k\right)}{{|}^{p})}^{2/p}ds<\infty$ a.s. Clearly, the integrand of $M^{\left(B,n\right)}$ is predictable. Thus, $M^{\left(B,n\right)}$ is a continuous local martingale and therefore, by the BDG inequality (see, e.g., [31] (Thm. 23.12)) with a constant $C_{p}^{B}>0$ and by the Jensen inequality in time, and for $\Gamma_{p}^{\left(B\right)}=C_{p}^{B}C_{p}$, we have

(31) $$\begin{array}{c} \begin{array}{cc} E & \left[\underset{t\in [t_{k},t_{k+1}]}{\sup}{\left|M_{t}^{\left(B,n\right)}\right|}^{p}\right]\leq C_{p}^{B}E\left[{\left(\int_{t_{k}}^{t_{k+1}}{\left|S_{s-}^{\left(n,M,k\right)}\sigma \left(s,S_{s-}^{\left(n,M,k\right)}\right)\right|}^{2}ds\right)}^{p/2}\right]\\ \; & \leq C_{p}^{B}\delta^{\frac{p}{2}-1}E\left[\int_{t_{k}}^{t_{k+1}}{\left|S_{s-}^{\left(n,M,k\right)}\sigma \left(s,S_{s-}^{\left(n,M,k\right)}\right)\right|}^{p}ds\right]\leq \Gamma_{p}^{\left(B\right)}\delta^{\frac{p}{2}-1}E\left[\int_{t_{k}}^{t_{k+1}}\left(1+|S_{s-}^{\left(n,M,k\right)}{|}^{p}\right)ds\right]\\ \; & =\Gamma_{p}^{\left(B\right)}\delta^{\frac{p}{2}-1}E\left[\int_{t_{k}}^{t_{k+1}}1_{\left(t_{k},t_{k+1}\right]}\left(s\right)\left(1+|S_{s-}^{\left(n,M,k\right)}{|}^{p}\right)ds\right]\leq \Gamma_{p}^{\left(B\right)}\delta^{p/2}\left(1+B_{M,k}^{\left(n\right)}\right). \end{array} \end{array}$$

By the product-space version of the Hölder inequality together with the growth estimate (26), $M^{\left(J,n\right)}$ and $M^{\left(K,n\right)}$ are local martingales. It suffices to consider the case of $M^{\left(J,n\right)}$, since $M^{\left(K,n\right)}$ is handled in the same way. By Lemma 5 (the required shift is covered entirely by Remark 3), the Hölder inequality on $\left(\left[t_{k},t_{k+1}\right]\times R,l⊗\bar{\nu }\right)$, and (26),

$$\begin{array}{cc} E & \left[\underset{t_{k}\leq t\leq t_{k+1}}{\sup}{\left|M_{t}^{\left(J,n\right)}\right|}^{p}\right]=E\left[\underset{t_{k}<t\leq t_{k+1}}{\sup}{\left|M_{t}^{\left(J,n\right)}\right|}^{p}\right]\leq c_{7}E\left[{\left(\int_{t_{k}}^{t_{k+1}}\int_{R}{\left|\tilde{h}\left(s,S_{s-}^{\left(n,M,k\right)},y\right)\right|}^{2}\bar{\nu }\left(dy\right)ds\right)}^{\frac{p}{2}}\right]\\ \; & +c_{8}E\left[\int_{t_{k}}^{t_{k+1}}\int_{R}{\left|\tilde{h}\left(s,S_{s-}^{\left(n,M,k\right)},y\right)\right|}^{p}\bar{\nu }\left(dy\right)ds\right]\\ \; & \leq c_{7}{\left(\delta \bar{\nu }\left(R\right)\right)}^{\frac{p}{2}-1}E\left[\int_{t_{k}}^{t_{k+1}}\int_{R}{\left|\tilde{h}\left(s,S_{s-}^{\left(n,M,k\right)},y,\omega \right)\right|}^{p}\bar{\nu }\left(dy\right)ds\right]\\ \; & +c_{8}C_{p}E\left[\int_{t_{k}}^{t_{k+1}}\left(1+\right|S_{s-}^{\left(n,M,k\right)}{|}^{p})ds\right]\leq C_{p}(c_{7}{\left(\delta \bar{\nu }\left(R\right)\right)}^{\frac{p}{2}-1}+c_{8})E\left[\int_{t_{k}}^{t_{k+1}}\left(1+\right|S_{s-}^{\left(n,M,k\right)}{|}^{p})ds\right]\\ \; & =C_{p}(c_{7}{\left(\delta \bar{\nu }\left(R\right)\right)}^{\frac{p}{2}-1}+c_{8})E\left[\int_{t_{k}}^{t_{k+1}}1_{\left(t_{k},t_{k+1}\right]}\left(s\right)(1+|S_{s-}^{\left(n,M,k\right)}{|}^{p})ds\right]\\ \; & \leq C_{p}(c_{7}{\left(\bar{\nu }\left(R\right)\right)}^{\frac{p}{2}-1}+c_{8})(\delta^{\frac{p}{2}}+\delta )(1+B_{M,k}^{\left(n\right)}). \end{array}$$

Therefore, for $\Gamma_{p}^{\left(J,K\right)}=2^{p}C_{p}\left(c_{7}\bar{\nu }{\left(R\right)}^{\frac{p}{2}-1}+c_{8}\right)$,

(32) $$\begin{array}{c} \begin{array}{cc} E & \left[\underset{t\in [t_{k},t_{k+1}]}{\sup}{\left|M_{t}^{\left(J,n\right)}+M_{t}^{\left(K,n\right)}\right|}^{p}\right]\\ \; & \leq 2^{p-1}\left(E\left[\underset{t_{k}\leq t\leq t_{k+1}}{\sup}{\left|M_{t}^{\left(J,n\right)}\right|}^{p}\right]+E\left[\underset{t_{k}\leq t\leq t_{k+1}}{\sup}{\left|M_{t}^{\left(K,n\right)}\right|}^{p}\right]\right)\leq 2^{p-1}\\ \; & \cdot 2C_{p}\left(c_{7}{\left(\bar{\nu }\left(R\right)\right)}^{\frac{p}{2}-1}+c_{8}\right)\left(\delta^{\frac{p}{2}}+\delta \right)\left(1+B_{M,k}^{\left(n\right)}\right)=\Gamma_{p}^{\left(J,K\right)}\left(\delta^{p/2}+\delta \right)\left(1+B_{M,k}^{\left(n\right)}\right). \end{array} \end{array}$$

Moreover, for each n≥0,

$$\begin{array}{c} \begin{array}{cc} B_{M,k}^{\left(n+1\right)} & =E\left[\underset{t_{k}\leq t\leq t_{k+1}}{\sup}{\left|{\hat{S}}_{t}^{\left(n+1,M,k\right)}\right|}^{p}\right]\leq 4^{p-1}\left(E|\xi_{k}{|}^{p}+E\underset{t_{k}\leq t\leq t_{k+1}}{\sup}{\left|A_{t}^{\left(n\right)}\right|}^{p}\right.\\ \; & \left.+E\underset{t_{k}\leq t\leq t_{k+1}}{\sup}|M_{t}^{\left(B,n\right)}{|}^{p}+E\underset{t_{k}\leq t\leq t_{k+1}}{\sup}{\left|M_{t}^{\left(J,n\right)}+M_{t}^{\left(K,n\right)}\right|}^{p}\right). \end{array} \end{array}$$

Applying (30)–(32), we obtain

$$\begin{array}{c} B_{M,k}^{\left(n+1\right)}\leq 4^{p-1}E{\left|\xi_{k}\right|}^{p}+4^{p-1}\left(M^{p}\delta^{p}+\Gamma_{p}^{\left(B\right)}\delta^{p/2}+\Gamma_{p}^{\left(J,K\right)}\left(\delta^{p/2}+\delta \right)\right)\left(1+B_{M,k}^{\left(n\right)}\right). \end{array}$$

Hence, one can find a constant $C_{0}>0$, independent of n,k,δ, such that

(33) $$B_{M,k}^{\left(n+1\right)}\leq C_{0}E{\left|\xi_{k}\right|}^{p}+C_{0}\left(\delta^{p}+\delta^{p/2}+\delta \right)\left(1+B_{M,k}^{\left(n\right)}\right),\;n\geq 0.$$

By (33), for δ>0 so small that

(34) $$C_{0}\left(\delta^{p}+\delta^{p/2}+\delta \right)\leq \frac{1}{2},$$

there exists a constant $C_{1}>0$ independent of n,k, such that

$$B_{M,k}^{\left(n+1\right)}\leq C_{1}\left(1+E|\xi_{k}{|}^{p}\right)+\frac{1}{2}B_{M,k}^{\left(n\right)}.$$

Let $C^{\prime }=2C_{1}+1>0$. In the following part of the proof, we will assume that δ satisfies (34). One can prove by induction on *n* that

(35) $$\begin{array}{c} \begin{array}{c} B_{M,k}^{\left(n\right)}\leq C^{\prime }\left(1+E|\xi_{k}{|}^{p}\right),\;n\geq 0,\;k\in \left[0:N-1\right]. \end{array} \end{array}$$

In particular, for each n≥0,k∈[0:N−1], ${\hat{S}}^{\left(n,M,k\right)}\in \Lambda^{p}\left(\left[t_{k},t_{k+1}\right]\right)$ and for each n≥1,k∈[0:N−1],

(36) $$\begin{array}{c} \begin{array}{cc} & E\left[\underset{t_{k}\leq t\leq t_{k+1}}{\sup}{\left|D_{t}^{\left(n-1,M,k\right)}\right|}^{p}\right]<\infty , \end{array} \end{array}$$

(37) $$\begin{array}{c} \begin{array}{cc} & S_{s-}^{\left(n,M,k\right)}-S_{s-}^{\left(n-1,M,k\right)}=D_{s-}^{\left(n-1,M,k\right)}\;a.e.,\;Q\text{-}\;a.s., \end{array} \end{array}$$

(38) $$\begin{array}{c} \begin{array}{cc} & \left|D_{s-}^{\left(n-1,M,k\right)}\right|\leq \underset{t_{k}\leq u\leq t_{k+1}}{\sup}\left|D_{u}^{\left(n-1,M,k\right)}\right|,\;s\in \left[t_{k},t_{k+1}\right],\;Q\text{-}\;a.s. \end{array} \end{array}$$

The process $H_{s}^{\left(n\right)}=f\left(S_{s-}^{\left(n\right)},s\right)-f\left(S_{s-}^{\left(n-1\right)},s\right)$ is predictable, since $S^{\left(n\right)}$ and $S^{\left(n-1\right)}$ are adapted càdlàg. Then, by (37) and (38),

(39) $$\begin{array}{c} \begin{array}{cc} \; & {\langle M^{\left(B,D,n\right)}\rangle }_{t_{k+1}}\leq \int_{t_{k}}^{t_{k+1}}|f\left(S_{s-}^{\left(n\right)},s\right)-f\left(S_{s-}^{\left(n-1\right)},s\right){|}^{2}ds\leq L_{\sigma }^{2}\int_{t_{k}}^{t_{k+1}}{\left|S_{s-}^{\left(n\right)}-S_{s-}^{\left(n-1\right)}\right|}^{2}ds\\ \; & =L_{\sigma }^{2}\int_{t_{k}}^{t_{k+1}}|D_{s-}^{\left(n-1\right)}{|}^{2}ds\leq L_{\sigma }^{2}\delta \underset{t_{k}\leq u\leq t_{k+1}}{\sup}{\left|D_{u}^{\left(n-1\right)}\right|}^{2}, \end{array} \end{array}$$

and by (36) and the Hölder inequality, $E\int_{t_{k}}^{t_{k+1}\wedge \rho_{M}}{\left|f\left(S_{s-}^{\left(n\right)},s\right)-f\left(S_{s-}^{\left(n-1\right)},s\right)\right|}^{2}ds$$\leq L_{\sigma }^{2}\delta (E\sup_{t_{k}\leq u\leq t_{k+1}}|D_{u}^{\left(n-1\right)}{{|}^{p})}^{2/p}<\infty$, which implies that $M^{\left(B,D,n\right)}$ is a martingale. By the BDG inequality and (39), for $C_{p}^{DB}=C_{p}^{B}L_{\sigma }^{p}$,

(40) $$\begin{array}{c} \begin{array}{c} E\underset{t_{k}\leq t\leq t_{k+1}}{\sup}{\left|M_{t}^{\left(B,D,n\right)}\right|}^{p}\leq C_{p}^{B}E\left[{\langle M^{\left(B,D,n\right)}\rangle }_{t_{k+1}}^{p/2}\right]\leq C_{p}^{DB}\delta^{p/2}E\underset{t_{k}\leq t\leq t_{k+1}}{\sup}{\left|D_{t}^{\left(n-1\right)}\right|}^{p}. \end{array} \end{array}$$

Similarly, since $S^{\left(n\right)}$ and $S^{\left(n-1\right)}$ are adapted and càdlàg, the processes $H^{\left(J,K,D,n\right)}\left(s,y\right)$ and $G^{\left(J,K,D,n\right)}\left(s,y\right)=H^{\left(J,K,D,n\right)}\left(s,y\right)1_{\left\{s\leq \rho_{M}\right\}}$ are predictable. Moreover, for each y∈R and l⊗Q– a.e.(s,ω)∈T×Ω,

(41) $$|H^{\left(J,K,D,n\right)}\left(s,y\right)|\leq \left(L^{\left(h\right)}\left(y\right)+L^{\left(k\right)}\left(y\right)\right)\left|S_{s-}^{\left(n\right)}-S_{s-}^{\left(n-1\right)}\right|.$$

It is easy to prove, using Hölder’s inequality, that

$$C^{\left(2\right)}=\int_{R}{\left(L^{\left(h\right)}\left(y\right)+L^{\left(k\right)}\left(y\right)\right)}^{2}\bar{\nu }\left(dy\right),\;C^{\left(p\right)}=\int_{R}{\left(L^{\left(h\right)}\left(y\right)+L^{\left(k\right)}\left(y\right)\right)}^{p}\bar{\nu }\left(dy\right)<\infty .$$

Moreover, $M_{t}^{\left(J,K,D,n\right)}=\int_{t_{k}}^{t}\int_{R}G^{\left(J,K,D,n\right)}\left(s,y\right)q\left(ds\;dy\right),\;t\in \left[t_{k},t_{k+1}\right]$. Inequality (41) as well as (37) and (38) imply

(42) $$\begin{array}{c} \begin{array}{cc} & \int_{t_{k}}^{t_{k+1}}\int_{R}{\left|G^{\left(J,K,D,n\right)}\left(s,y\right)\right|}^{2}\bar{\nu }\left(dy\right)ds\leq C^{\left(2\right)}\delta \underset{t_{k}\leq s\leq t_{k+1}}{\sup}{\left|D_{s}^{\left(n-1\right)}\right|}^{2}, \end{array} \end{array}$$

(43) $$\begin{array}{c} \begin{array}{cc} & \int_{t_{k}}^{t_{k+1}}\int_{R}{\left|G^{\left(J,K,D,n\right)}\left(s,y\right)\right|}^{p}\bar{\nu }\left(dy\right)ds\leq C^{\left(p\right)}\delta \underset{t_{k}\leq s\leq t_{k+1}}{\sup}{\left|D_{s}^{\left(n-1\right)}\right|}^{p}. \end{array} \end{array}$$

By inequalities (36) and (42),

$$\begin{array}{cc} E & \left[\int_{t_{k}}^{t_{k+1}}\int_{R}{\left|G^{\left(J,K,D,n\right)}\left(s,y\right)\right|}^{2}\bar{\nu }\left(dy\right)ds\right]\leq C^{\left(2\right)}\delta E\underset{t_{k}\leq s\leq t_{k+1}}{\sup}{\left|D_{s}^{\left(n-1\right)}\right|}^{2}\\ \; & \leq C^{\left(2\right)}\delta \left(1+E\underset{t_{k}\leq s\leq t_{k+1}}{\sup}{\left|D_{s}^{\left(n-1\right)}\right|}^{p}\right)<\infty \end{array}$$

and in particular, $M^{\left(J,K,D,n\right)}$ is a martingale. Similarly, (36), (42), and (43) imply

(44) $$\begin{array}{c} \begin{array}{cc} & E\left[\int_{t_{k}}^{t_{k+1}}\int_{R}{\left|G^{\left(J,K,D,n\right)}\left(s,y\right)\right|}^{p}\bar{\nu }\left(dy\right)ds\right]\leq C^{\left(p\right)}\delta E\underset{t_{k}\leq s\leq t_{k+1}}{\sup}{\left|D_{s}^{\left(n-1\right)}\right|}^{p}, \end{array} \end{array}$$

(45) $$\begin{array}{c} \begin{array}{cc} & E{\left(\int_{t_{k}}^{t_{k+1}}\int_{R}{\left|G^{\left(J,K,D,n\right)}\left(s,y\right)\right|}^{2}\bar{\nu }\left(dy\right)ds\right)}^{p/2}\leq {\left(C^{\left(2\right)}\right)}^{p/2}\delta^{p/2}E\underset{t_{k}\leq s\leq t_{k+1}}{\sup}{\left|D_{s}^{\left(n-1\right)}\right|}^{p}<\infty . \end{array} \end{array}$$

Thus, we can use Lemma 4. The required integrand shift is justified by Remark 2. Its application to $M^{\left(J,K,D,n\right)}$ gives

(46) $$\begin{array}{c} \begin{array}{cc} E & \left[\underset{t_{k}\leq t\leq t_{k+1}}{\sup}{\left|M_{t}^{\left(J,K,D,n\right)}\right|}^{p}\right]=E\left[\underset{t_{k}<t\leq t_{k+1}}{\sup}{\left|M_{t}^{\left(J,K,D,n\right)}\right|}^{p}\right]\\ \; & \leq c_{7}E{\left(\int_{t_{k}}^{t_{k+1}}\int_{R}{\left|G^{\left(J,K,D,n\right)}\left(s,y\right)\right|}^{2}\bar{\nu }\left(dy\right)ds\right)}^{p/2}+c_{8}E\int_{t_{k}}^{t_{k+1}}\int_{R}{\left|G^{\left(J,K,D,n\right)}\left(s,y\right)\right|}^{p}\bar{\nu }\left(dy\right)ds. \end{array} \end{array}$$

Thus, by (44)–(46), we obtain for $C_{p}^{\left(J,K\right)}=c_{7}{\left(C^{\left(2\right)}\right)}^{p/2}+c_{8}C^{\left(p\right)}$,

(47) $$\begin{array}{c} E\underset{t_{k}\leq t\leq t_{k+1}}{\sup}{\left|M_{t}^{\left(J,D,n\right)}+M_{t}^{\left(K,D,n\right)}\right|}^{p}\leq C_{p}^{\left(J,K\right)}\left(\delta^{p/2}+\delta \right)E\underset{t_{k}\leq t\leq t_{k+1}}{\sup}{\left|D_{t}^{\left(n-1\right)}\right|}^{p}. \end{array}$$

We fix n≥1. We will assume that δ<1. By (37) and (38), we have

(48) $$\begin{array}{c} E\underset{t_{k}\leq t\leq t_{k+1}}{\sup}{\left|A_{t}^{\left(D,n\right)}\right|}^{p}\leq M^{p}\delta^{p}E\underset{t_{k}\leq s\leq t_{k+1}}{\sup}{\left|D_{s}^{\left(n-1\right)}\right|}^{p}\leq M^{p}\delta E\underset{t_{k}\leq s\leq t_{k+1}}{\sup}{\left|D_{s}^{\left(n-1\right)}\right|}^{p}. \end{array}$$

By (40), (47), and (48), for $C_{M}^{T}=3^{p-1}\left(M^{p}+C_{p}^{DB}+2C_{p}^{\left(J,K\right)}\right)$,

(49) $$\begin{array}{c} \begin{array}{cc} E\underset{t_{k}\leq t\leq t_{k+1}}{\sup}{\left|D_{t}^{\left(n\right)}\right|}^{p} & \leq 3^{p-1}\left(M^{p}\delta +C_{p}^{DB}\delta^{p/2}+C_{p}^{\left(J,K\right)}\left(\delta^{p/2}+\delta \right)\right)E\underset{t_{k}\leq s\leq t_{k+1}}{\sup}{\left|D_{s}^{\left(n-1\right)}\right|}^{p}\\ \; & \leq C_{M}^{T}\delta E\underset{t_{k}\leq s\leq t_{k+1}}{\sup}{\left|D_{s}^{\left(n-1\right)}\right|}^{p}. \end{array} \end{array}$$

To choose a single time step, we introduce $\delta_{M}=\delta_{M}^{\left(1\right)}\wedge \delta_{M}^{\left(2\right)}$, where $\delta_{M}^{\left(1\right)}>0$ is any number such that (34) holds for all $\delta \leq \delta_{M}^{\left(1\right)}$, and $\delta_{M}^{\left(2\right)}=\frac{1}{2\;C_{M}^{T}}\wedge 1$. Then, for each time partition with mesh $\delta \leq \delta_{M}$, we have $C_{M}^{T}\delta \leq \frac{1}{2}$, and therefore,

(50) $$\begin{array}{c} \begin{array}{c} E\underset{t_{k}\leq t\leq t_{k+1}}{\sup}{\left|D_{t}^{\left(n\right)}\right|}^{p}\leq \frac{1}{2}E\underset{t_{k}\leq t\leq t_{k+1}}{\sup}{\left|D_{t}^{\left(n-1\right)}\right|}^{p}. \end{array} \end{array}$$

We now turn to the construction of the limit on the interval $\left[t_{k},t_{k+1}\right]$.

Henceforth, we assume the stronger condition $\delta \leq \delta_{M}$. Let us fix k∈[0:N−1]. Clearly, $\xi_{k}\in L^{p}\left(\Omega \right)$. Then, for each n≥0, we have the uniform bound $B_{M,k}^{\left(n\right)}\leq C_{M,p,k}^{p}$, where $C_{M,p,k}={\left(C^{\prime }\left(1+E|\xi_{k}{|}^{p}\right)\right)}^{1/p}<\infty$ depends only on M,p, and δ. In particular,

(51) $$\begin{array}{c} \begin{array}{cc} & \underset{n\in N}{\sup}{∥{\hat{S}}^{\left(n,M,k\right)}∥}_{\Lambda^{p}\left(\left[t_{k},t_{k+1}\right]\right)}\leq C_{M,p,k}<\infty , \end{array} \end{array}$$

(52) $$\begin{array}{c} \begin{array}{cc} & {∥{\hat{S}}^{\left(1,M,k\right)}-{\hat{S}}^{\left(0,M,k\right)}∥}_{\Lambda^{p}\left(\left[t_{k},t_{k+1}\right]\right)}={\left(E\underset{t\in [t_{k},t_{k+1}]}{\sup}{\left|{\hat{S}}_{t}^{\left(1,M,k\right)}-{\hat{S}}_{t}^{\left(0,M,k\right)}\right|}^{p}\right)}^{1/p}\\ \; & \;\;\;\;\;\;\;\;\;\;\;\;\;\;\;\;\;\;\;\;\;\;\;\;\;\;\;\;\;\;\;\;\;\;\;\;\;\;\;\;\;\;\;\;\;\;\;\leq {\left(2^{p-1}\left(B_{M,k}^{\left(1\right)}+B_{M,k}^{\left(0\right)}\right)\right)}^{1/p}<\infty . \end{array} \end{array}$$

Iterating (50), we obtain

(53) $$\begin{array}{c} \begin{array}{c} {∥{\hat{S}}^{\left(n+1,M,k\right)}-{\hat{S}}^{\left(n,M,k\right)}∥}_{\Lambda^{p}\left(\left[t_{k},t_{k+1}\right]\right)}\leq 2^{-n/p}{∥{\hat{S}}^{\left(1,M,k\right)}-{\hat{S}}^{\left(0,M,k\right)}∥}_{\Lambda^{p}\left(\left[t_{k},t_{k+1}\right]\right)}. \end{array} \end{array}$$

Therefore, (52) and (53) imply that the sequence ${\left({\hat{S}}^{\left(n,M,k\right)}\right)}_{n\geq 0}$ is Cauchy in the complete space $\Lambda^{p}\left(\left[t_{k},t_{k+1}\right]\right)$, since for each m>n≥0,

$$\begin{array}{cc} & {∥{\hat{S}}^{\left(m,M,k\right)}-{\hat{S}}^{\left(n,M,k\right)}∥}_{\Lambda^{p}\left(\left[t_{k},t_{k+1}\right]\right)}\leq \sum_{j=n}^{m-1}{∥{\hat{S}}^{\left(j+1,M,k\right)}-{\hat{S}}^{\left(j,M,k\right)}∥}_{\Lambda^{p}\left(\left[t_{k},t_{k+1}\right]\right)}\\ \; & \leq \sum_{j=n}^{\infty }{∥{\hat{S}}^{\left(j+1,M,k\right)}-{\hat{S}}^{\left(j,M,k\right)}∥}_{\Lambda^{p}\left(\left[t_{k},t_{k+1}\right]\right)}\leq {∥{\hat{S}}^{\left(1,M,k\right)}-{\hat{S}}^{\left(0,M,k\right)}∥}_{\Lambda^{p}\left(\left[t_{k},t_{k+1}\right]\right)}\sum_{j=n}^{\infty }2^{-j/p}\;\underset{n,m\to \infty }{\to }\;0. \end{array}$$

The contraction estimate implies that the Picard sequence is Cauchy in $\Lambda^{p}\left(\left[t_{k},t_{k+1}\right]\right)$.

Therefore, there exists a càdlàg adapted process ${\hat{S}}^{\left(M,k\right)}:\left[t_{k},t_{k+1}\right]\times \Omega \to R$ such that ${\hat{S}}^{\left(n,M,k\right)}⟶{\hat{S}}^{\left(M,k\right)}\;\mathrm{in}\;\Lambda^{p}\left(\left[t_{k},t_{k+1}\right]\right)$ and clearly, ${∥{\hat{S}}^{\left(M,k\right)}∥}_{\Lambda^{p}\left(\left[t_{k},t_{k+1}\right]\right)}\leq C_{M,p,k}$. It enables to define a process $S^{\left(M,k\right)}$ on $\left[0,t_{k+1}\right]$ by

$$S^{\left(M,k\right)}\left(t,\omega \right)=Y_{k}\left(t,\omega \right)\;\mathrm{for}\;t\in \left[0,t_{k}\right],\;\omega \in \Omega ,\;S^{\left(M,k\right)}\left(t\right)={\hat{S}}^{\left(M,k\right)}\left(t\right)\;\mathrm{for}\;t\in \left(t_{k},t_{k+1}\right].$$

By construction of the Picard iterates, $Y_{k}\left(t_{k},\omega \right)={\hat{S}}^{\left(M,k\right)}\left(t_{k},\omega \right),\;\omega \in \Omega ,$ and therefore, $S^{\left(M,k\right)}$ is a càdlàg adapted process on $\left[0,t_{k+1}\right]$. In turn, the processes $Y_{k}$, k≥0, used in our Picard construction, are defined as follows. We set $Y_{0}\left(0,\omega \right)=\xi_{0}\left(\omega \right)$ and $Y_{1}\left(t,\omega \right)$$=S_{t}^{\left(M,0\right)}\left(\omega \right),\;t\in \left[0,t_{1}\right],\;\omega \in \Omega$. If for some k≥1, $Y_{k}\in \Lambda^{p}\left(\left[0,t_{k}\right]\right)$ is already constructed, then we assume that $\xi_{k}\left(\omega \right)=Y_{k}\left(t_{k},\omega \right),\;\omega \in \Omega$, $Y_{k+1}\left(t,\omega \right)=Y_{k}\left(t,\omega \right)\;\mathrm{for}\;t\in \left[0,t_{k}\right]$, $\omega \in \Omega ,\;Y_{k+1}\left(t,\omega \right)=S_{t}^{\left(M,k\right)}\left(\omega \right)\;\mathrm{for}\;t\in \left(t_{k},t_{k+1}\right],\;\omega \in \Omega .$ Then, $Y_{k+1}$ is adapted and càdlàg, and since $E[\sup_{t\in [0,t_{k+1}]}|Y_{k+1}{\left(t\right)|}^{p}]\leq E[\sup_{t\in [0,t_{k}]}|Y_{k}{\left(t\right)|}^{p}]+E[\sup_{t\in [t_{k},t_{k+1}]}{|S_{t}^{\left(M,k\right)}|}^{p}]<\infty$, $Y_{k+1}\in \Lambda^{p}\left(\left[0,t_{k+1}\right]\right)$.

Next, we verify uniqueness on the interval $\left[t_{k},t_{k+1}\right]$.

We will show uniqueness of the process $S^{\left(M,k\right)}$ on $\left[t_{k},t_{k+1}\right]$. Let ${\tilde{S}}^{\left(M,k\right)}$ be another process on $\left[t_{k},t_{k+1}\right]$ obtained the same way as $S^{\left(M,k\right)}$ with the same initial condition $\xi_{k}$ at time $t_{k}$. Proceeding similarly to (36)–(49), with $D_{t}^{\left(n-1\right)}$ replaced by $\Delta_{t}=S_{t}^{\left(M,k\right)}$$-{\tilde{S}}_{t}^{\left(M,k\right)},\;t\in \left[t_{k},t_{k+1}\right]$, we have

(54) $$\begin{array}{c} \begin{array}{c} E\underset{t_{k}\leq t\leq t_{k+1}}{\sup}{\left|\Delta_{t}\right|}^{p}\leq C_{M}^{T}\delta E\underset{t_{k}\leq s\leq t_{k+1}}{\sup}{\left|\Delta_{s}\right|}^{p}\leq \frac{1}{2}E\underset{t_{k}\leq t\leq t_{k+1}}{\sup}{\left|\Delta_{t}\right|}^{p}, \end{array} \end{array}$$

which implies that $\Delta_{t}=0$ a.s. on $\left[t_{k},t_{k+1}\right]$, and therefore $S^{\left(M,k\right)}$ and ${\tilde{S}}^{\left(M,k\right)}$ are indistinguishable on $\left[t_{k},t_{k+1}\right]$. We use the fact that $\Delta_{t_{k}}=\Delta_{t_{k}-}=0$: for k=0 this follows from the standard càdlàg convention at $t_{0}=0$, while for k>0 it is satisfied outside a null set after the previously proved indistinguishability on $\left[0,t_{k}\right]$, since by construction, $S^{\left(M,k\right)}=S^{\left(M,k-1\right)}$ and ${\tilde{S}}^{\left(M,k\right)}={\tilde{S}}^{\left(M,k-1\right)}$, so their values and left limits at $t_{k}$ are equal.

The same argument as in the proof for (54) shows that for $t\in [t_{k},t_{k+1}]$,

(55) $$\begin{array}{c} \begin{array}{cc} & {∥\int_{t_{k}}^{t\wedge \rho_{M}}\left(X\left(S_{s-}^{\left(n,M,k\right)},ds\right)-X\left(S_{s-}^{\left(M,k\right)},ds\right)\right)∥}_{\Lambda^{p}\left(\left[t_{k},t_{k+1}\right]\right)}^{p}\leq C_{M}^{T}\delta {∥S^{\left(n,M,k\right)}-S^{\left(M,k\right)}∥}_{\Lambda^{p}\left(\left[t_{k},t_{k+1}\right]\right)}^{p}\\ \;\;\; & \leq \frac{1}{2}{∥S^{\left(n,M,k\right)}{{|}_{\left[t_{k},t_{k+1}\right]}-S^{\left(M,k\right)}|}_{\left[t_{k},t_{k+1}\right]}∥}_{\Lambda^{p}\left(\left[t_{k},t_{k+1}\right]\right)}^{p}=\frac{1}{2}{∥{\hat{S}}^{\left(n,M,k\right)}-{\hat{S}}^{\left(M,k\right)}∥}_{\Lambda^{p}\left(\left[t_{k},t_{k+1}\right]\right)}^{p}\underset{n\to \infty }{\to }0. \end{array} \end{array}$$

Since

$${\hat{S}}_{t\wedge \rho_{M}}^{\left(n+1,M,k\right)}=S_{t_{k}}^{\left(M,k\right)}+\int_{t_{k}}^{t\wedge \rho_{M}}X\left({\hat{S}}_{s-}^{\left(n,M,k\right)},ds\right),\;t\in \left[t_{k},t_{k+1}\right],$$

we obtain

$${\hat{S}}_{t\wedge \rho_{M}}^{\left(n+1,M,k\right)}=S_{t_{k}}^{\left(M,k\right)}+\int_{t_{k}}^{t\wedge \rho_{M}}X\left({\hat{S}}_{s-}^{\left(M,k\right)},ds\right)+\int_{t_{k}}^{t\wedge \rho_{M}}\left(X\left(S_{s-}^{\left(n,M,k\right)},ds\right)-X\left(S_{s-}^{\left(M,k\right)},ds\right)\right).$$

Since ${\hat{S}}^{\left(n+1,M,k\right)}\to {\hat{S}}^{\left(M,k\right)}\;\mathrm{in}\;\Lambda^{p}\left(\left[t_{k},t_{k+1}\right]\right)$ and (55) holds, we get

(56) $$\begin{array}{c} \begin{array}{c} {\hat{S}}_{t\wedge \rho_{M}}^{\left(M,k\right)}=S_{t_{k}}^{\left(M,k\right)}+\int_{t_{k}}^{t\wedge \rho_{M}}X\left({\hat{S}}_{s-}^{\left(M,k\right)},ds\right),\;t\in \left[t_{k},t_{k+1}\right]. \end{array} \end{array}$$

Applying (56) on the successive subintervals $\left[t_{k},t_{k+1}\right]$, we obtain a process

(57) $$\begin{array}{c} S^{\left(M\right)}=S^{\left(M,N-1\right)}:T\times \Omega \to R\in \Lambda^{p}, \end{array}$$

which solves (25) on T with the integral taken up to $t\wedge \rho_{M}$. It is easy to verify that if $M^{\prime }<M$, then on $\left[0,\rho_{M^{\prime }}\right]$ also $|r_{s-}|\leq M^{\prime }$, and thus, $S_{t}^{\left(M^{\prime }\right)}=S_{t}^{\left(M\right)},\;t\leq \rho_{M^{\prime }}$, which implies the consistency of the sequence ${\left(S^{\left(M\right)}\right)}_{M\in N}$. Moreover, by the inequality $E[\sup_{t\leq T}|r_{t}|]<\infty$, we have the convergence $\rho_{M}↑T$ a.s. Therefore, a process *S* on T given by

(58) $$S_{t}=S_{t}^{\left(M\right)}\;\mathrm{for}\;\mathrm{any}\;M\;\mathrm{for}\;\mathrm{which}\;t\leq \rho_{M},$$

is well defined. Since for each M∈N, the process $S^{\left(M\right)}$ satisfies (13) on $\left[0,\rho_{M}\right]$ and (58) holds, for all $t\leq \rho_{M}$,

(59) $$\begin{array}{c} \begin{array}{cc} S_{t} & =S_{0}+\int_{0}^{t}S_{s-}r_{s-}ds+\int_{0}^{t}S_{s-}\sigma \left(s,S_{s-}\right)dW_{s}^{\left(2\right)}\\ \; & +\int_{0}^{t}\int_{R}\tilde{h}\left(s,S_{s-},y\right)q\left(ds\;dy\right)+\int_{0}^{t}\int_{R}\tilde{k}\left(s,S_{s-},y\right)q\left(ds\;dy\right). \end{array} \end{array}$$

Since $\rho_{M}↑T$ a.s., for each fixed t∈T we have

$$P\left(\overset{\infty }{\underset{M=1}{⋃}}\left\{t\leq \rho_{M}\right\}\right)=1.$$

On the event $\left\{t\leq \rho_{M}\right\}$ the identity (59) holds, and by consistency, this defines $S_{t}$. As a consequence, *S* satisfies (13) on T.

Finally, we combine the interval-wise uniqueness with consistency to obtain global uniqueness.

Finally, if *S* and $\tilde{S}$ are two solutions on T starting from the same initial condition $S_{0}$, then for each M∈N, $S_{t}^{\left(M\right)}=S_{t\wedge \rho_{M}},\;{\tilde{S}}_{t}^{\left(M\right)}={\tilde{S}}_{t\wedge \rho_{M}},\;t\in T$, satisfy the same localised equation on each interval $\left[t_{k},t_{k+1}\right]$ with the same initial condition at $t_{k}$. Therefore, as it was shown above, they are indistinguishable on each interval. Moreover, by the consistency across intervals, we have $S_{t}^{\left(M\right)}={\tilde{S}}_{t}^{\left(M\right)},\;t\in T$ a.s. As a consequence, since $\rho_{M}↑T$ a.s., the solution is unique, i.e., $S_{t}={\tilde{S}}_{t},\;t\in T$ a.s. □

**Theorem** **2.** *Let Assumption 2 hold, and let r satisfy the tail condition *(21)*. Then, SDE *(13)* admits a unique adapted càdlàg solution $S\in \Lambda^{p}$.*

**Proof.**  The existence of a unique adapted càdlàg solution to (13) follows from the previous lemma. The process $S^{\left(M\right)}$ given by (57) satisfies on T the semimartingale decomposition

(60) $$\begin{array}{c} \begin{array}{cc} S_{t}^{\left(M\right)} & =S_{0}+\int_{0}^{t\wedge \rho_{M}}S_{s-}^{\left(M\right)}r_{s-}ds+\int_{0}^{t\wedge \rho_{M}}S_{s-}^{\left(M\right)}\sigma \left(s,S_{s-}^{\left(M\right)}\right)dW_{s}^{\left(2\right)}\\ \; & +\int_{0}^{t\wedge \rho_{M}}\int_{R}\tilde{h}\left(s,S_{s-}^{\left(M\right)},y\right)q\left(ds\;dy\right)+\int_{0}^{t\wedge \rho_{M}}\int_{R}\tilde{k}\left(s,S_{s-}^{\left(M\right)},y\right)q\left(ds\;dy\right). \end{array} \end{array}$$

Applying the Itô formula (see [29] (Theorem 4.4.7)), we obtain

(61) $$\begin{array}{c} \begin{array}{cc} {\left|S_{t}^{\left(M\right)}\right|}^{p} & ={\left|S_{0}\right|}^{p}+\int_{0}^{t\wedge \rho_{M}}p{\left|S_{s-}^{\left(M\right)}\right|}^{p-2}S_{s-}^{\left(M\right)}S_{s-}^{\left(M\right)}r_{s-}ds\\ \; & +\int_{0}^{t\wedge \rho_{M}}p{\left|S_{s-}^{\left(M\right)}\right|}^{p-2}S_{s-}^{\left(M\right)}S_{s-}^{\left(M\right)}\sigma \left(s,S_{s-}^{\left(M\right)}\right)dW_{s}^{\left(2\right)}\\ \; & +\int_{0}^{t\wedge \rho_{M}}\int_{R}\left({\left|S_{s-}^{\left(M\right)}+\tilde{h}\left(s,S_{s-}^{\left(M\right)},y\right)\right|}^{p}-{\left|S_{s-}^{\left(M\right)}\right|}^{p}\right)q\left(ds\;dy\right)\\ \; & +\int_{0}^{t\wedge \rho_{M}}\int_{R}\left({\left|S_{s-}^{\left(M\right)}+\tilde{k}\left(s,S_{s-}^{\left(M\right)},y\right)\right|}^{p}-{\left|S_{s-}^{\left(M\right)}\right|}^{p}\right)q\left(ds\;dy\right)+\int_{0}^{t\wedge \rho_{M}}H_{M}\left(s\right)ds, \end{array} \end{array}$$

$$H_{M}\left(s\right)=H_{M}^{\left(c\right)}\left(s\right)+H_{M}^{\left(h\right)}\left(s\right)+H_{M}^{\left(k\right)}\left(s\right),$$

(62) $$\begin{array}{c} \begin{array}{cc} H_{M}^{\left(c\right)}\left(s\right) & =\frac{1}{2}p\left(p-1\right){\left|S_{s-}^{\left(M\right)}\right|}^{p-2}{\left(S_{s-}^{\left(M\right)}\sigma \left(s,S_{s-}^{\left(M\right)}\right)\right)}^{2}, \end{array} \end{array}$$

(63) $$\begin{array}{c} \begin{array}{cc} H_{M}^{\left(h\right)}\left(s\right) & =\int_{R}\left({\left|S_{s-}^{\left(M\right)}+\tilde{h}\left(s,S_{s-}^{\left(M\right)},y\right)\right|}^{p}-{\left|S_{s-}^{\left(M\right)}\right|}^{p}-p{\left|S_{s-}^{\left(M\right)}\right|}^{p-2}S_{s-}^{\left(M\right)}\tilde{h}\left(s,S_{s-}^{\left(M\right)},y\right)\right)\bar{\nu }\left(dy\right), \end{array} \end{array}$$

(64) $$\begin{array}{c} \begin{array}{cc} H_{M}^{\left(k\right)}\left(s\right) & =\int_{R}\left({\left|S_{s-}^{\left(M\right)}+\tilde{k}\left(s,S_{s-}^{\left(M\right)},y\right)\right|}^{p}-{\left|S_{s-}^{\left(M\right)}\right|}^{p}-p{\left|S_{s-}^{\left(M\right)}\right|}^{p-2}S_{s-}^{\left(M\right)}\tilde{k}\left(s,S_{s-}^{\left(M\right)},y\right)\right)\bar{\nu }\left(dy\right). \end{array} \end{array}$$

The Taylor formula with reminder for $\varphi \left(x\right)={\left|x\right|}^{p}$ implies

(65) $${\left|x+z\right|}^{p}-{\left|x\right|}^{p}-p{\left|x\right|}^{p-2}xz\leq C_{p}^{\varphi }\left({\left|x\right|}^{p-2}{\left|z\right|}^{2}+{\left|z\right|}^{p}\right),\;x,z\in R,$$

where $C_{p}^{\varphi }>0$ is a constant depending only on *p*. The application of (65) gives

(66) $$\begin{array}{c} H_{M}^{\left(h\right)}\left(s\right)\leq C_{p}^{\varphi }\int_{R}\left({\left|S_{s-}^{\left(M\right)}\right|}^{p-2}{\left|\tilde{h}\left(s,S_{s-}^{\left(M\right)},y\right)\right|}^{2}+{\left|\tilde{h}\left(s,S_{s-}^{\left(M\right)},y\right)\right|}^{p}\right)1_{\left\{s\leq \rho_{M}\right\}}\bar{\nu }\left(dy\right). \end{array}$$

We fix (s,ω) for which (26) holds and set $x=S_{s-}^{\left(M\right)}\left(\omega \right)$. By (66), Lemma 6, and (26), $H_{M}^{\left(h\right)}\left(s,\omega \right)\leq {\hat{C}}_{p}^{\varphi }\left(1+\right|S_{s-}^{\left(M\right)}\left(\omega \right){|}^{p})$ for ${\hat{C}}_{p}^{\varphi }=C_{p}^{\varphi }\left({\hat{C}}_{p}+C_{p}\right)$ and (l⊗Q)-a.e. (s,ω). Similarly, $H_{M}^{\left(k\right)}\left(s,\omega \right)\leq {\hat{C}}_{p}^{\varphi }\left(1+\right|S_{s-}^{\left(M\right)}\left(\omega \right){|}^{p})$ for (l⊗Q)-a.e. (s,ω). Finally, for (s,ω) satisfying (26) and $x=S_{s-}^{\left(M\right)}\left(\omega \right)$, $H_{M}^{\left(c\right)}\left(s\right)\leq \frac{1}{2}p\left(p-1\right)(|S_{s-}^{\left(M\right)}\sigma \left(s,S_{s-}^{\left(M\right)}\right){|}^{p}+|S_{s-}^{\left(M\right)}{|}^{p})\leq C_{p}^{\varphi ,c}\left(1+\right|S_{s-}^{\left(M\right)}{|}^{p})$, where the constant $C_{p}^{\varphi ,c}>0$ is independent of *M*. Thus, $H_{M}^{\left(c\right)}\left(s\right)\leq C_{p}^{\varphi ,c}\left(1+\right|S_{s-}^{\left(M\right)}{|}^{p})$ holds (l⊗Q)- a.e. and therefore, for a constant $C_{p}^{\left(H\right)}>0$ independent of *M* and l⊗Q- almost all (s,ω)∈T×Ω,

(67) $$H_{M}\left(s\right)=H_{M}^{\left(c\right)}\left(s\right)+H_{M}^{\left(h\right)}\left(s\right)+H_{M}^{\left(k\right)}\left(s\right)\leq C_{p}^{\left(H\right)}\left(1+{\left|S_{s-}^{\left(M\right)}\right|}^{p}\right).$$

Let $f_{M}\left(t\right)=E|S_{t}^{\left(M\right)}{|}^{p}=E{\left|S_{t\wedge \rho_{M}}^{\left(M\right)}\right|}^{p}$. From (61), it follows that

$$\begin{array}{c} \begin{array}{cc} f_{M}\left(t\right) & =E{\left|S_{0}\right|}^{p}+E\int_{0}^{t\wedge \rho_{M}}p{\left|S_{s-}^{\left(M\right)}\right|}^{p-2}S_{s-}^{\left(M\right)}S_{s-}^{\left(M\right)}r_{s-}ds+E\int_{0}^{t\wedge \rho_{M}}H_{M}\left(s\right)ds\\ \; & +E\int_{0}^{t\wedge \rho_{M}}p{\left|S_{s-}^{\left(M\right)}\right|}^{p-2}S_{s-}^{\left(M\right)}S_{s-}^{\left(M\right)}\sigma \left(s,S_{s-}^{\left(M\right)}\right)dW_{s}^{\left(2\right)}\\ \; & +E\int_{0}^{t\wedge \rho_{M}}\int_{R}\left({\left|S_{s-}^{\left(M\right)}+\tilde{h}\left(s,S_{s-}^{\left(M\right)},y\right)\right|}^{p}-{\left|S_{s-}^{\left(M\right)}\right|}^{p}\right)q\left(ds\;dy\right)\\ \; & +E\int_{0}^{t\wedge \rho_{M}}\int_{R}\left({\left|S_{s-}^{\left(M\right)}+\tilde{k}\left(s,S_{s-}^{\left(M\right)},y\right)\right|}^{p}-{\left|S_{s-}^{\left(M\right)}\right|}^{p}\right)q\left(ds\;dy\right). \end{array} \end{array}$$

We will show that the three last expected values are equal to 0. For each n∈N, we define the stopping time $\tau_{n}=\inf\{t\geq 0:|S_{t}^{\left(M\right)}\left|\geq n\right\}\wedge T$. Applying the Itô formula to $\varphi \left(x\right)={\left|x\right|}^{p}$ and $S_{t\wedge \tau_{n}}^{\left(M\right)}$, we get the following counterpart of (61).

(68) $$\begin{array}{c} \begin{array}{cc} {\left|S_{t\wedge \tau_{n}}^{\left(M\right)}\right|}^{p} & ={\left|S_{0}\right|}^{p}+\int_{0}^{t\wedge \rho_{M}\wedge \tau_{n}}p{\left|S_{s-}^{\left(M\right)}\right|}^{p-2}S_{s-}^{\left(M\right)}S_{s-}^{\left(M\right)}r_{s-}ds\\ \; & +\int_{0}^{t\wedge \rho_{M}\wedge \tau_{n}}p{\left|S_{s-}^{\left(M\right)}\right|}^{p-2}S_{s-}^{\left(M\right)}S_{s-}^{\left(M\right)}\sigma \left(s,S_{s-}^{\left(M\right)}\right)dW_{s}^{\left(2\right)}\\ \; & +\int_{0}^{t\wedge \rho_{M}\wedge \tau_{n}}\int_{R}\left({\left|S_{s-}^{\left(M\right)}+\tilde{h}\left(s,S_{s-}^{\left(M\right)},y\right)\right|}^{p}-{\left|S_{s-}^{\left(M\right)}\right|}^{p}\right)q\left(ds\;dy\right)\\ \; & +\int_{0}^{t\wedge \rho_{M}\wedge \tau_{n}}\int_{R}\left({\left|S_{s-}^{\left(M\right)}+\tilde{k}\left(s,S_{s-}^{\left(M\right)},y\right)\right|}^{p}-{\left|S_{s-}^{\left(M\right)}\right|}^{p}\right)q\left(ds\;dy\right)\\ \; & +\int_{0}^{t\wedge \rho_{M}\wedge \tau_{n}}H_{M}\left(s\right)ds,\;t\in T. \end{array} \end{array}$$

All integrands in the first to the fourth integral are predictable and on $\left[0,\tau_{n}\right]$ and $\left\{s\leq \rho_{M}\right\}$,

$$\begin{array}{c} \begin{array}{cc} E & \int_{0}^{T}{\left|p{\left|S_{s-}^{\left(M\right)}\right|}^{p-2}S_{s-}^{\left(M\right)}S_{s-}^{\left(M\right)}\sigma \left(s,S_{s-}^{\left(M\right)}\right)\right|}^{2}1_{\left\{s\leq \rho_{M}\right\}}1_{\left\{s\leq \tau_{n}\right\}}ds\leq Tp^{2}n^{2p-2}C_{\sigma }^{2/p}{\left(1+n^{p}\right)}^{2/p}<\infty . \end{array} \end{array}$$

Thus, the Brownian integral is a martingale with expectation zero.

By Lemma 7 with $x=S_{s-}^{\left(M\right)}$ and $u=\tilde{h}\left(s,S_{s-}^{\left(M\right)},y\right)$ and the Hölder inequality, we get

$$\begin{array}{cc} E & \int_{0}^{T}\int_{R}\left|\left({\left|S_{s-}^{\left(M\right)}+\tilde{h}\left(s,S_{s-}^{\left(M\right)},y\right)\right|}^{p}-{\left|S_{s-}^{\left(M\right)}\right|}^{p}\right)1_{\left\{s\leq \rho_{M}\right\}}1_{\left\{s\leq \tau_{n}\right\}}\right|\bar{\nu }\left(dy\right)ds\\ \; & \leq E\int_{0}^{T}\int_{R}\left|{\overset{ˇ}{C}}_{p}\left({\left|S_{s-}^{\left(M\right)}\right|}^{p-1}\left|\tilde{h}\left(s,S_{s-}^{\left(M\right)},y\right)\right|+{\left|\tilde{h}\left(s,S_{s-}^{\left(M\right)},y\right)\right|}^{p}\right)1_{\left\{s\leq \rho_{M}\right\}}1_{\left\{s\leq \tau_{n}\right\}}\right|\bar{\nu }\left(dy\right)ds\\ \; & \leq {\overset{ˇ}{C}}_{p}n^{p-1}\left(1+n\right)T\int_{R}\left|K^{\left(h\right)}\left(y\right)\right|\bar{\nu }\left(dy\right)+{\overset{ˇ}{C}}_{p}{\left(1+n\right)}^{p}T\int_{R}{\left|K^{\left(h\right)}\left(y\right)\right|}^{p}\bar{\nu }\left(dy\right)\\ \; & \leq {\overset{ˇ}{C}}_{p}n^{p-1}\left(1+n\right)T{\left(\int_{R}{\left|K^{\left(h\right)}\left(y\right)\right|}^{p}\bar{\nu }\left(dy\right)\right)}^{1/p}{\left(\bar{\nu }\left(R\right)\right)}^{(p-1)/p}\\ \; & +{\overset{ˇ}{C}}_{p}{\left(1+n\right)}^{p}T\int_{R}{\left|K^{\left(h\right)}\left(y\right)\right|}^{p}\bar{\nu }\left(dy\right)<\infty . \end{array}$$

Similarly,

$$E\int_{0}^{T}\int_{R}\left|\left({\left|S_{s-}^{\left(M\right)}+\tilde{k}\left(s,S_{s-}^{\left(M\right)},y\right)\right|}^{p}-{\left|S_{s-}^{\left(M\right)}\right|}^{p}\right)1_{\left\{s\leq \rho_{M}\right\}}1_{\left\{s\leq \tau_{n}\right\}}\right|\bar{\nu }\left(dy\right)ds<\infty$$

and therefore, by [30] (Remark in Subsection 2.2), the third and the fourth integrals in (68) are martingales with expectation zero.

Let $f_{M}^{\left(n\right)}\left(t\right)=E{\left|S_{t\wedge \rho_{M}\wedge \tau_{n}}^{\left(M\right)}\right|}^{p},\;t\in T$. From (67) and the equality $S_{s\wedge \rho_{M}\wedge \tau_{n}}^{\left(M\right)}=S_{s\wedge \rho_{M}}^{\left(M\right)}$ on the set $\left\{s\leq \tau_{n}\right\}$, it follows that $\left|H_{M}\left(s\right)\right|1_{\left\{s\leq \tau_{n}\right\}}\leq C_{p}^{\left(H\right)}\left(1+\right|S_{s\wedge \rho_{M}\wedge \tau_{n}}^{\left(M\right)}{|}^{p})$. Using (68) and the above-established vanishing of the expectations of the mentioned integrals, we obtain

$$\begin{array}{c} \begin{array}{cc} f_{M}^{\left(n\right)}\left(t\right) & \leq E|S_{0}{|}^{p}+pM\int_{0}^{t}E{\left|S_{s\wedge \rho_{M}\wedge \tau_{n}}^{\left(M\right)}\right|}^{p}ds+C_{p}^{\left(H\right)}\int_{0}^{t}\left(1+E{\left|S_{s\wedge \rho_{M}\wedge \tau_{n}}^{\left(M\right)}\right|}^{p}\right)ds\\ \; & =S_{0}^{p}+\left(pM+C_{p}^{\left(H\right)}\right)\int_{0}^{t}f_{M}^{\left(n\right)}\left(s\right)ds+C_{p}^{\left(H\right)}t\leq C+b\int_{0}^{t}f_{M}^{\left(n\right)}\left(s\right)ds,\;t\in T, \end{array} \end{array}$$

with $C=S_{0}^{p}+C_{p}^{\left(H\right)}T$ and $b=pM+C_{p}^{\left(H\right)}$. By Proposition 1 applied to *C*, $\alpha \left(t\right)=f_{M}^{\left(n\right)}\left(t\right)$ and β(s)=b, we obtain $f_{M}^{\left(n\right)}\left(t\right)\leq C_{p}\left(M,T\right)=Ce^{bT},\;t\in T$, and $C_{p}\left(M,T\right)$ is independent of *n*.

Since $t\wedge \rho_{M}\wedge \tau_{n}↑t\wedge \rho_{M}$ and $S^{\left(M\right)}$ is càdlàg, we have

$$S_{t\wedge \rho_{M}\wedge \tau_{n}}^{\left(M\right)}⟶S_{t\wedge \rho_{M}}^{\left(M\right)}\;a.s.$$

Hence, by Fatou’s lemma,

$$E{\left|S_{t\wedge \rho_{M}}^{\left(M\right)}\right|}^{p}\leq \underset{n\to \infty }{\mathrm{lim inf}}E{\left|S_{t\wedge \rho_{M}\wedge \tau_{n}}^{\left(M\right)}\right|}^{p}\leq C_{p}\left(M,T\right),\;t\in T.$$

Therefore,

(69) $$\begin{array}{c} f_{M}\left(t\right)=E{\left|S_{t\wedge \rho_{M}}^{\left(M\right)}\right|}^{p}\leq \left(S_{0}^{p}+C_{p}^{\left(H\right)}T\right)e^{\left(pM+C_{p}^{\left(H\right)}\right)T},\;t\in T, \end{array}$$

(70) $$\begin{array}{c} \underset{0\leq t\leq T}{\sup}E{\left|S_{t\wedge \rho_{M}}^{\left(M\right)}\right|}^{p}\leq K_{p}e^{\kappa_{p}M},\;M\in N, \end{array}$$

where $K_{p}=\left(S_{0}^{p}+C_{p}^{\left(H\right)}T\right)e^{C_{p}^{\left(H\right)}T}>0,\;\kappa_{p}=pT>0$.

By construction, $S_{t}=S_{t}^{\left(M\right)}$ on $\left\{\sup_{0\leq s\leq T}|r_{s}|\leq M\right\}$. To find a uniform estimate for $E[\sup_{t\leq T}|S_{t}{|}^{p}]$, we introduce the stopping times and cadlag, adapted processes

$$\tau_{N}=\inf\{t\geq 0:|S_{t-}^{\left(M\right)}\left|\geq N\right\}\wedge T,\;N\in N,\;Y_{t}^{\left(M,N\right)}=S_{t\wedge \tau_{N}\wedge \rho_{M}}^{\left(M\right)},\;t\in T.$$

By (70) and (60),

(71) $$\begin{array}{c} \underset{0\leq t\leq T}{\sup}E{\left|Y_{t}^{\left(M,N\right)}\right|}^{p}\leq \underset{0\leq t\leq T}{\sup}E{\left|S_{t\wedge \rho_{M}}^{\left(M\right)}\right|}^{p}\leq K_{p}e^{\kappa_{p}M}<\infty , \end{array}$$

$$\begin{array}{cc} \; & Y_{t}^{\left(M,N\right)}=Y_{t}^{\left(M,N\right)}=S_{0}+A_{t}^{\left(M,N\right)}+M_{t}^{\left(M,N\right)},\;A_{t}^{\left(M,N\right)}=\int_{0}^{t\wedge \tau_{N}\wedge \rho_{M}}S_{s-}^{\left(M\right)}r_{s-}ds,\\ \; & M_{t}^{\left(M,N\right)}=M_{t}^{\left(B,M,N\right)}+M_{t}^{\left(J,M,N\right)},\;M_{t}^{\left(B,M,N\right)}=\int_{0}^{t\wedge \tau_{N}\wedge \rho_{M}}S_{s-}^{\left(M\right)}\sigma \left(s,S_{s-}^{\left(M\right)}\right)dW_{s},\\ \; & M_{t}^{\left(J,M,N\right)}=\int_{0}^{t\wedge \tau_{N}\wedge \rho_{M}}\int_{R}\tilde{h}\left(s,S_{s-}^{\left(M\right)},y\right)q\left(ds\;dy\right)+\int_{0}^{t\wedge \tau_{N}\wedge \rho_{M}}\int_{R}\tilde{k}\left(s,S_{s-}^{\left(M\right)},y\right)q\left(ds\;dy\right). \end{array}$$

We estimate the components of the above decomposition on $\left[0,\tau_{N}\wedge \rho_{M}\right]$. $S^{\left(M\right)}$ is cadlag, and therefore, $|S_{s-}^{\left(M\right)}\left|=\right|S_{s}^{\left(M\right)}|$ for a.e. *s*, which implies by the Jensen inequality,

(72) $$\begin{array}{c} \begin{array}{cc} \underset{u\leq t}{\sup}{\left|A_{u}^{\left(M,N\right)}\right|}^{p} & \leq M^{p}{\left(\int_{0}^{t\wedge \tau_{N}\wedge \rho_{M}}\left|S_{s-}^{\left(M\right)}\right|ds\right)}^{p}=M^{p}{\left(\int_{0}^{t}\left|Y_{s}^{\left(M,N\right)}\right|ds\right)}^{p}\\ \; & \leq M^{p}t^{p-1}\int_{0}^{t}{\left|Y_{s}^{\left(M,N\right)}\right|}^{p}ds\leq M^{p}T^{p-1}\int_{0}^{T}{\left|Y_{s}^{\left(M,N\right)}\right|}^{p}ds,\;t\in T. \end{array} \end{array}$$

Taking expectations and using (71), we obtain for $K_{p}^{\left(A\right)}=K_{p}T^{p}$

(73) $$\begin{array}{c} \begin{array}{cc} E\left[\underset{u\leq T}{\sup}{\left|A_{u}^{\left(M,N\right)}\right|}^{p}\right] & \leq M^{p}T^{p-1}\int_{0}^{T}E{\left|Y_{s}^{\left(M,N\right)}\right|}^{p}ds\leq M^{p}T^{p-1}T\underset{0\leq s\leq T}{\sup}E{\left|Y_{s}^{\left(M,N\right)}\right|}^{p}\\ \; & \leq K_{p}^{\left(A\right)}M^{p}e^{\kappa_{p}M}. \end{array} \end{array}$$

The integrand of $M^{\left(B,M,N\right)}$ is predictable and by (26),

$$\begin{array}{cc} E & \int_{0}^{T}{\left|S_{s-}^{\left(M\right)}\sigma \left(s,S_{s-}^{\left(M\right)}\right)\right|}^{2}ds\leq E\int_{0}^{T}\left(1+C_{p}\left(1+{\left|S_{s-}^{\left(M\right)}\right|}^{p}\right)\right)ds\\ \; & \leq E\int_{0}^{T}\left(\left(C_{p}+1\right)(1+{\left|S_{s-}^{\left(M\right)}\right|}^{p}\right)ds\leq T\left(C_{p}+1\right)\left(1+N^{p}\right)ds<\infty , \end{array}$$

which implies that $M^{\left(B,M,N\right)}$ is a martingale. By the BDG inequality, the Jensen inequality on T, and (26), for $K_{p}^{\left(B\right)}=C_{p}^{B}C_{p}T^{\frac{p}{2}-1}$,

$$\begin{array}{cc} E & \left[\underset{u\leq t}{\sup}{\left|M_{u}^{\left(B,M,N\right)}\right|}^{p}\right]\leq C_{p}^{B}E\left[{\left(\int_{0}^{t\wedge \tau_{N}\wedge \rho_{M}}{\left|S_{s-}^{\left(M\right)}\sigma \left(s,S_{s-}^{\left(M\right)}\right)\right|}^{2}ds\right)}^{p/2}\right]\\ \; & \leq C_{p}^{B}T^{\frac{p}{2}-1}\int_{0}^{t\wedge \tau_{N}\wedge \rho_{M}}E\left[{\left|S_{s-}^{\left(M\right)}\sigma \left(s,S_{s-}^{\left(M\right)}\right)\right|}^{p}\right]ds\leq K_{p}^{\left(B\right)}\int_{0}^{t\wedge \tau_{N}\wedge \rho_{M}}\left(1+E{\left|S_{s-}^{\left(M\right)}\right|}^{p}\right)ds. \end{array}$$

The càdlàg property of sample paths of $S^{\left(M\right)}$ implies the equality $S_{s-}^{\left(M\right)}=S_{s}^{\left(M\right)}$ for Lebesgue-a.e. s∈T. Moreover, on $\left\{s\leq \rho_{M}\right\}$ we have $S_{s}^{\left(M\right)}=S_{s\wedge \rho_{M}}^{\left(M\right)}$. Therefore, for Lebesgue-a.e.s∈T, $E|S_{s-}^{\left(M\right)}{|}^{p}=E{\left|S_{s\wedge \rho_{M}}^{\left(M\right)}\right|}^{p}$. Thus,

(74) $$\begin{array}{c} E\left[\underset{u\leq t}{\sup}{\left|M_{u}^{\left(B,M,N\right)}\right|}^{p}\right]\leq K_{p}^{\left(B\right)}\int_{0}^{t}\left(1+E{\left|S_{s\wedge \rho_{M}}^{\left(M\right)}\right|}^{p}\right)ds. \end{array}$$

By (26), for $C_{p}^{\left(2\right)}=2^{p-1}C_{p}$ and (l⊗Q)-a.e. (s,ω),

(75) $$\int_{R}{\left|\tilde{g}\left(s,S_{s-}^{\left(M\right)},y\right)\right|}^{p}\bar{\nu }\left(dy\right)\leq C_{p}^{\left(2\right)}\left(1+{\left|S_{s-}^{\left(M\right)}\right|}^{p}\right).$$

Using the Jensen inequality and (75), for $C_{p}^{\left(3\right)}=\bar{\nu }{\left(R\right)}^{\frac{p}{2}-1}C_{p}^{\left(2\right)}$ and (l⊗Q)-a.e. (s,ω) yields

(76) $$\begin{array}{c} \begin{array}{cc} {\left(\int_{R}{\left|\tilde{g}\left(s,S_{s-}^{\left(M\right)},y\right)\right|}^{2}\bar{\nu }\left(dy\right)\right)}^{p/2}\; & \leq \bar{\nu }{\left(R\right)}^{\frac{p}{2}-1}\int_{R}{\left|\tilde{g}\left(s,S_{s-}^{\left(M\right)},y\right)\right|}^{p}\bar{\nu }\left(dy\right)\leq C_{p}^{\left(3\right)}\left(1+{\left|S_{s-}^{\left(M\right)}\right|}^{p}\right). \end{array} \end{array}$$

From (71) and (75), it follows that $M^{\left(J,M,N\right)}$ is a martingale. Indeed,

(77) $$\begin{array}{c} \begin{array}{cc} E & \left[\int_{0}^{T}\int_{R}{\left|\tilde{g}\left(s,S_{s-}^{\left(M\right)},y\right)\right|}^{2}\bar{\nu }\left(dy\right)ds\right]\leq E\left[\int_{0}^{T}\int_{R}\left(1+{\left|\tilde{g}\left(s,S_{s-}^{\left(M\right)},y\right)\right|}^{p}\right)\bar{\nu }\left(dy\right)ds\right]\leq T\bar{\nu }\left(R\right)\\ \; & +C_{p}^{\left(2\right)}\int_{0}^{T}\left(1+E{\left|S_{s\wedge \tau_{N}\wedge \rho_{M}}^{\left(M\right)}\right|}^{p}\right)ds=T\left(\bar{\nu }\left(R\right)+C_{p}^{\left(2\right)}\right)+C_{p}^{\left(2\right)}\int_{0}^{T}E{\left|S_{s\wedge \tau_{N}\wedge \rho_{M}}^{\left(M\right)}\right|}^{p}ds<\infty . \end{array} \end{array}$$

Applying the Jensen inequality, (76), and the Fubini theorem, we get for $C_{p}^{\left(4\right)}=T^{\frac{p}{2}-1}C_{p}^{\left(3\right)}$,

(78) $$\begin{array}{c} E\left[{\left(\int_{0}^{t\wedge \tau_{N}\wedge \rho_{M}}\int_{R}{\left|\tilde{g}\left(s,S_{s-}^{\left(M\right)},y\right)\right|}^{2}\bar{\nu }\left(dy\right)ds\right)}^{p/2}\right]\leq C_{p}^{\left(4\right)}\int_{0}^{t\wedge \tau_{N}\wedge \rho_{M}}\left(1+E{\left|S_{s-}^{\left(M\right)}\right|}^{p}\right)ds. \end{array}$$

In turn, by (75) and Fubini’s theorem,

(79) $$\begin{array}{c} \begin{array}{cc} E & \left[\int_{0}^{t\wedge \tau_{N}\wedge \rho_{M}}\int_{R}{\left|\tilde{g}\left(s,S_{s-}^{\left(M\right)},y\right)\right|}^{p}\bar{\nu }\left(dy\right)ds\right]\\ \; & =\int_{0}^{t\wedge \tau_{N}\wedge \rho_{M}}E\left[\int_{R}{\left|\tilde{g}\left(s,S_{s-}^{\left(M\right)},y\right)\right|}^{p}\bar{\nu }\left(dy\right)\right]ds\leq C_{p}^{\left(2\right)}\int_{0}^{t\wedge \tau_{N}\wedge \rho_{M}}\left(1+E{\left|S_{s-}^{\left(M\right)}\right|}^{p}\right)ds. \end{array} \end{array}$$

By (78), (79), Lemma 4 (cf. Remark 2), we obtain for $K_{p}^{\left(J\right)}=\left(c_{7}\vee c_{8}\right)\left(C_{p}^{\left(2\right)}+C_{p}^{\left(4\right)}\right)$,

(80) $$\begin{array}{c} E\left[\underset{u\leq t}{\sup}{\left|M_{u}^{\left(J,M,N\right)}\right|}^{p}\right]=E\left[\underset{0<u\leq t}{\sup}{\left|M_{u}^{\left(J,M,N\right)}\right|}^{p}\right]\leq K_{p}^{\left(J\right)}\int_{0}^{t\wedge \tau_{N}\wedge \rho_{M}}\left(1+E{\left|S_{s-}^{\left(M\right)}\right|}^{p}\right)ds. \end{array}$$

Inequalities (70), (74), and (80) imply for $K_{p}^{\left(M\right)}=2^{p-1}\left(K_{p}^{\left(B\right)}+K_{p}^{\left(J\right)}\right)$,

(81) $$\begin{array}{c} \begin{array}{c} E\left[\underset{u\leq t}{\sup}{\left|M_{u}^{\left(M,N\right)}\right|}^{p}\right]\leq K_{p}^{\left(M\right)}\int_{0}^{t}\left(1+E{\left|S_{s\wedge \rho_{M}}^{\left(M\right)}\right|}^{p}\right)ds\leq K_{p}^{\left(M\right)}t\left(1+K_{p}e^{\kappa_{p}M}\right),\;t\in T. \end{array} \end{array}$$

By (73) and (81), we obtain for $K_{p}^{\left(S\right)}=3^{p-1}\left(S_{0}^{p}+K_{p}^{\left(M\right)}T+K_{p}^{\left(M\right)}TK_{p}+K_{p}^{\left(A\right)}\right)$,

$$\begin{array}{cc} E & \left[\underset{u\leq t}{\sup}{\left|Y_{u}^{\left(M,N\right)}\right|}^{p}\right]\leq 3^{p-1}S_{0}^{p}+3^{p-1}E\left[\underset{u\leq t}{\sup}{\left|A_{u}^{\left(M,N\right)}\right|}^{p}\right]+3^{p-1}E\left[\underset{u\leq t}{\sup}{\left|M_{u}^{\left(M,N\right)}\right|}^{p}\right]\\ \; & \leq 3^{p-1}S_{0}^{p}+3^{p-1}K_{p}^{\left(A\right)}M^{p}e^{\kappa_{p}M}+3^{p-1}K_{p}^{\left(M\right)}T\left(1+K_{p}e^{\kappa_{p}M}\right)\leq K_{p}^{\left(S\right)}M^{p}e^{\kappa_{p}M},\;t\in T, \end{array}$$

since M≥1 and $e^{\kappa_{p}M}\geq 1$. Moreover,

$$\underset{t\leq T}{\sup}{\left|S_{t\wedge \tau_{N}\wedge \rho_{M}}^{\left(M\right)}\right|}^{p}↑\underset{t\leq T}{\sup}{\left|S_{t\wedge \rho_{M}}^{\left(M\right)}\right|}^{p}\;a.s.\;\mathrm{as}\;N\to \infty$$

and therefore, by the monotone convergence theorem,

$$E\left[\underset{t\leq T}{\sup}{\left|S_{t}^{\left(M\right)}\right|}^{p}\right]=E\left[\underset{t\leq T}{\sup}{\left|S_{t\wedge \rho_{M}}^{\left(M\right)}\right|}^{p}\right]=\lim_{N\to \infty }E\left[\underset{u\leq t}{\sup}{\left|Y_{u}^{\left(M,N\right)}\right|}^{p}\right]\leq K_{p}^{\left(S\right)}M^{p}e^{\kappa_{p}M},\;M\in N.$$

Thus, we obtained the moment estimate of order *p*, using the solution constructed in the previous lemma together with inequality (26) and the first inequality in Lemma 6 formulated for the exponent *p*. The assumptions of this theorem include, in addition, growth and Lipschitz conditions for the exponent 2p. These stronger assumptions imply the corresponding conditions for *p*, and they allow us to apply (27) and the second inequality in Lemma 6 formulated for the exponent 2p. Using the stronger growth and Lipschitz conditions for the exponent 2p, we repeat the moment estimate with *p* replaced by 2p. This yields

(82) $$\begin{array}{c} E\left[\underset{t\leq T}{\sup}{\left|S_{t}^{\left(M\right)}\right|}^{2p}\right]\leq K_{2p}^{\left(S\right)}M^{2p}e^{\kappa_{2p}M},\;M\in N, \end{array}$$

where constants $K_{2p}^{\left(S\right)}>0$ and $\kappa_{2p}>0$ are independent of *M* and depend only on the coefficients of the SDE and the exponent 2p.

We define the following disjoint partition of Ω: $A_{n}=\{n-1\leq \sup_{0\leq t\leq T}|r_{t}\left|<n\right\},\;n\geq 1$. On $A_{n}$ we have $\rho_{n}=T$. Therefore, $S_{t}=S_{t}^{\left(n\right)},\;t\in T$, and

$$E\left[\underset{t\leq T}{\sup}{\left|S_{t}\right|}^{p}\right]=\sum_{n=1}^{\infty }E\left[\underset{t\leq T}{\sup}{\left|S_{t}^{\left(n\right)}\right|}^{p}1_{A_{n}}\right].$$

Since $Q\left(A_{n}\right)\leq C_{r}\exp\left(-c_{r}{\left(n-2\right)}^{\beta }\right)$, n≥2, and by (82),

$${\left(E\left[\underset{t\leq T}{\sup}{\left|S_{t}^{\left(n\right)}\right|}^{2p}\right]\right)}^{1/2}\leq {\left(K_{2p}^{\left(S\right)}\right)}^{1/2}\exp\left(\frac{1}{2}\kappa_{2p}n+p\log n\right),$$

the Hölder inequality implies that

(83) $$\begin{array}{c} \begin{array}{cc} E\left[\underset{t\leq T}{\sup}{\left|S_{t}\right|}^{p}\right] & \leq \sum_{n=1}^{\infty }{\left(E\left[\underset{t\leq T}{\sup}{\left|S_{t}^{\left(n\right)}\right|}^{2p}\right]\right)}^{1/2}Q{\left(A_{n}\right)}^{1/2}\leq {\left(K_{2p}^{\left(S\right)}\right)}^{1/2}\exp\left(\frac{1}{2}\kappa_{2p}\right)\cdot 1\\ \; & +{\left(K_{2p}^{\left(S\right)}\right)}^{1/2}C_{r}^{1/2}\sum_{n=2}^{\infty }\exp\left(p\log n-\frac{1}{2}c_{r}{\left(n-2\right)}^{\beta }+\frac{1}{2}\kappa_{2p}n\right)<\infty . \end{array} \end{array}$$

Therefore, $S\in \Lambda^{p}$. Finally, by (83), $\sup_{t\leq T}E|S_{t}{|}^{p}\leq E[\sup_{s\leq T}|S_{s}{|}^{p}]<\infty .$ □

**Remark** **4.** *Since r is an adapted càdlàg process, $r_{t-}=r_{t}$ for Lebesgue- a.e. t∈T and therefore, in the drift term of* (13)*, we can equivalently write $S_{s-}r_{s-}$ or $S_{s-}r_{s}$. The choice $r_{s-}$ is convenient because it makes the drift coefficient predictable, while all integrability assumptions are formulated for $r_{s}$.*

The following example shows that the weaker assumptions on *r* are not sufficient to guarantee that the solution *S* belongs to $\Lambda^{p}$.

**Example** **1.** *Let σ≡0,h≡0,k≡0, so that *(13)* reduces to*

(84) $$dS_{t}=S_{t}r_{t}dt,\;S_{0}>0.$$

*Let q≥1 and $r_{t}=R,\;t\in T$, where R is a Pareto-distributed random variable with scale 1 and shape α>q. Then, r is adapted, càdlàg, $\sup_{0\leq t\leq T}{\left|r_{t}\right|}^{q}=R^{q}$, and since α>q, $E\left[\sup_{t\leq T}{\left|r_{t}\right|}^{q}\right]<\infty$. Therefore, the weak assumption *(22)* for r with q and Assumption 2 (then also Assumption 1) hold. Simultaneously, for x≥1, $Q(\sup_{0\leq t\leq T}|r_{t}\left|>x\right)=Q\left(R>x\right)=x^{-\alpha }$. Thus, *(21)* is not satisfied since $x^{-\alpha }$ decays only polynomially, and therefore, it cannot be bounded by $C_{r}e^{-c_{r}x^{\beta }}$ for all large x. Clearly, the unique pathwise solution to *(84)* has the form $S_{t}=S_{0}e^{Rt},\;t\in T$, and $\sup_{0\leq t\leq T}{\left|S_{t}\right|}^{p}=S_{0}^{p}e^{pTR}$. By integration by substitution,*

(85) $$\begin{array}{c} \begin{array}{cc} E\underset{0\leq t\leq T}{\sup}{\left|S_{t}\right|}^{p} & =S_{0}^{p}Ee^{pTR}=S_{0}^{p}\int_{0}^{\infty }Q\left(e^{pTR}>x\right)dx=S_{0}^{p}\int_{0}^{\infty }Q\left(R>\frac{lnx}{pT}\right)dx\\ \; & =S_{0}^{p}\int_{-\infty }^{\infty }pTe^{pTz}Q\left(R>z\right)dz=S_{0}^{p}\left(\int_{-\infty }^{1}pTe^{pTz}dz+\int_{1}^{\infty }pTe^{pTz}z^{-\alpha }dz\right)\\ \; & =S_{0}^{p}\left(e^{pT}+\int_{1}^{\infty }pTe^{pTz}z^{-\alpha }dz\right)=\infty \end{array} \end{array}$$

*and therefore, $S\notin \Lambda^{p}$. Thus, the considered case shows that Assumption 2 and the weak condition on r do not ensure $S\in \Lambda^{p}$.*

## 4. The Dupire Formula for an Underlying Asset Model with a Generalised Jump Structure and Stochastic Interest Rate

This section is devoted to the derivation and proof of the Dupire formula in the case where the underlying asset price *S* is described by (13) and the short rate *r* is an adapted and càdlàg process satisfying tail condition (21). Such an approach enables considering a generalised structure of jumps of *S* and a stochastic dynamics of the short rate. We will apply Theorem 1, presented in Section 2, Theorem 2, which provides the existence and uniqueness of the regular solution to the previously mentioned SDE, and the theory of pricing contingent claims. We use a distributional formulation of the Dupire equation because the payoff of a European call option is not classically differentiable with respect to the strike variable, and the distributional level of regularity provides a natural framework for a rigorous derivation of the formula.

As indicated above, the process $r={\left(r_{t}\right)}_{t\in T}$, which models the value of the risk-free instantaneous interest rate, is adapted with *l*-integrable sample paths on T a.s. The *savings account*, denoted by $B={\left(B_{t}\right)}_{t\in T}$, is an adapted process of finite variation and has continuous sample paths given by

$$B_{t}=\exp\left(\int_{0}^{t}r_{u}du\right),\;t\in T.$$

The short-rate process *r* and the discount process satisfy the assumptions stated in the previous section. Moreover, we introduce the symbols B(s,t),0≤s≤t≤T, where B(s,t) denotes the price at time *s* of a zero-coupon bond of maturity *t*. We make the assumption that there exists the spot martingale measure $P^{*}$ on $\left(\Omega ,F_{T}\right)$ for B(s,t) (see, e.g., [23] for further details). Therefore, there are no arbitrage opportunities between bonds and the savings account. Furthermore, one has the following equality (see [23] (Eqn. 9.14))

(86) $$B\left(s,t\right)=E^{*}\left(\exp\left(-\int_{s}^{t}r_{u}du\right)|F_{s}\right),\;0\leq s\leq t\leq T,$$

where the symbol $E^{*}$ denotes the expected value with respect to $P^{*}$.

Additionally, for t∈T, we consider the forward martingale measure for the settlement date *t*, denoted by $P_{t}$, on $\left(\Omega ,F_{t}\right)$, which is equivalent to $P^{*}$ and is defined by the Radon–Nikodým derivative of the form (see [23] (Def. 9.6.2))

(87) $$\frac{dP_{t}}{dP^{*}}=\frac{1}{B\left(0,t\right)B_{t}},\;P^{*}-a.s.$$

and we will use the symbol $E^{P_{t}}$ to denote the expected value with respect to $P_{t}$.

In this section, the SDE (13) introduced in the preceding section is considered, used here with the predictable processes *h*, *k*, $\tilde{h}$, and $\tilde{k}$ denoted by $h_{2}$, $k_{2}$, ${\tilde{h}}_{2}$, and ${\tilde{k}}_{2}$. All references to the existence and uniqueness of its solution, as well as to Assumption 2, concern Equation (13) under this change of notation. Moreover, throughout this section, the processes and measures X, $W^{\left(2\right)}$, N, *q*, and $\bar{\nu }$ are those defined in Section 2. This allows the theory developed earlier, including Theorem 1, to be applied directly.

We assume that $Q=P^{*}$. The following lemma shows that under Assumption 2 with p≥4 and condition (21) on the short rate, the discounted asset price S/B is a square-integrable Q-martingale, which ensures the absence of arbitrage in the market. For the proof we refer the reader to Appendix A.

**Lemma** **9.** *Let Assumption 2 be satisfied for p≥4 and let condition *(21)* hold. Then, $S_{t}/B_{t},\;t\in T,$ is a square-integrable Q-martingale.*

We will use the following lemma. Its proof can be found in Appendix A.

**Lemma** **10.** *Let S be the solution to *(13)* under the probability measure Q, with Assumption 2 holding for p=4 and condition *(21)* satisfied. Then $E\int_{0}^{T}H\left(t\right)dt<\infty$, where*

$$\begin{array}{cc} H(t) & =r_{t-}^{2}R_{t}^{2}+S_{t-}^{2}\sigma^{2}\left(t,S_{t-}\right)+{\left(S_{t-}r_{t-}-\int_{R}{\tilde{k}}_{2}\left(t,S_{t-},y\right)\bar{\nu }\left(dy\right)\right)}^{2}\\ \; & +\int_{R}\left({\tilde{h}}_{2}^{4}\left(t,S_{t-},y\right)+{\tilde{k}}_{2}^{4}\left(t,S_{t-},y\right)\right)\bar{\nu }\left(dy\right)+S_{t-}^{2}+R_{t}^{2}\left(1+S_{t-}^{2}+S_{t-}^{2}\sigma^{2}\left(t,S_{t-}\right)\right.\\ \; & \left.+\int_{R}\left({\tilde{h}}_{2}^{2}\left(t,S_{t-},y\right)+{\tilde{k}}_{2}^{2}\left(t,S_{t-},y\right)\right)\bar{\nu }\left(dy\right)\right). \end{array}$$

We now state the main theorem concerning the Dupire formula in the considered case.

**Theorem** **3.** *Let the price S of the underlying asset be described by *(13)* under the probability measure Q, where Assumption 2 with p=4 holds and condition *(21)* is satisfied for the short rate r. Then, the Dupire formula for C has the following form in $D^{\prime }$:*

(88) $$\begin{array}{c} \begin{array}{cc} \frac{\partial C}{\partial t}= & \frac{\sigma^{2}\left(t,k\right)k^{2}}{2}\frac{\partial^{2}C}{\partial k^{2}}+kB\left(0,t\right)E^{P_{t}}\left(r_{t}1_{S_{t-}>k}\right)\\ \; & +\int_{R}\int_{R}E^{P_{t}}\left(\left.j\left(S_{t-},{\tilde{h}}_{2}\left(t,S_{t-},y\right)\right)\left(k\right)+j\left(S_{t-},{\tilde{k}}_{2}\left(t,S_{t-},y\right)\right)\left(k\right)\right|S_{t-}=x\right)\Phi \left(t,dx\right)\bar{\nu }\left(dy\right), \end{array} \end{array}$$

*where σ is called the local volatility.*

**Proof.**  By Theorem 2, the SDE (13) has a unique adapted càdlàg solution $S\in \Lambda^{4}\left(T\right)$. Moreover, by Lemma 1, for every q≥1, ${\left(R_{t}\right)}_{t\in T}\in \Lambda^{q}$. From the assumptions, it follows that

$$\begin{array}{cc} E\left[\int_{0}^{T}{\left|S_{t-}r_{t-}\right|}^{2}dt\right]\; & \leq TE\left[\underset{s\leq T}{\sup}|S_{s}{|}^{2}\cdot \underset{s\leq T}{\sup}{\left|r_{s}\right|}^{2}\right]\\ \; & \leq T{\left(E\left[\underset{s\leq T}{\sup}{\left|S_{s}\right|}^{4}\right]\right)}^{1/2}{\left(E\left[\underset{s\leq T}{\sup}{\left|r_{s}\right|}^{4}\right]\right)}^{1/2}<\infty \end{array}$$

by the Hölder inequality,

$$\begin{array}{cc} E\left[\int_{0}^{T}{\left|S_{t-}\sigma \left(t,S_{t-}\right)\right|}^{2}dt\right] & \leq E\left[\int_{0}^{T}\left(1+|S_{t-}\sigma \left(t,S_{t-}\right){|}^{4}\right)dt\right]\\ \; & \leq T+C_{\sigma }E\left[\int_{0}^{T}\left(1+|S_{t-}{|}^{4}\right)dt\right]<\infty , \end{array}$$

$$\begin{array}{cc} E\left[\int_{0}^{T}\int_{R}{\left|h_{2}\left(t,S_{t-},y\right)\right|}^{2}\bar{\nu }\left(dy\right)dt\right]\; & \leq E\left[\int_{0}^{T}\left(1+\right|S_{t-}{\left|\right)}^{2}dt\right]\cdot \int_{R}{\left(K^{\left(h\right)}\right)}^{2}\left(y\right)\bar{\nu }\left(dy\right)<\infty , \end{array}$$

and

$$\begin{array}{cc} E\left[\int_{0}^{T}\int_{R}{\left|k_{2}\left(t,S_{t-},y\right)\right|}^{2}\bar{\nu }\left(dy\right)dt\right]\; & \leq E\left[\int_{0}^{T}\left(1+\right|S_{t-}{\left|\right)}^{2}dt\right]\cdot \int_{R}{\left(K^{\left(k\right)}\right)}^{2}\left(y\right)\bar{\nu }\left(dy\right)<\infty . \end{array}$$

The integrands are also predictable. Therefore,

$$S_{t-}r_{t-},S_{t-}\sigma \left(t,S_{t-}\right)\in M\left(T\right),\;h_{2}\left(t,S_{t-},y\right)1_{\left\{|y|<1\right\}}\left(y\right),k_{2}\left(t,S_{t-},y\right)1_{\left\{|y|\geq 1\right\}}\left(y\right)\in M_{loc}^{2}\left(\hat{P},\bar{\nu },R\right).$$

Application of Theorem 1 requires identifying the processes and other objects from Section 2.3 with their counterparts in the current formulation.

We have $\alpha ={\hat{h}}_{1}={\hat{k}}_{1}=\phi^{\left(1\right)}=0$, N≡1, and for t∈T, ${\hat{h}}_{2}\left(t,y\right)=h_{2}\left(t,S_{t-},y\right)$, ${\hat{k}}_{2}\left(t,y\right)=k_{2}\left(t,S_{t-},y\right)$, ${\tilde{\hat{h}}}_{2}\left(t,y\right)={\tilde{h}}_{2}\left(t,S_{t-},y\right)$, ${\tilde{\hat{k}}}_{2}\left(t,y\right)={\tilde{k}}_{2}\left(t,S_{t-},y\right)$, ${\tilde{N}}_{t}=R_{t}$, $\beta_{t}=-r_{t}R_{t}$,

$$\begin{array}{cc} \; & \sigma_{t}=\bar{\sigma }\left(t,S_{t-}\right)=S_{t-}\sigma \left(t,S_{t-}\right),\;\gamma_{t}=r_{t-}S_{t-}-\int_{R}{\tilde{k}}_{2}\left(t,S_{t-},y\right)\bar{\nu }\left(dy\right),\;\phi_{t}^{\left(2\right)}=R_{t}\sigma_{t}=R_{t}\bar{\sigma }\left(t,S_{t-}\right),\\ \; & \lambda_{t}=-R_{t}\int_{R}{\tilde{k}}_{2}\left(t,S_{t-},y\right)\bar{\nu }\left(dy\right),\;{\tilde{\xi }}^{\left(2\right)}\left(t,y\right)=R_{t}{\tilde{k}}_{2}\left(t,S_{t-},y\right),\;Q^{t}=P^{t}. \end{array}$$

Moreover, if t∈T, then

$$\begin{array}{cc} \; & E^{t}\left(\sigma_{t}^{2}|S_{t-}=k\right)=E^{Q}\left(\sigma_{t}^{2}|S_{t-}=k\right)=E^{Q}\left({\bar{\sigma }}^{2}\left(t,S_{t-}\right)|S_{t-}=k\right)={\bar{\sigma }}^{2}\left(t,k\right)=\sigma^{2}\left(t,k\right)k^{2},\\ \; & E^{t}\left(\left.\frac{\lambda_{t}}{{\tilde{N}}_{t-}}-\frac{\beta_{t}}{{\tilde{N}}_{t-}}k\right|S_{t-}=x\right)=E^{t}\left(\left.-\int_{R}{\tilde{k}}_{2}\left(t,S_{t-},y\right)\bar{\nu }\left(dy\right)+r_{t}k\right|S_{t-}=x\right)\\ \; & =-E^{t}\left(\left.\int_{R}{\tilde{k}}_{2}\left(t,S_{t-},y\right)\bar{\nu }\left(dy\right)\right|S_{t-}=x\right)+kE^{t}\left(\left.r_{t}\right|S_{t-}=x\right),\\ \; & E^{t}\left(\left.\frac{{\tilde{\xi }}^{\left(2\right)}\left(t,y\right)}{{\tilde{N}}_{t-}}-\frac{{\tilde{k}}_{1}\left(t,S_{t-},y\right)}{{\tilde{N}}_{t-}}k\right|S_{t-}=x\right)=E^{t}\left(\left.{\tilde{k}}_{2}\left(t,S_{t-},y\right)\right|S_{t-}=x\right),\\ \; & E^{t}\left(\frac{{\tilde{h}}_{1}\left(t,S_{t-},y\right)j\left(S_{t-},{\tilde{h}}_{2}\left(t,S_{t-},y\right)\right)\left(k\right)+{\tilde{k}}_{1}\left(t,S_{t-},y\right)j\left(S_{t-},{\tilde{k}}_{2}\left(t,S_{t-},y\right)\right)\left(k\right)}{{\tilde{N}}_{t-}}\right.\\ \; & \left.\left.+j\left(S_{t-},{\tilde{h}}_{2}\left(t,S_{t-},y\right)\right)\left(k\right)+j\left(S_{t-},{\tilde{k}}_{2}\left(t,S_{t-},y\right)\right)\left(k\right)\right|S_{t-}=x\right)\\ \; & =E^{t}\left(\left.j\left(S_{t-},{\tilde{h}}_{2}\left(t,S_{t-},y\right)\right)\left(k\right)+j\left(S_{t-},{\tilde{k}}_{2}\left(t,S_{t-},y\right)\right)\left(k\right)\right|S_{t-}=x\right),\\ \; & \Phi \left(t,dx\right)=E^{Q}{\tilde{N}}_{t-}\mu_{S_{t-}}^{t}\left(dx\right), \end{array}$$

$$\begin{array}{cc} \; & \int_{k}^{\infty }\Phi \left(t,dx\right)=E^{Q}{\tilde{N}}_{t-}E^{t}1_{S_{t-}>k}=-\frac{\partial C\left(t,k\right)}{\partial k},\\ \; & \int_{k}^{\infty }E^{t}\left(\left.\frac{\lambda_{t}}{{\tilde{N}}_{t-}}-\frac{\beta_{t}}{{\tilde{N}}_{t-}}k\right|S_{t-}=x\right)\Phi \left(t,dx\right)=-\int_{k}^{\infty }E^{t}\left(\left.\int_{R}{\tilde{k}}_{2}\left(t,S_{t-},y\right)\bar{\nu }\left(dy\right)\right|S_{t-}=x\right)\Phi \left(t,dx\right)\\ \; & +k\int_{R}1_{x>k}E^{t}\left(\left.r_{t}\right|S_{t-}=x\right)\Phi \left(t,dx\right)=-\int_{k}^{\infty }E^{t}\left(\left.\int_{R}{\tilde{k}}_{2}\left(t,S_{t-},y\right)\bar{\nu }\left(dy\right)\right|S_{t-}=x\right)\Phi \left(t,dx\right)\\ \; & +k\int_{R}E^{t}\left(\left.r_{t}1_{S_{t-}>k}\right|S_{t-}=x\right)\Phi \left(t,dx\right)\\ & =-E^{Q}{\tilde{N}}_{t-}\int_{k}^{\infty }E^{t}\left(\left.\int_{R}{\tilde{k}}_{2}\left(t,S_{t-},y\right)\bar{\nu }\left(dy\right)\right|S_{t-}=x\right)\mu_{S_{t-}}^{t}\left(dx\right)+kE^{Q}{\tilde{N}}_{t-}E^{t}\left(r_{t}1_{S_{t-}>k}\right)\\ & =-B\left(0,t\right)E^{t}\left(\int_{R}{\tilde{k}}_{2}\left(t,S_{t-},y\right)\bar{\nu }\left(dy\right)1_{S_{t-}>k}\right)+kB\left(0,t\right)E^{P^{t}}\left(r_{t}1_{S_{t-}>k}\right),\\ \; & \int_{R}\int_{k}^{\infty }E^{t}\left(\left.\frac{{\tilde{\xi }}^{\left(2\right)}\left(t,y\right)}{{\tilde{N}}_{t-}}-\frac{{\tilde{k}}_{1}\left(t,S_{t-},y\right)}{{\tilde{N}}_{t-}}k\right|S_{t-}=x\right)\Phi \left(t,dx\right)\bar{\nu }\left(dy\right)\\ \; & =\int_{R}\int_{k}^{\infty }E^{t}\left(\left.{\tilde{k}}_{2}\left(t,S_{t-},y\right)\right|S_{t-}=x\right)\Phi \left(t,dx\right)\bar{\nu }\left(dy\right)=B\left(0,t\right)E^{t}\left(\int_{R}{\tilde{k}}_{2}\left(t,S_{t-},y\right)\bar{\nu }\left(dy\right)1_{S_{t-}>k}\right). \end{array}$$

We also have

$$\begin{array}{cc} \; & \int_{R}\int_{R}E^{t}\left(\frac{{\tilde{h}}_{1}\left(t,S_{t-},y\right)j\left(S_{t-},{\tilde{h}}_{2}\left(t,S_{t-},y\right)\right)\left(k\right)+{\tilde{k}}_{1}\left(t,S_{t-},y\right)j\left(S_{t-},{\tilde{k}}_{2}\left(t,S_{t-},y\right)\right)\left(k\right)}{{\tilde{N}}_{t-}}\right.\\ \; & \;\;\;\;\;\;\;\;\;\left.\left.+j\left(S_{t-},{\tilde{h}}_{2}\left(t,S_{t-},y\right)\right)\left(k\right)+j\left(S_{t-},{\tilde{k}}_{2}\left(t,S_{t-},y\right)\right)\left(k\right)\right|S_{t-}=x\right)\Phi \left(t,dx\right)\bar{\nu }\left(dy\right)\\ \; & =\int_{R}\int_{R}E^{P^{t}}\left(\left.j\left(S_{t-},{\tilde{h}}_{2}\left(t,S_{t-},y\right)\right)\left(k\right)+j\left(S_{t-},{\tilde{k}}_{2}\left(t,S_{t-},y\right)\right)\left(k\right)\right|S_{t-}=x\right)\Phi \left(t,dx\right)\bar{\nu }\left(dy\right). \end{array}$$

Finally, by Lemma 10 and the above equalities,

$$\begin{array}{cc} E_{0}^{Q}T & \left[\alpha_{t}^{2}+\beta_{t}^{2}+\sigma_{t}^{2}+\gamma_{t}^{2}+\int_{|y|<1}\left({\hat{h}}_{1}^{4}\left(t,y\right)+{\hat{h}}_{2}^{4}\left(t,y\right)\right)\bar{\nu }\left(dy\right)\right.\\ \; & \left.+\int_{|y|\geq 1}\left({\hat{k}}_{1}^{4}\left(t,y\right)+{\hat{k}}_{2}^{4}\left(t,y\right)\right)\bar{\nu }\left(dy\right)+S_{t-}^{2}\left(1+\alpha_{t}^{2}+\int_{|y|<1}{\hat{h}}_{1}^{2}\left(t,y\right)\bar{\nu }\left(dy\right)\right.\right.\\ \; & \left.\left.+\int_{|y|\geq 1}{\hat{k}}_{1}^{2}\left(t,y\right)\bar{\nu }\left(dy\right)\right)+{\tilde{N}}_{t-}^{2}\left(1+S_{t-}^{2}+\sigma_{t}^{2}+\int_{|y|<1}{\hat{h}}_{2}^{2}\left(t,y\right)\bar{\nu }\left(dy\right)\right.\right.\\ \; & \left.\left.+\int_{|y|\geq 1}{\hat{k}}_{2}^{2}\left(t,y\right)\bar{\nu }\left(dy\right)\right)\right]dt=E^{Q}\left[\int_{0}^{T}H\left(t\right)dt\right]<\infty . \end{array}$$

Therefore, condition (7) holds. By virtue of the above equalities, the assumptions of Theorem 1 are satisfied, and the application of Theorem 1 in this setting then yields (88). □

In the proof of the above theorem we used Theorem 1 and the distributional approach, because the payoff of the European option is not differentiable in the strike variable. The uniqueness statements in Lemma 8 and Theorem 2 concern only the solution of the underlying SDE. We do not claim uniqueness of the distributional solution to the Dupire equation.

**Remark** **5.** *Equation *(88)* is satisfied in the sense of distribution, i.e., for each R-valued $C^{\infty }$-function f on $R^{2}$ of compact support in $\left(0,T\right)\times R$*

(89) $$\begin{array}{c} \begin{array}{cc} \; & -\int_{0}^{T}\int_{-\infty }^{\infty }f_{t}^{\prime }\left(t,k\right)C\left(t,k\right)dk\;dt=\frac{1}{2}\int_{0}^{T}\int_{-\infty }^{\infty }f\left(t,k\right)\sigma^{2}\left(t,k\right)k^{2}\frac{\partial^{2}C}{\partial k^{2}}\left(dt\;dk\right)\\ \; & +\int_{0}^{T}\int_{-\infty }^{\infty }f\left(t,k\right)kB\left(0,t\right)E^{P_{t}}\left(r_{t}1_{S_{t-}>k}\right)\;dk\;dt\\ \; & +\int_{0}^{T}\int_{-\infty }^{\infty }f\left(t,k\right)\int_{-\infty }^{\infty }\int_{-\infty }^{\infty }E^{P_{t}}\left(\left.j\left(S_{t-},{\tilde{h}}_{2}\left(t,S_{t-},y\right)\right)\left(k\right)+j\left(S_{t-},{\tilde{k}}_{2}\left(t,S_{t-},y\right)\right)\left(k\right)\right|S_{t-}=x\right)\\ \; & \;\;\;\;\;\;\;\;\;\;\;\;\Phi \left(t,dx\right)\bar{\nu }\left(dy\right)\;dk\;dt. \end{array} \end{array}$$

*In particular, $\frac{\partial^{2}C}{\partial k^{2}}\left(dt\;dk\right)=l⊗\Phi \left(dt\;dk\right)$ belongs to $D^{\prime }$ as a positive and finite Borel measure. Further details of the distributional framework employed here are provided in [19].*

The generalised Dupire formula derived in this paper is a theoretical result. The development of suitable numerical procedures for its application requires further advanced research.

**Remark** **6.** *Let Assumption 2 hold for p=4 and let the short rate $r={\left(r_{t}\right)}_{t\in T}$ be deterministic. By Assumption 2 (iv), r is càdlàg on T, hence bounded: there exists M<∞ such that $M=\sup_{t\leq T}\left|r_{t}\right|$. Therefore, the tail condition *(21)* is satisfied. Indeed,*

$$Q\left(\underset{t\leq T}{\sup}\left|r_{t}\right|>x\right)=1_{\left\{x<M\right\}},\;x\geq 0.$$

*Let $c_{r}>0$ and β>1 be arbitrary, and $C_{r}=e^{c_{r}M^{\beta }}$. Then, for x<M,*

$$C_{r}e^{-c_{r}x^{\beta }}>C_{r}e^{-c_{r}M^{\beta }}=1=1_{\left\{x<M\right\}},$$

*while for x≥M, $1_{\left\{x<M\right\}}=0<C_{r}e^{-c_{r}x^{\beta }}$.*

*Since r is deterministic, $B_{t}=\exp\left(\int_{0}^{t}r_{u}du\right)$,$B\left(0,t\right)=\exp\left(-\int_{0}^{t}r_{u}du\right)$,t∈T. Hence,*

$$\frac{dP_{t}}{dP^{*}}=\frac{1}{B\left(0,t\right)B_{t}}=1,\;P^{*}-a.s.,$$

so $P_{t}=P^{*}=Q$.

*Moreover, since $\frac{\partial C\left(t,k\right)}{\partial k}=-B\left(0,t\right)Q\left(S_{t-}>k\right)$, we have $kB\left(0,t\right)r_{t}Q\left(S_{t-}>k\right)=-r_{t}k\frac{\partial C\left(t,k\right)}{\partial k}$. Thus, *(88)* has the following form in $D^{\prime }$:*

(90) $$\begin{array}{c} \begin{array}{cc} \frac{\partial C}{\partial t} & =\frac{\sigma^{2}\left(t,k\right)k^{2}}{2}\frac{\partial^{2}C}{\partial k^{2}}-r_{t}k\frac{\partial C\left(t,k\right)}{\partial k}\\ \; & +\int_{R}\int_{R}E^{Q}\left(\left.j\left(S_{t-},{\tilde{h}}_{2}\left(t,S_{t-},y\right)\right)\left(k\right)+j\left(S_{t-},{\tilde{k}}_{2}\left(t,S_{t-},y\right)\right)\left(k\right)\right|S_{t-}=x\right)\Phi \left(t,dx\right)\bar{\nu }\left(dy\right). \end{array} \end{array}$$

*If, in addition, the Lévy measure $\bar{\nu }$ is identically zero, then *(88)* can be written in $D^{\prime }$ as follows:*

(91) $$\begin{array}{c} \begin{array}{c} \frac{\partial C}{\partial t}=\frac{\sigma^{2}\left(t,k\right)k^{2}}{2}\frac{\partial^{2}C}{\partial k^{2}}-r_{t}k\frac{\partial C\left(t,k\right)}{\partial k}, \end{array} \end{array}$$

*which corresponds to the classical Dupire Equation *(1)*.*

In the two subsequent subsections, we present theoretical examples illustrating particular cases of the general Formula (88). These examples are not intended as direct applications of the Dupire formula but rather demonstrate that the SDE structure assumed in Theorem 3, together with the tail condition for the short rate, allows the theorem to be used in various concrete situations.

### 4.1. Merton Jump-Diffusion with Vasicek Short Rate

Let the short rate ${\left(r_{t}\right)}_{t\in T}$ be described by the Vasicek model

(92) $$dr_{t}=a\left(b-r_{t}\right)dt+\sigma_{r}dW_{t},\;r_{0}\in R\;\mathrm{and}\;a,b,\sigma_{r}>0,$$

where *W* is a standard Brownian motion. No assumptions on dependence or independence between the Brownian motion *W* driving the Vasicek model and the stochastic processes introduced earlier are imposed. We have the following lemma.

**Lemma** **11.** *The short rate model given by *(92)* satisfies condition *(21)* for β=2, i.e.,*

$$Q\left(\underset{0\leq t\leq T}{\sup}\left|r_{t}\right|>x\right)\leq C_{r}\exp\left(-c_{r}x^{2}\right),\;x\geq 0.$$

The proof appears in Appendix A.

We consider the Merton jump-diffusion model as used in [8], written here in our notation and before discounting, and combined with the stochastic Vasicek short-rate model (92). The undiscounted asset price *S* is a solution to the Merton-type SDE

(93) $$dS_{t}=S_{t-}r_{t}dt+S_{t-}\sigma \left(t,S_{t-}\right)dW_{t}^{\left(2\right)}+\int_{R}S_{t-}\left(e^{y}-1\right)q\left(dt\;dy\right),\;t\in T,$$

with initial condition $S_{0}>0$ and normal distribution of jumps. The following theorem is devoted to the form of the Dupire formula in the considered case.

**Theorem** **4.** *Assume that under the measure Q the short rate r is described by the Vasicek model *(92)*. Furthermore, let the price S of the underlying asset evolve according to the following Merton-type SDE*

(94) $$\begin{array}{cc} dS_{t} & =S_{t-}r_{t}dt+S_{t-}\sigma \left(t,S_{t-}\right)dW_{t}^{\left(2\right)}+\int_{R}\tilde{h}\left(t,S_{t-},y\right)q\left(dt\;dy\right)+\int_{R}\tilde{k}\left(t,S_{t-},y\right)q\left(dt\;dy\right),\;t\in T, \end{array}$$

*with initial condition $S_{0}>0$,$\tilde{h}\left(t,x,y\right)=x\left(e^{y}-1\right)1_{\left\{|y|<1\right\}}$,$\tilde{k}\left(t,x,y\right)=x\left(e^{y}-1\right)1_{\left\{|y|\geq 1\right\}}$, and $\bar{\sigma }\left(t,x\right)=x\sigma \left(t,x\right)$ satisfying Assumption 2 (ii.b) with 2p=8 (σ is the local volatility). Additionally, let the compound Poisson process ${\left(X_{t}\right)}_{t\in T}$ have a constant rate $\overset{ˇ}{\lambda }>0$, and let the distribution of jumps $\overset{ˇ}{\varphi }$ be normal with mean $\overset{ˇ}{\mu }$ and variance ${\overset{ˇ}{\gamma }}^{2}$, and therefore, $\bar{\nu }\left(dx\right)=\overset{ˇ}{\lambda }\overset{ˇ}{\varphi }\left(dx\right)$. Then, the Dupire formula for C takes the following form in $D^{\prime }$:*

(95) $$\begin{array}{c} \begin{array}{cc} \frac{\partial C}{\partial t} & =\frac{\sigma^{2}\left(t,k\right)k^{2}}{2}\frac{\partial^{2}C}{\partial k^{2}}+kB\left(0,t\right)E^{P_{t}}\left(r_{t}1_{S_{t-}>k}\right)\\ \; & +\int_{R}e^{y}\left(C\left(t,ke^{-y}\right)-C\left(t,k\right)-\left(e^{-y}-1\right)k\frac{\partial C\left(t,k\right)}{\partial k}\right)\overset{ˇ}{\lambda }\overset{ˇ}{\varphi }\left(dy\right), \end{array} \end{array}$$

*where*

(96) $$\begin{array}{c} \begin{array}{cc} B(0,t) & =e^{m\left(0,t\right)-n\left(0,t\right)r_{0}},\;n\left(0,t\right)=\frac{1-e^{-at}}{a},\;m\left(0,t\right)=\left(b-\frac{\sigma_{r}^{2}}{2a^{2}}\right)\left(n(0,t)-t\right)-\frac{\sigma_{r}^{2}}{4a}n{\left(0,t\right)}^{2}. \end{array} \end{array}$$

**Proof.**  Clearly, $\left|h\left(t,x,y\right)|\leq \right|e^{y}-1|(1+\left|x|\right)$,$|h\left(t,x,y\right)-h\left(t,x_{1},y\right)\left|\leq \right|e^{y}-1||x-x_{1}|$, and similarly for *k*. Thus, to verify Assumption 2 (iii.b) with 2p=8, it suffices to show that $K^{\left(h\right)}\left(y\right)$$=K^{\left(k\right)}\left(y\right)$$=L^{\left(h\right)}\left(y\right)$$=L^{\left(k\right)}\left(y\right)=\left|e^{y}-1\right|\in L^{8}\left(\bar{\nu }\right)$.

If Z∼N(0,1), then $E\left[e^{\theta Z}\right]=\exp\left(\frac{1}{2}\theta^{2}\right)$ (see, e.g., [32]), and therefore, for $Z∼N(\mu ,\sigma^{2})$ and θ∈R, we have $E[e^{\theta Z}]<\infty$. Thus,

$$\int_{R}|e^{y}{-1|}^{8}\bar{\nu }\left(dy\right)=\overset{ˇ}{\lambda }\int_{R}{\left|e^{y}-1\right|}^{8}\overset{ˇ}{\varphi }\left(dy\right)\leq 2^{7}\overset{ˇ}{\lambda }\int_{R}\left(e^{8y}+1\right)\overset{ˇ}{\varphi }\left(dy\right)<\infty ,$$

which implies that $K^{\left(h\right)},K^{\left(k\right)},L^{\left(h\right)},L^{\left(k\right)}\in L^{8}\left(\bar{\nu }\right)$. Finally, *r* is continuous and adapted, and by Lemma 11, it satisfies (21) for β=2. Thus, all hypotheses of Theorem 3 hold for the considered model (94) with the Vasicek short rate (92). In line with the approach of [20], we rederive the next two formulas, which appear consecutively, so as to present a self-contained proof for the considered case.

$$\begin{array}{cc} E^{t} & \left(\left.j\left(S_{t-},{\tilde{h}}_{2}\left(t,S_{t-},y\right)\right)\left(k\right)+j\left(S_{t-},{\tilde{k}}_{2}\left(t,S_{t-},y\right)\right)\left(k\right)\right|S_{t-}=x\right)\\ \; & =E^{t}\left(\left.{\left(S_{t-}e^{y}-k\right)}_{+}-{\left(S_{t-}-k\right)}_{+}-S_{t-}\left(e^{y}-1\right)g_{S_{t-}}\left(k\right)\right|S_{t-}=x\right)\\ \; & =E^{t}\left(\left.{\left(S_{t-}e^{y}-k\right)}_{+}-{\left(S_{t-}-k\right)}_{+}-\left(e^{y}-1\right)\left[{\left(S_{t-}-k\right)}_{+}+k\right]g_{S_{t-}}\left(k\right)\right|S_{t-}=x\right)\\ \; & =E^{t}\left(\left.{\left(S_{t-}e^{y}-k\right)}_{+}-e^{y}{\left(S_{t-}-k\right)}_{+}-\left(e^{y}-1\right)kg_{S_{t-}}\left(k\right)\right|S_{t-}=x\right)\\ \; & ={\left(xe^{y}-k\right)}_{+}-e^{y}{\left(x-k\right)}_{+}-\left(e^{y}-1\right)kg_{x}\left(k\right)\\ \; & =e^{y}\left({\left(x-ke^{-y}\right)}_{+}-{\left(x-k\right)}_{+}-\left(1-e^{-y}\right)kg_{x}\left(k\right)\right), \end{array}$$

$$\begin{array}{c} \begin{array}{cc} \int_{R}\int_{R} & E^{t}\left(\left.j\left(S_{t-},{\tilde{h}}_{2}\left(t,S_{t-},y\right)\right)\left(k\right)+j\left(S_{t-},{\tilde{k}}_{2}\left(t,S_{t-},y\right)\right)\left(k\right)\right|S_{t-}=x\right)\Phi \left(t,dx\right)\bar{\nu }\left(dy\right)\\ \; & =\int_{R}\int_{R}e^{y}\left({\left(x-ke^{-y}\right)}_{+}-{\left(x-k\right)}_{+}-\left(1-e^{-y}\right)kg_{x}\left(k\right)\right)\Phi \left(t,dx\right)\bar{\nu }\left(dy\right)\\ \; & =\int_{R}e^{y}E^{Q}{\tilde{N}}_{t-}\int_{R}\left({\left(x-ke^{-y}\right)}_{+}-{\left(x-k\right)}_{+}-\left(1-e^{-y}\right)kg_{x}\left(k\right)\right)\mu_{S_{t-}}^{t}\left(dx\right)\bar{\nu }\left(dy\right)\\ \; & =\int_{R}e^{y}\left(E^{Q}{\tilde{N}}_{t-}\int_{R}{\left(x-ke^{-y}\right)}_{+}\mu_{S_{t-}}^{t}\left(dx\right)-E^{Q}{\tilde{N}}_{t-}\int_{R}{\left(x-k\right)}_{+}\mu_{S_{t-}}^{t}\left(dx\right)\right.\\ \; & \left.-\left(1-e^{-y}\right)kE^{Q}{\tilde{N}}_{t-}\int_{R}g_{x}\left(k\right)\mu_{S_{t-}}^{t}\left(dx\right)\right)\bar{\nu }\left(dy\right)\\ \; & =\int_{R}e^{y}\left(E^{Q}{\tilde{N}}_{t-}E^{t}{\left(S_{t-}-ke^{-y}\right)}_{+}-E^{Q}{\tilde{N}}_{t-}E^{t}{\left(S_{t-}-k\right)}_{+}\right.\\ \; & \left.-\left(1-e^{-y}\right)kE^{Q}{\tilde{N}}_{t-}E^{t}1_{S_{t-}>k}\right)\bar{\nu }\left(dy\right)\\ \; & =\int_{R}e^{y}\left(E^{Q}R_{t}{\left(S_{t-}-ke^{-y}\right)}_{+}-E^{Q}R_{t}{\left(S_{t-}-k\right)}_{+}-\left(e^{-y}-1\right)k\frac{\partial C\left(t,k\right)}{\partial k}\right)\bar{\nu }\left(dy\right)\\ \; & =\int_{R}e^{y}\left(C\left(t,ke^{-y}\right)-C\left(t,k\right)-\left(e^{-y}-1\right)k\frac{\partial C\left(t,k\right)}{\partial k}\right)\overset{ˇ}{\lambda }\overset{ˇ}{\varphi }\left(dy\right). \end{array} \end{array}$$

Hence, the Formula (88) takes the form (95), which completes the proof. Finally, (96) is the price of the zero-coupon bond in the Vasicek short-rate model (see, e.g., [2] (Eqn. (3.8))), adapted to our notation. □

### 4.2. Jump-Diffusion with Time-Dependent Jump Scaling

We assume that the (undiscounted) asset price *S* is a solution to the following SDE

(97) $$dS_{t}=S_{t-}r_{t}dt+S_{t-}\sigma \left(t,S_{t-}\right)dW_{t}^{\left(2\right)}+\int_{R}S_{t-}\left(e^{a(t)y}-1\right)q\left(dt\;dy\right),\;t\in T,$$

with initial condition $S_{0}>0$ and a deterministic function a(t), scaling the instantaneous log-return. We assume generally that the measure $\bar{\nu }$ satisfies Assumption 2 (i) and the short rate *r* satisfies Assumption 2 (iv) and (21). If Assumption 2 (ii)–(iii) holds for 2p=8, then the full set of hypotheses of Theorem 3 is satisfied for p=4. Consequently, Theorem 3 can be used in the considered case. The following theorem is devoted to the form of the Dupire formula for this model.

**Theorem** **5.** *Under Q, let the short rate r be adapted, càdlàg, and satisfy *(21)*, and let the price process of the underlying asset $S={\left\{S_{t}\right\}}_{t\in T}$ be given by*

(98) $$\begin{array}{cc} dS_{t} & =S_{t-}r_{t}dt+S_{t-}\sigma \left(t,S_{t-}\right)dW_{t}^{\left(2\right)}+\int_{R}\tilde{h}\left(t,S_{t-},y\right)q\left(dt\;dy\right)+\int_{R}\tilde{k}\left(t,S_{t-},y\right)q\left(dt\;dy\right),\;t\in T, \end{array}$$

*with initial condition $S_{0}>0$, $\tilde{h}\left(t,x,y\right)=x\left(e^{a(t)y}-1\right)1_{\left\{|y|<1\right\}}$, $\tilde{k}\left(t,x,y\right)=x\left(e^{a(t)y}-1\right)1_{\left\{|y|\geq 1\right\}}$, a:T→R being a deterministic, continuous time-dependent jump-size scaling function, and $\bar{\sigma }\left(t,x\right)=x\sigma \left(t,x\right)$ satisfying Assumption 2 (ii) with p=4. Moreover, we assume that $\bar{\nu }$ is finite, and the following inequality holds*

(99) $$\begin{array}{c} \int_{R}{\left(\underset{t\in T}{\sup}\left|e^{a(t)y}-1\right|\right)}^{8}\bar{\nu }\left(dy\right)<\infty . \end{array}$$

*Then, the Dupire formula for C takes the following form in $D^{\prime }$*

(100) $$\begin{array}{c} \begin{array}{cc} \frac{\partial C}{\partial t} & =\frac{\sigma^{2}\left(t,k\right)k^{2}}{2}\frac{\partial^{2}C}{\partial k^{2}}+kB\left(0,t\right)E^{P_{t}}\left(r_{t}1_{S_{t-}>k}\right)\\ \; & +\int_{R}e^{a(t)y}\left(C\left(t,ke^{-a(t)y}\right)-C\left(t,k\right)-\left(e^{-a(t)y}-1\right)k\frac{\partial C\left(t,k\right)}{\partial k}\right)\bar{\nu }\left(dy\right). \end{array} \end{array}$$

**Proof.**  We have the inequalities $\left|h\left(t,x,y\right)\right|\leq \sup_{t\in T}|e^{a(t)y}-1|(1+\left|x|\right)$ and $|h\left(t,x,y\right)-h\left(t,x_{1},y\right)|\leq \sup_{t\in T}|e^{a(t)y}-1||x-x_{1}|$ for all $x,x_{1}\in R$ and y∈R, and similarly for *k*. Therefore, $K^{\left(h\right)}\left(y\right)=K^{\left(k\right)}\left(y\right)=L^{\left(h\right)}\left(y\right)=L^{\left(k\right)}\left(y\right)=\sup_{t\in T}\left|e^{a(t)y}-1\right|$ and (99) implies $K^{\left(h\right)},K^{\left(k\right)},L^{\left(h\right)},L^{\left(k\right)}\in L^{8}\left(\bar{\nu }\right)$. Thus, Assumption 2 (iii) holds for p=4.

Proceeding in the same manner as in the previous proof and in [20], we now derive the next two consecutive formulas in the present, more general setting.

$$\begin{array}{cc} E^{t} & \left(\left.j\left(S_{t-},{\tilde{h}}_{2}\left(t,S_{t-},y\right)\right)\left(k\right)+j\left(S_{t-},{\tilde{k}}_{2}\left(t,S_{t-},y\right)\right)\left(k\right)\right|S_{t-}=x\right)\\ \; & =E^{t}\left(\left.{\left(S_{t-}e^{a(t)y}-k\right)}_{+}-{\left(S_{t-}-k\right)}_{+}-S_{t-}\left(e^{a(t)y}-1\right)g_{S_{t-}}\left(k\right)\right|S_{t-}=x\right)\\ \; & =E^{t}\left(\left.{\left(S_{t-}e^{a(t)y}-k\right)}_{+}-{\left(S_{t-}-k\right)}_{+}-\left(e^{a(t)y}-1\right)\left[{\left(S_{t-}-k\right)}_{+}+k\right]g_{S_{t-}}\left(k\right)\right|S_{t-}=x\right)\\ \; & =E^{t}\left(\left.{\left(S_{t-}e^{a(t)y}-k\right)}_{+}-e^{a(t)y}{\left(S_{t-}-k\right)}_{+}-\left(e^{a(t)y}-1\right)kg_{S_{t-}}\left(k\right)\right|S_{t-}=x\right)\\ \; & ={\left(xe^{a(t)y}-k\right)}_{+}-e^{a(t)y}{\left(x-k\right)}_{+}-\left(e^{a(t)y}-1\right)kg_{x}\left(k\right)\\ \; & =e^{a(t)y}\left({\left(x-ke^{-a(t)y}\right)}_{+}-{\left(x-k\right)}_{+}-\left(1-e^{-a(t)y}\right)kg_{x}\left(k\right)\right), \end{array}$$

(101) $$\begin{array}{c} \begin{array}{cc} \int_{R}\int_{R} & E^{t}\left(\left.j\left(S_{t-},{\tilde{h}}_{2}\left(t,S_{t-},y\right)\right)\left(k\right)+j\left(S_{t-},{\tilde{k}}_{2}\left(t,S_{t-},y\right)\right)\left(k\right)\right|S_{t-}=x\right)\Phi \left(t,dx\right)\bar{\nu }\left(dy\right)\\ \; & =\int_{R}\int_{R}e^{a(t)y}\left({\left(x-ke^{-a(t)y}\right)}_{+}-{\left(x-k\right)}_{+}-\left(1-e^{-a(t)y}\right)kg_{x}\left(k\right)\right)\Phi \left(t,dx\right)\bar{\nu }\left(dy\right)\\ \; & =\int_{R}e^{a(t)y}E^{Q}{\tilde{N}}_{t-}\int_{R}\left({\left(x-ke^{-a(t)y}\right)}_{+}-{\left(x-k\right)}_{+}-\left(1-e^{-a(t)y}\right)kg_{x}\left(k\right)\right)\mu_{S_{t-}}^{t}\left(dx\right)\bar{\nu }\left(dy\right)\\ \; & =\int_{R}e^{a(t)y}\left(E^{Q}{\tilde{N}}_{t-}\int_{R}{\left(x-ke^{-a(t)y}\right)}_{+}\mu_{S_{t-}}^{t}\left(dx\right)-E^{Q}{\tilde{N}}_{t-}\int_{R}{\left(x-k\right)}_{+}\mu_{S_{t-}}^{t}\left(dx\right)\right.\\ \; & \left.-\left(1-e^{-a(t)y}\right)kE^{Q}{\tilde{N}}_{t-}\int_{R}g_{x}\left(k\right)\mu_{S_{t-}}^{t}\left(dx\right)\right)\bar{\nu }\left(dy\right)\\ \; & =\int_{R}e^{a(t)y}\left(E^{Q}{\tilde{N}}_{t-}E^{t}{\left(S_{t-}-ke^{-a(t)y}\right)}_{+}-E^{Q}{\tilde{N}}_{t-}E^{t}{\left(S_{t-}-k\right)}_{+}\right.\\ \; & \left.-\left(1-e^{-a(t)y}\right)kE^{Q}{\tilde{N}}_{t-}E^{t}1_{S_{t-}>k}\right)\bar{\nu }\left(dy\right)\\ \; & =\int_{R}e^{a(t)y}\left(E^{Q}R_{t}{\left(S_{t-}-ke^{-a(t)y}\right)}_{+}-E^{Q}R_{t}{\left(S_{t-}-k\right)}_{+}-\left(e^{-a(t)y}-1\right)k\frac{\partial C\left(t,k\right)}{\partial k}\right)\bar{\nu }\left(dy\right)\\ \; & =\int_{R}e^{a(t)y}\left(C\left(t,ke^{-a(t)y}\right)-C\left(t,k\right)-\left(e^{-a(t)y}-1\right)k\frac{\partial C\left(t,k\right)}{\partial k}\right)\bar{\nu }\left(dy\right). \end{array} \end{array}$$

Because *r* satisfies (21), the assumptions of Theorem 3 are fulfilled. Together with Formula (101), this allows us to apply Theorem 3 and obtain (100). □

The financial interpretation of the jump part is the following: at a jump time with mark *y*, we have $S_{t}=S_{t-}e^{a(t)y}$, so the function a(t) scales the instantaneous log-return generated by a jump of size *y*. Therefore, the considered model introduces time-dependent jump-size scaling.

### 4.3. On Minimal Entropy Martingale Measures and Their Relevance

We begin by recalling the notion of a minimal entropy martingale measure, which appears naturally in models of the type considered in Theorem 3.

**Definition** **2.** *Let $\left(\Omega ,F,{\left(F_{t}\right)}_{t\in T},P\right)$ be a filtered probability space satisfying the usual conditions. Let $\tilde{S}={\left({\tilde{S}}_{t}\right)}_{t\in T}$ be an adapted one-dimensional discounted price process. A probability measure Q on $\left(\Omega ,F_{T}\right)$ is called an equivalent local martingale measure (ELMM) for $\tilde{S}$ if Q∼P and $\tilde{S}$ is a local Q-martingale on T. For any Q≪P, the relative entropy of Q with respect to P is defined by*

$$H\left(Q|P\right)=E^{P}\left[\frac{dQ}{dP}\log\left(\frac{dQ}{dP}\right)\right],$$

*and we set H(Q∣P)=+∞ in cases where Q is not absolutely continuous with respect to P.*

*An ELMM $Q^{E}$ for $\tilde{S}$ on T is called a minimal entropy martingale measure (MEMM) if it is the element of the class of ELMMs that attains the minimal value of the functional H(Q∣P).*

The above formulation follows the definition given by Schweizer [33]. Moreover, it is worth noting that if $\tilde{S}$ is a nonnegative P-semimartingale, the fundamental theorem of asset pricing implies that the existence of an ELMM for $\tilde{S}$ is equivalent to the absence of arbitrage in the sense of NFLVR (no free lunch with vanishing risk), see [33].

The minimal entropy martingale measure is often regarded as a natural and canonical choice of pricing measure in incomplete markets, and its justification can be expressed in information-theoretic terms. Miyahara [34] characterises the MEMM as the equivalent martingale measure closest to the original probability P in the sense of Kullback–Leibler distance, interpreting it as a typical example of a minimal distance martingale measure. Large deviation theory is closely related to the minimisation of relative entropy, and Sanov’s theorem provides the probabilistic foundation for interpreting the MEMM as the most probable empirical measure of price paths within the class of equivalent martingale measures, see Cover and Thomas [35] and Miyahara [34]. This perspective highlights the MEMM as a statistically natural and information-theoretically justified representative among all equivalent martingale measures.

In the context of proving the existence of the minimal entropy martingale measure, Frittelli [36] formulates his construction for a family of adapted processes. In the single-process case relevant here, with one adapted process *X*, we consider the space

$$K=\mathrm{Lin}\left\{H\left(X_{t}-X_{s}\right):H\in L^{\infty }\left(\Omega ,F_{s},P\right),s\leq t\right\},$$

and define

$$M=\left\{Q\ll P:E_{Q}\left[k\right]=0\;\mathrm{for}\;\mathrm{all}\;k\in K\right\},$$

that is, the class of P-absolutely continuous probability measures under which *X* is a Q-martingale. The minimal entropy martingale measure is defined as a minimiser of H(Q∣P) over M. Under the assumptions that *X* is bounded and that the infimum of H(Q∣P) over M is finite, Frittelli shows that a unique minimiser exists (see [36] (Thm. 2.1)). In addition, once this minimiser is known to exist, ref. [36] (Thm. 2.2) shows that the existence of at least one equivalent martingale measure with finite entropy means that the minimiser itself must be equivalent to P. Ref. [36] (Remark 2.1) explains that if *X* is locally bounded, ref. [36] (Thm. 2.1) holds with local martingale measures. In turn, for unbounded *X*, the infimum of H(Q∣P) over M may fail to be attained. For the present class of models, existence of a MEMM under a physical measure P is not established here and would require additional assumptions.

The existence problem for the minimal entropy martingale measure has been solved in the case where the price process is a geometric Lévy model, that is, when $S_{t}=S_{0}e^{Z_{t}}$, where $S_{0}$ is a positive constant, *Z* is a Lévy process under P with the generating triplet $\left(\sigma^{2},\nu \left(dx\right),b\right)$, and the short rate r>0 is constant. Miyahara [34] (Theorem 7.1) provides a sufficient condition for the existence of the minimal entropy martingale measure in this model.

The model developed in Section 4 is constructed directly under an equivalent martingale measure Q. Therefore, the question of minimal entropy with respect to the physical measure P does not arise in the case of our model. Introducing the minimal entropy martingale measure would require reformulating the entire model under P and analysing the resulting semimartingale structure with respect to relative entropy. Such an investigation would demand additional, model-specific assumptions and lies outside the scope of the present paper. A thorough study of MEMM for this class of models is planned as part of our future research.

## 5. Conclusions

The Dupire formula serves as a practical instrument for valuing financial derivatives. In this paper, we derived the Dupire formula in a setting that allows general jump behaviour and a class of stochastic short-rate models including the Vasicek model. A key step was proving existence and uniqueness for the asset-price SDE with an exogenous stochastic short rate. We also obtained more explicit versions of the Dupire formula in two modelling examples, showing how the general result can be used in practice. A natural direction for further research is to extend the Dupire formula to models in which the underlying Lévy measure is infinite, so that the asset dynamics exhibit jumps of infinite activity.

## Data Availability

No new data were created or analyzed in this study.

## Appendix A. Proofs of Propositions and Lemmas

**Proof** **of Proposition 2.** For $Z=\sup_{t\in T}\left|r_{t}\right|$, ref. [37] (Theorem 12.1) implies

(A1) $$E\left[Z^{q}\right]=\int_{0}^{\infty }qx^{q-1}Q\left(Z>x\right)dx\leq qC_{r}\int_{0}^{\infty }x^{q-1}e^{-c_{r}x^{\beta }}dx<\infty ,$$

which yields (22). In turn, by (A1), for each *t*, $E|r_{t}{|}^{q}\leq E\left[Z^{q}\right]<\infty$. Hence,

$$\int_{0}^{T}E{\left|r_{t}\right|}^{q}dt=E\left[\int_{0}^{T}{\left|r_{t}\right|}^{q}dt\right]\leq TE\left[Z^{q}\right]<\infty .$$

□

**Proof** **of Lemma 1.** Let q≥1 and $Z=\sup_{t\leq T}\left|r_{t}\right|$. By (21), $Q\left(Z>z\right)\leq C_{r}e^{-c_{r}z^{\beta }}$, z≥0, and $\sup_{t\leq T}{\left|R_{t}\right|}^{q}\leq e^{qTZ}$ a.s. Therefore, $E[\sup_{t\leq T}|R_{t}{|}^{q}]\leq E\left[e^{qTZ}\right]$. Integrating by substitution $y=e^{qTz}$, we obtain

$$\begin{array}{cc} Ee^{qTZ} & =\int_{0}^{\infty }Q\left(e^{qTZ}>y\right)dy=1+qT\int_{0}^{\infty }e^{qTz}Q\left(Z>z\right)dz\leq 1+qTC_{r}I, \end{array}$$

where $I=\int_{0}^{\infty }e^{qTz-c_{r}z^{\beta }}dz$. Let $R^{\prime }>0$ be such that for $z\geq R^{\prime }$, we have the inequality $1-\frac{qT}{c_{r}}z^{1-\beta }\geq \frac{1}{2}$. Then, $\int_{R^{\prime }}^{\infty }e^{qTz-c_{r}z^{\beta }}dz=\int_{R^{\prime }}^{\infty }e^{-c_{r}z^{\beta }\left(1-\frac{qT}{c_{r}}z^{1-\beta }\right)}dz\leq \int_{R^{\prime }}^{\infty }e^{-\left(c_{r}/2\right)z^{\beta }}dz<\infty$ and as a consequence, I<∞. Thus, $E[\sup_{t\leq T}|R_{t}{|}^{q}]\leq Ee^{qTZ}\leq 1+qTC_{r}I<\infty$ and therefore, $R\in \Lambda^{q}\left(T\right)$. □

**Proof** **of Lemma 2.** Clearly, $\theta_{t-}$ and $r_{t-}$ are predictable, and thus, all integrands in the process $\int_{0}^{\cdot }X\left(\theta_{s-},ds\right)$ are also predictable. We define the stopping times

$$\tau_{m}=\inf\{t\geq 0:|\theta_{t-}\left|\geq m\right\}\wedge T,\;m\in N.$$

On $\left[0,\tau_{m}\right]$, we carry out the next part of the proof. Since *r* is càdlàg, we have $r_{s-}=r_{s}$ for almost all s∈T. Hence, by the Fubini theorem and the assumption $E[\sup_{t\leq T}|r_{t}|]<\infty$, we obtain

$$E\left[\int_{0}^{T}\left|r_{s-}\right|ds\right]=E\left[\int_{0}^{T}\left|r_{s}\right|ds\right]=\int_{0}^{T}E\left|r_{s}\right|ds\leq TE\left[\underset{t\leq T}{\sup}\left|r_{t}\right|\right]<\infty .$$

Consequently, $\int_{0}^{T}\left|r_{s-}\right|ds<\infty$ a.s. and, since $|\theta_{s-}|\leq m$ on $\left[0,\tau_{m}\right]$,

$$\int_{0}^{\tau_{m}}|b\left(\theta_{s-},s\right)|ds=\int_{0}^{\tau_{m}}|\theta_{s-}r_{s-}|ds\leq m\int_{0}^{T}\left|r_{s-}\right|ds<\infty \;a.s.$$

Furthermore, $|f\left(\theta_{s-},s\right){|}^{2}$$=(|f\left(\theta_{s-},s\right){{|}^{p})}^{2/p}\leq C_{\sigma }^{2/p}{\left(1+m^{p}\right)}^{2/p}\leq C_{\sigma }^{2/p}{\left(2m^{p}\right)}^{2/p}$$\leq 2C_{\sigma }^{2/p}\left(1+m^{2}\right)<\infty$ a.s. Thus, since

$$E\left[\int_{0}^{\tau_{m}}{\left|f\left(\theta_{s-},s\right)\right|}^{2}ds\right]\leq 2C_{\sigma }^{2/p}\left(1+m^{2}\right)T<\infty ,$$

the integral $\int_{0}^{t\wedge \tau_{m}}f\left(\theta_{s-},s\right)dW_{s}^{\left(2\right)}$ is a square-integrable martingale. By the Hölder inequality,

$$\int_{R}{\left(K^{\left(h\right)}\left(y\right)\right)}^{2}\bar{\nu }\left(dy\right)\leq {\left(\int_{R}{\left|K^{\left(h\right)}\left(y\right)\right|}^{p}\bar{\nu }\left(dy\right)\right)}^{2/p}{\left(\bar{\nu }\left(R\right)\right)}^{1-2/p}<\infty$$

and similarly, $\int_{R}{\left(K^{\left(k\right)}\left(y\right)\right)}^{2}\bar{\nu }\left(dy\right)<\infty$. Thus, $\int_{0}^{t\wedge \tau_{m}}\int_{R}\tilde{h}\left(s,\theta_{s-},y\right)q\left(ds\;dy\right)$ and $\int_{0}^{t\wedge \tau_{m}}\int_{R}\tilde{k}\left(s,\theta_{s-},y\right)q\left(ds\;dy\right)$ are square-integrable martingales, since we have

$$E\left[\int_{0}^{\tau_{m}}\int_{R}{\left|\tilde{h}\left(s,\theta_{s-},y\right)\right|}^{2}\bar{\nu }\left(dy\right)ds\right]\leq {\left(1+m\right)}^{2}T\int_{R}{\left(K^{\left(h\right)}\left(y\right)\right)}^{2}\bar{\nu }\left(dy\right)<\infty$$

and similarly $E\left[\int_{0}^{\tau_{m}}\int_{R}{\left|\tilde{k}\left(s,\theta_{s-},y\right)\right|}^{2}\bar{\nu }\left(dy\right)ds\right]<\infty$. The above properties imply that $Z_{t}^{\left(m\right)}=\int_{0}^{t\wedge \tau_{m}}X\left(\theta_{s-},ds\right),\;t\in T,\;m\in N,$ are semimartingales (in particular, they are adapted and càdlàg). Moreover, ${\left\{\tau_{m}\right\}}_{m=1}^{\infty }$ is nondecreasing and since for $m^{\prime }>m$, the integrands coincide on $\left[0,\tau_{m}\right]$, we have $Z_{t}^{\left(m\right)}=Z_{t}^{\left(m^{\prime }\right)},\;t\leq \tau_{m}$. It enables us to define an adapted and càdlàg process *Z* on T, independent of *m*, by the equality $Z_{t}=Z_{t}^{\left(m\right)}$ for $t\leq \tau_{m}$. Moreover, $Z_{t\wedge \tau_{m}}=Z_{t}^{\left(m\right)}$ for all *m* a.s., i.e., ${\left\{\tau_{m}\right\}}_{m=1}^{\infty }$ localises *Z*. The fact that the class of semimartingales is closed under localisation, implies that *Z* is a semimartingale on T. Finally, the decomposition

$$\begin{array}{c} \begin{array}{cc} Z_{t\wedge \tau_{m}} & =\int_{0}^{t\wedge \tau_{m}}b\left(\theta_{s-},s\right)ds+\int_{0}^{t\wedge \tau_{m}}f\left(\theta_{s-},s\right)dW_{s}^{\left(2\right)}+\int_{0}^{t\wedge \tau_{m}}\int_{R}\tilde{h}\left(s,\theta_{s-},y\right)q\left(ds\;dy\right)\\ \; & +\int_{0}^{t\wedge \tau_{m}}\int_{R}\tilde{k}\left(s,\theta_{s-},y\right)q\left(ds\;dy\right)\;a.s.,\;m\in N, \end{array} \end{array}$$

and the fact that $\tau_{m}↑T$ imply the following form of *Z*

$$\begin{array}{c} \begin{array}{cc} Z_{t} & =\int_{0}^{t}b\left(\theta_{s-},s\right)ds+\int_{0}^{t}f\left(\theta_{s-},s\right)dW_{s}^{\left(2\right)}+\int_{0}^{t}\int_{R}\tilde{h}\left(s,\theta_{s-},y\right)q\left(ds\;dy\right)\\ \; & +\int_{0}^{t}\int_{R}\tilde{k}\left(s,\theta_{s-},y\right)q\left(ds\;dy\right)\;a.s. \end{array} \end{array}$$

□

**Proof** **of Lemma 3.** We introduce the notation $S_{cl}=\sup_{s_{1}\leq t\leq s_{2}}\left|M_{t}\right|$,$S_{op}=\sup_{s_{1}<t\leq s_{2}}\left|M_{t}\right|$. Since $\left(s_{1},s_{2}\right]\subset \left[s_{1},s_{2}\right]$, we have $S_{op}\leq S_{cl}$. To prove the reverse inequality, we fix $t\in [s_{1},s_{2}]$. If $t>s_{1}$, then $t\in (s_{1},s_{2}]$, hence $|M_{t}|\leq S_{op}$. If $t=s_{1}$, then by right-continuity of *M* there exists a sequence $t_{n}↓s_{1}$ with $t_{n}>s_{1}$ such that $M_{s_{1}}=\lim_{n\to \infty }M_{t_{n}}$. Therefore, $|M_{s_{1}}|=\lim_{n\to \infty }\left|M_{t_{n}}\right|\leq S_{op}$. Since $|M_{t}|\leq S_{op}$ for all $t\in [s_{1},s_{2}]$, we obtain $S_{cl}\leq S_{op}$. Together with the opposite inequality, this yields $\sup_{s_{1}\leq t\leq s_{2}}|M_{t}|=\sup_{s_{1}<t\leq s_{2}}\left|M_{t}\right|$ a.s. □

**Proof** **of Lemma 5.** We define stopping times

$$\tau_{n}=\inf\left\{s\geq 0:\int_{0}^{s}\int_{R}\left({\left|g\left(r,y\right)\right|}^{2}+{\left|g\left(r,y\right)\right|}^{p}\right)\bar{\nu }\left(dy\right)dr>n\right\}\wedge t,\;n\in N.$$

For almost every ω, the map $s⟼\int_{0}^{s}\int_{R}{\left(|g\left(r,y,\omega \right)\right|}^{2}+{\left|g\left(r,y,\omega \right)\right|}^{p})\bar{\nu }\left(dy\right)dr$ is finite and nondecreasing on T and $A\left(\omega \right)=\int_{0}^{t}\int_{R}{\left(|g\left(r,y,\omega \right)\right|}^{2}+{\left|g\left(r,y,\omega \right)\right|}^{p})\bar{\nu }\left(dy\right)dr<\infty$. If n≥A(ω), then $\tau_{n}\left(\omega \right)=t$ and therefore, $\tau_{n}\left(\omega \right)↑t$ for almost every ω. Let

$$g^{\left(n\right)}\left(r,y\right)=g\left(r,y\right)1_{\left\{r\leq \tau_{n}\right\}},\;Y^{\left(n\right)}\left(s\right)=\int_{0}^{s}\int_{R}g^{\left(n\right)}\left(r,y\right)q\left(dr\;dy\right)=\int_{0}^{s\wedge \tau_{n}}\int_{R}g\left(r,y\right)q\left(dr\;dy\right).$$

Then, Q-a.s., $\int_{0}^{t}\int_{R}{\left|g^{\left(n\right)}\left(r,y,\omega \right)\right|}^{2}\bar{\nu }\left(dy\right)dr\leq n$,$\int_{0}^{t}\int_{R}{\left|g^{\left(n\right)}\left(r,y,\omega \right)\right|}^{p}\bar{\nu }\left(dy\right)dr\leq n$. Hence, $Y^{\left(n\right)}$ is a square-integrable martingale on T. By Lemma 4,

(A2) $$E\left[\underset{0<s\leq t}{\sup}{\left|Y^{\left(n\right)}\left(s\right)\right|}^{p}\right]\leq c_{7}E\left[{\left(\int_{0}^{t}\int_{R}{\left|g^{\left(n\right)}\left(r,y\right)\right|}^{2}\bar{\nu }\left(dy\right)dr\right)}^{p/2}\right]+c_{8}E\left[\int_{0}^{t}\int_{R}{\left|g^{\left(n\right)}\left(r,y\right)\right|}^{p}\bar{\nu }\left(dy\right)dr\right].$$

Since $\tau_{n}↑t$ and $Y^{\left(n\right)}\left(s\right)=Y\left(s\wedge \tau_{n}\right)$, we have $\sup_{0<s\leq t}|Y^{\left(n\right)}\left(s\right)|↑\sup_{0<s\leq t}\left|Y\left(s\right)\right|$ a.s. and by monotone convergence, $E[\sup_{0<s\leq t}{\left|Y\left(s\right)\right|}^{p}]=\lim_{n\to \infty }E[\sup_{0<s\leq t}|Y^{\left(n\right)}\left(s\right){|}^{p}]$. Moreover, since $|g^{\left(n\right)}\left|↑|g\right|$ pointwise, $\int_{0}^{t}\int_{R}|g^{\left(n\right)}{\left(r,y\right)|}^{2}\bar{\nu }\left(dy\right)dr↑\int_{0}^{t}\int_{R}{\left|g\left(r,y\right)\right|}^{2}\bar{\nu }\left(dy\right)dr$,$\int_{0}^{t}\int_{R}|g^{\left(n\right)}{\left(r,y\right)|}^{p}\bar{\nu }\left(dy\right)dr↑\int_{0}^{t}\int_{R}{\left|g\left(r,y\right)\right|}^{p}\bar{\nu }\left(dy\right)dr$, and all terms are nonnegative. Thus, by monotone convergence, $\lim_{n\to \infty }E[(\int_{0}^{t}\int_{R}|g^{\left(n\right)}{|}^{2}\bar{\nu }{\left(dy\right)dr)}^{p/2}]=E[(\int_{0}^{t}\int_{R}{\left|g\right|}^{2}\bar{\nu }\left(dy\right)dr{)}^{p/2}]$, and $\lim_{n\to \infty }E[\int_{0}^{t}\int_{R}|g^{\left(n\right)}{|}^{p}\;\bar{\nu }\left(dy\right)dr]=E[\int_{0}^{t}\int_{R}{\left|g\right|}^{p}\bar{\nu }\left(dy\right)dr]$. Therefore, passing to the limit n→∞ in (A2) gives (29). □

**Proof** **of Lemma 6.** The inequality for p=2 follow from (26) with ${\hat{C}}_{2}=C_{2}$. Let p>2 be fixed and let x∈R. By the Young inequality,

$$\begin{array}{cc} {\left|x\right|}^{p-2}{\left|\tilde{h}\left(s,x,y,\omega \right)\right|}^{2} & \leq \frac{p-2}{p}{\left({\left|x\right|}^{p-2}\right)}^{\frac{p}{p-2}}+\frac{2}{p}{\left({\left|\tilde{h}\left(s,x,y,\omega \right)\right|}^{2}\right)}^{\frac{p}{2}}=\frac{p-2}{p}{\left|x\right|}^{p}\\ \; & +\frac{2}{p}{\left|\tilde{h}\left(s,x,y,\omega \right)\right|}^{p} \end{array}$$

and therefore, by (26) for ${\hat{C}}_{p}=\frac{p-2}{p}\bar{\nu }\left(R\right)+\frac{2}{p}C_{p}$,

$$\begin{array}{cc} \int_{R}{\left|x\right|}^{p-2}{\left|\tilde{h}\left(s,x,y,\omega \right)\right|}^{2}\bar{\nu }\left(dy\right) & \leq \frac{p-2}{p}{\left|x\right|}^{p}\int_{R}\bar{\nu }\left(dy\right)+\frac{2}{p}\int_{R}{\left|\tilde{h}\left(s,x,y,\omega \right)\right|}^{p}\bar{\nu }\left(dy\right)\\ \; & \leq {\hat{C}}_{p}\left(1+{\left|x\right|}^{p}\right). \end{array}$$

Proceeding in the case 2p in the same way as for *p* and applying (27), we obtain

$$\begin{array}{c} \int_{R}{\left|x\right|}^{2p-2}{\left|\tilde{h}\left(s,x,y,\omega \right)\right|}^{2}\bar{\nu }\left(dy\right)\leq {\hat{C}}_{2p}\left(1+{\left|x\right|}^{2p}\right), \end{array}$$

with ${\hat{C}}_{2p}=\frac{2p-2}{2p}\bar{\nu }\left(R\right)+\frac{2}{2p}C_{2p}$. □

**Proof** **of Lemma 7.** For q=2, we have

$$\begin{array}{cc} \left|{\left|x+u\right|}^{2}-{\left|x\right|}^{2}\right| & =\left|2xu+u^{2}\right|\leq 2|x||u|+{\left|u\right|}^{2}\leq 2\left(\left|x||u\right|+{\left|u\right|}^{2}\right). \end{array}$$

For q>2, the Taylor theorem with Lagrange remainder implies the existence of θ∈(0,1) such that ${\left|x+u\right|}^{q}={\left|x\right|}^{q}+{q|x|}^{q-2}xu+\frac{1}{2}q\left(q-1\right){\left|x+\theta u\right|}^{q-2}u^{2}$. Thus,

(A3) $$\begin{array}{c} \begin{array}{cc} \left|{\left|x+u\right|}^{q}\right. & \left.-{\left|x\right|}^{q}-q{\left|x\right|}^{q-2}xu\right|=\frac{1}{2}{q\left(q-1\right)|x+\theta u|}^{q-2}{\left|u\right|}^{2}\\ \; & \leq \frac{1}{2}q\left(q-1\right){\left(|x|+|u|\right)}^{q-2}{\left|u\right|}^{2}\leq \frac{1}{2}2^{q-3}q\left(q-1\right)\left({\left|x\right|}^{q-2}+{\left|u\right|}^{q-2}\right){\left|u\right|}^{2}\\ \; & =2^{q-4}q\left(q-1\right)\left({\left|x\right|}^{q-2}{\left|u\right|}^{2}+{\left|u\right|}^{q}\right). \end{array} \end{array}$$

If |u|≤|x|, then ${\left|x\right|}^{q-2}{\left|u\right|}^{2}\leq {\left|x\right|}^{q-1}\left|u\right|$, and if |u|≥|x|, then ${\left|x\right|}^{q-2}{\left|u\right|}^{2}\leq {\left|u\right|}^{q}$. Hence,

$$\begin{array}{c} {\left|x\right|}^{q-2}{\left|u\right|}^{2}\leq {\left|x\right|}^{q-1}\left|u\right|+{\left|u\right|}^{q}. \end{array}$$

Thus, by (A3), $\left|{\left|x+u\right|}^{q}-{\left|x\right|}^{q}-q{\left|x\right|}^{q-2}xu\right|\leq 2^{q-3}q\left(q-1\right)\left({\left|x\right|}^{q-1}\left|u\right|+{\left|u\right|}^{q}\right)$. □

**Proof** **of Lemma 9.** We write the Equation (13) in the convention used in [29].

$$dS_{t}=G_{S}\left(t\right)dt+F_{S}\left(t\right)dW_{t}^{\left(2\right)}+\int_{|y|<1}H_{S}\left(t,y\right)q\left(dt\;dy\right)+\int_{|y|\geq 1}K_{S}\left(t,y\right)N\left(dt\;dy\right),$$

$$G_{S}\left(t\right)=S_{t-}r_{t-}-\int_{|y|\geq 1}{\tilde{k}}_{2}\left(t,S_{t-},y\right)\bar{\nu }\left(dy\right),\;F_{S}\left(t\right)=S_{t-}\sigma \left(t,S_{t-}\right),$$

$$H_{S}\left(t,y\right)={\tilde{h}}_{2}\left(t,S_{t-},y\right),\;K_{S}\left(t,y\right)={\tilde{k}}_{2}\left(t,S_{t-},y\right),$$

$$B_{t}=\exp\left(\int_{0}^{t}r_{u}du\right),\;R_{t}=B_{t}^{-1}=\exp\left(-\int_{0}^{t}r_{u}du\right),\;t\in T.$$

Since $\int_{0}^{t}r_{u}du$ is continuous, *R* is continuous. Its differential is $dR_{t}=-r_{t}R_{t}dt$. Because *r* is càdlàg, we have $r_{t}=r_{t-}$ and $R_{t}=R_{t-}$ for all t∈T outside a Lebesgue-null set. Hence, we may write the drift in the predictable form. Thus, we have $dR_{t}=G_{R}\left(t\right)dt$, where $G_{R}\left(t\right)=-r_{t-}R_{t-}$, and this predictable version of the integrand will be used in the subsequent calculations. *R* has no Brownian part and no jump part; it is a finite-variation semimartingale. By Theorem 2, $S\in \Lambda^{p}$. We have $|F_{S}{\left(t\right)|}^{2}\leq 1+{\left|F_{S}\left(t\right)\right|}^{p}\leq 1+C_{\sigma }\left(1+|S_{t-}{|}^{p}\right)\leq C_{\sigma }^{\prime \prime }\left(1+|S_{t-}{|}^{p}\right)$, t∈T, for $C_{\sigma }^{\prime \prime }=C_{\sigma }+1$. Therefore,

(A4) $$\begin{array}{c} \begin{array}{cc} E\int_{0}^{T}{\left|F_{S}\left(t\right)\right|}^{2}dt & \leq C_{\sigma }^{\prime \prime }E\left(T+\int_{0}^{T}{\left|S_{t-}\right|}^{p}dt\right)=C_{\sigma }^{\prime \prime }E\left(T+\int_{0}^{T}{\left|S_{t}\right|}^{p}dt\right)\\ \; & \leq C_{\sigma }^{\prime \prime }TE\left(1+\underset{u\leq T}{\sup}{\left|S_{u}\right|}^{p}\right)=C_{\sigma }^{\prime \prime }T\left(1+E\left[\underset{u\leq T}{\sup}{\left|S_{u}\right|}^{p}\right]\right)<\infty . \end{array} \end{array}$$

Since $S_{t-}$ is predictable and σ is Borel measurable, $F_{S}$ is predictable. Using the fact that $S\in \Lambda^{p}$ and Proposition 2 with q=p, we obtain

$$\begin{array}{cc} E & \left[\int_{0}^{T}\left|S_{t-}r_{t-}\right|dt\right]\leq \frac{1}{2}E\int_{0}^{T}\left(|S_{t-}{|}^{2}+{\left|r_{t-}\right|}^{2}\right)dt\\ \; & \leq \frac{1}{2}E\int_{0}^{T}\left(1+|S_{t-}{|}^{p}+1+{\left|r_{t-}\right|}^{p}\right)dt=\frac{1}{2}E\int_{0}^{T}\left(2+|S_{t}{|}^{p}+{\left|r_{t}\right|}^{p}\right)dt\\ \; & \leq \frac{T}{2}E\left(2+\underset{u\leq T}{\sup}|S_{u}{|}^{p}+\underset{u\leq T}{\sup}{\left|r_{u}\right|}^{p}\right)\leq \frac{T}{2}\left(2+E\left[\underset{u\leq T}{\sup}{\left|S_{u}\right|}^{p}\right]+E\left[\underset{u\leq T}{\sup}{\left|r_{u}\right|}^{p}\right]\right)<\infty . \end{array}$$

By Assumption 2, $C_{k}=\int_{|y|\geq 1}K^{\left(k\right)}\left(y\right)\bar{\nu }\left(dy\right)<\infty$ and we have

$$\left|\int_{|y|\geq 1}{\tilde{k}}_{2}\left(t,S_{t-},y\right)\bar{\nu }\left(dy\right)\right|\leq \left(1+\right|S_{t-}\left|\right)\int_{|y|\geq 1}K^{\left(k\right)}\left(y\right)\bar{\nu }\left(dy\right)=C_{k}\left(1+\right|S_{t-}\left|\right).$$

Thus,

$$\begin{array}{cc} E & \left[\int_{0}^{T}\left|\int_{|y|\geq 1}{\tilde{k}}_{2}\left(t,S_{t-},y\right)\bar{\nu }\left(dy\right)\right|dt\right]\leq C_{k}E\left[\int_{0}^{T}\left(1+\right|S_{t-}\left|\right)dt\right]\leq C_{k}E\left[\int_{0}^{T}\left(1+\right|S_{t}\left|\right)dt\right]\\ \; & \leq C_{k}E\left[\int_{0}^{T}\left(1+1+\right|S_{t}{|}^{p})dt\right]\leq C_{k}T(2+E\underset{u\leq T}{\sup}|S_{u}{|}^{p})<\infty . \end{array}$$

Since $|G_{S}\left(t\right)|\leq |S_{t-}r_{t-}|+\left|\int_{|y|\geq 1}{\tilde{k}}_{2}\left(t,S_{t-},y\right)\bar{\nu }\left(dy\right)\right|$, we have

$$\begin{array}{cc} E\left[\int_{0}^{T}\left|G_{S}\left(t\right)\right|dt\right] & \leq E\left[\int_{0}^{T}\left|S_{t-}r_{t-}\right|dt\right]+E\left[\int_{0}^{T}\left|\int_{|y|\geq 1}{\tilde{k}}_{2}\left(t,S_{t-},y\right)\bar{\nu }\left(dy\right)\right|dt\right]<\infty . \end{array}$$

Since $S_{t-}$, $r_{t-}$, and ${\tilde{k}}_{2}$ are predictable, $G_{S}$ is predictable. By Assumption 2, we have $D_{h}=2\int_{\left\{|y|<1\right\}}{\left(K^{\left(h\right)}\left(y\right)\right)}^{2}\bar{\nu }\left(dy\right)<\infty$ and $D_{k}=2\int_{|y|\geq 1}{\left(K^{\left(k\right)}\left(y\right)\right)}^{2}\bar{\nu }\left(dy\right)<\infty$. Therefore, for t∈T,

$$\begin{array}{cc} \int_{|y|<1} & {|H_{S}\left(t,y\right)|}^{2}\bar{\nu }\left(dy\right)=\int_{|y|<1}{\left|{\tilde{h}}_{2}\left(t,S_{t-},y\right)\right|}^{2}\bar{\nu }\left(dy\right)\\ \; & \leq \int_{|y|<1}\left(1+\right|S_{t-}{\left|\right)}^{2}{\left(K^{\left(h\right)}\left(y\right)\right)}^{2}\bar{\nu }\left(dy\right)\leq D_{h}\left(1+|S_{t-}{|}^{2}\right)\\ \; & \leq D_{h}\left(1+1+|S_{t-}{|}^{p}\right)=D_{h}\left(2+|S_{t-}{|}^{p}\right) \end{array}$$

and

$$\begin{array}{cc} \int_{|y|\geq 1} & {|K_{S}\left(t,y\right)|}^{2}\bar{\nu }\left(dy\right)=\int_{|y|\geq 1}{\left|{\tilde{k}}_{2}\left(t,S_{t-},y\right)\right|}^{2}\bar{\nu }\left(dy\right)\\ \; & \leq \int_{|y|\geq 1}\left(1+\right|S_{t-}{\left|\right)}^{2}{\left(K^{\left(k\right)}\left(y\right)\right)}^{2}\bar{\nu }\left(dy\right)\leq D_{k}\left(1+|S_{t-}{|}^{2}\right)\\ \; & \leq D_{k}\left(1+1+|S_{t-}{|}^{p}\right)=D_{k}\left(2+|S_{t-}{|}^{p}\right). \end{array}$$

Thus,

(A5) $$\begin{array}{c} \begin{array}{cc} E & \left[\int_{0}^{T}\int_{|y|<1}{\left|H_{S}\left(t,y\right)\right|}^{2}\bar{\nu }\left(dy\right)dt\right]\leq D_{h}E\left[\int_{0}^{T}\left(2+|S_{t-}{|}^{p}\right)dt\right]\\ \; & =D_{h}E\left[\int_{0}^{T}\left(2+|S_{t}{|}^{p}\right)dt\right]\leq D_{h}T\left(2+E\left[\underset{u\leq T}{\sup}{\left|S_{u}\right|}^{p}\right]\right)<\infty , \end{array} \end{array}$$

(A6) $$\begin{array}{c} \begin{array}{cc} E & \left[\int_{0}^{T}\int_{|y|\geq 1}{\left|K_{S}\left(t,y\right)\right|}^{2}\bar{\nu }\left(dy\right)dt\right]\leq D_{k}E\left[\int_{0}^{T}\left(2+|S_{t-}{|}^{p}\right)dt\right]\\ \; & =D_{k}E\left[\int_{0}^{T}\left(2+|S_{t}{|}^{p}\right)dt\right]\leq D_{k}T\left(2+E\left[\underset{u\leq T}{\sup}{\left|S_{u}\right|}^{p}\right]\right)<\infty . \end{array} \end{array}$$

Finally, by Proposition 2,

(A7) $$\begin{array}{c} \begin{array}{cc} E & \left[\int_{0}^{T}{\left|G_{R}\left(t\right)\right|}^{2}dt\right]=E\left[\int_{0}^{T}{\left|r_{t-}R_{t}\right|}^{2}dt\right]=E\left[\int_{0}^{T}{\left|r_{t}R_{t}\right|}^{2}dt\right]\\ \; & \leq \frac{1}{2}\left(\int_{0}^{T}E{\left|r_{t}\right|}^{4}dt+\int_{0}^{T}E{\left|R_{t}\right|}^{4}dt\right)<\infty . \end{array} \end{array}$$

From the predictability of the integrands $G_{S},F_{S},H_{S},K_{S}$ and $G_{R}$, together with the estimates (A4)–(A7), it follows that *S* and *R* are of the form of a Lévy-type stochastic integral [29] (Eq. (4.13)).

$$dS_{t}=G_{S}\left(t\right)dt+F_{S}\left(t\right)dW_{t}^{\left(2\right)}+\int_{|y|<1}H_{S}\left(t,y\right)q\left(dt\;dy\right)+\int_{|y|\geq 1}K_{S}\left(t,y\right)N\left(dt\;dy\right),$$

$$dR_{t}=G_{R}\left(t\right)dt=-r_{t-}R_{t-}dt.$$

Since *R* has finite variation, ${\left[S,R\right]}_{t}\equiv 0$. By Itô’s product formula (see, [29] (Thm. 4.4.13)),

$$\begin{array}{cc} S_{t}R_{t} & =S_{0}R_{0}+\int_{0}^{t}S_{s-}dR_{s}+\int_{0}^{t}R_{s-}dS_{s}=S_{0}+\int_{0}^{t}S_{s-}G_{R}\left(s\right)ds\\ \; & +\int_{0}^{t}R_{s-}G_{S}\left(s\right)ds+\int_{0}^{t}R_{s-}F_{S}\left(s\right)dW_{s}^{\left(2\right)}+\int_{0}^{t}\int_{|y|<1}R_{s-}H_{S}\left(s,y\right)q\left(ds\;dy\right)\\ \; & +\int_{0}^{t}\int_{|y|\geq 1}R_{s-}K_{S}\left(s,y\right)N\left(ds\;dy\right). \end{array}$$

Thus, the SDE of the product is

$$d\left(S_{t}R_{t}\right)=G_{SR}\left(t\right)dt+F_{SR}\left(t\right)dW_{t}^{\left(2\right)}+\int_{|y|<1}H_{SR}\left(t,y\right)q\left(dt\;dy\right)+\int_{|y|\geq 1}K_{SR}\left(t,y\right)N\left(dt\;dy\right),$$

where

$$\begin{array}{cc} G_{SR}\left(t\right) & =S_{t-}G_{R}\left(t\right)+R_{t-}G_{S}\left(t\right)=S_{t-}\left(-r_{t-}R_{t-}\right)\\ \; & +R_{t-}\left(S_{t-}r_{t-}-\int_{|y|\geq 1}{\tilde{k}}_{2}\left(t,S_{t-},y\right)\bar{\nu }\left(dy\right)\right)=-R_{t-}\int_{|y|\geq 1}{\tilde{k}}_{2}\left(t,S_{t-},y\right)\bar{\nu }\left(dy\right). \end{array}$$

$$F_{SR}\left(t\right)=R_{t-}F_{S}\left(t\right),\;H_{SR}\left(t,y\right)=R_{t-}H_{S}\left(t,y\right),\;K_{SR}\left(t,y\right)=R_{t-}K_{S}\left(t,y\right).$$

In our formulation, the term $G_{SR}\left(t\right)$ is incorporated into the compensator of the large-jump component, and therefore the drift of $S_{t}R_{t}$ is zero. Hence,

$$S_{t}R_{t}=S_{0}+\int_{0}^{t}F_{SR}\left(s\right)dW_{s}^{\left(2\right)}+\int_{0}^{t}\int_{R}H_{SR}\left(s,y\right)q\left(ds\;dy\right)+\int_{0}^{t}\int_{R}K_{SR}\left(s,y\right)q\left(ds\;dy\right).$$

Choosing $C_{\sigma }={\left(C_{\sigma }^{\prime }\right)}^{1/2}$, as in Assumption 2, we have for all x∈R, ${\left|x\sigma \left(t,x\right)\right|}^{2}={\left({\left|x\sigma \left(t,x\right)\right|}^{p}\right)}^{2/p}\leq {\left(C_{\sigma }\right)}^{2/p}{\left(1+{\left|x\right|}^{p}\right)}^{2/p}$. Therefore,

$$\begin{array}{cc} E & \int_{0}^{T}|F_{SR}{\left(t\right)|}^{2}dt=E\int_{0}^{T}|R_{t-}F_{S}{\left(t\right)|}^{2}dt=E\int_{0}^{T}{\left|R_{t}F_{S}\left(t\right)\right|}^{2}dt\leq E\left[\underset{u\leq T}{\sup}|R_{u}{|}^{2}\int_{0}^{T}{\left|F_{S}\left(t\right)\right|}^{2}dt\right]\\ \; & \leq E\left[\underset{u\leq T}{\sup}|R_{u}{|}^{2}\int_{0}^{T}C_{\sigma }^{2/p}\left(1+\right|S_{t-}{{|}^{p})}^{2/p}dt\right]=E\left[\underset{u\leq T}{\sup}|R_{u}{|}^{2}\int_{0}^{T}C_{\sigma }^{2/p}\left(1+\right|S_{t}{{|}^{p})}^{2/p}dt\right]\\ \; & \leq TC_{\sigma }^{2/p}E\left[\underset{u\leq T}{\sup}|R_{u}{|}^{2}(1+\underset{u\leq T}{\sup}|S_{u}{{|}^{p})}^{2/p}\right]\\ \; & \leq TC_{\sigma }^{2/p}{\left(E\underset{u\leq T}{\sup}{\left|R_{u}\right|}^{4}\right)}^{1/2}{\left(E{\left(1+\underset{u\leq T}{\sup}{\left|S_{u}\right|}^{p}\right)}^{4/p}\right)}^{1/2}\\ \; & \leq TC_{\sigma }^{2/p}{\left(E\underset{u\leq T}{\sup}{\left|R_{u}\right|}^{4}\right)}^{1/2}{\left(1+E\underset{u\leq T}{\sup}{\left|S_{u}\right|}^{p}\right)}^{1/2}<\infty , \end{array}$$

which implies that $\int_{0}^{t}F_{SR}\left(s\right)dW_{s}^{\left(2\right)}$, t∈T, is a square-integrable martingale. By Assumption 2, we have $D_{h}^{\prime }=\int_{R}{\left(K^{\left(h\right)}\left(y\right)\right)}^{2}\bar{\nu }\left(dy\right)<\infty$. Therefore,

$$\begin{array}{cc} E & \int_{0}^{T}\int_{R}|H_{SR}{\left(t,y\right)|}^{2}\bar{\nu }\left(dy\right)dt=E\int_{0}^{T}\int_{R}{\left|R_{t-}H_{S}\left(t,y\right)\right|}^{2}\bar{\nu }\left(dy\right)dt\\ \; & \leq E\left[\underset{u\leq T}{\sup}|R_{u}{|}^{2}\int_{0}^{T}\int_{R}{\left|H_{S}\left(t,y\right)\right|}^{2}\bar{\nu }\left(dy\right)dt\right]\\ \; & =E\left[\underset{u\leq T}{\sup}|R_{u}{|}^{2}\int_{0}^{T}\int_{R}{\left|{\tilde{h}}_{2}\left(t,S_{t-},y\right)\right|}^{2}\bar{\nu }\left(dy\right)dt\right]\\ \; & \leq E\left[\underset{u\leq T}{\sup}|R_{u}{|}^{2}\int_{0}^{T}\int_{R}\left(1+\right|S_{t-}{\left|\right)}^{2}{\left(K^{\left(h\right)}\left(y\right)\right)}^{2}\bar{\nu }\left(dy\right)dt\right]\\ \; & \leq 2E\left[\underset{u\leq T}{\sup}{\left|R_{u}\right|}^{2}\int_{0}^{T}\left(1+|S_{t-}{|}^{2}\right)\int_{R}{\left(K^{\left(h\right)}\left(y\right)\right)}^{2}\bar{\nu }\left(dy\right)dt\right]\\ \; & =2D_{h}^{\prime }E\left[\underset{u\leq T}{\sup}{\left|R_{u}\right|}^{2}\int_{0}^{T}\left(1+|S_{t-}{|}^{2}\right)dt\right]\leq 2D_{h}^{\prime }E\left[\underset{u\leq T}{\sup}{\left|R_{u}\right|}^{2}\int_{0}^{T}\left(1+1+|S_{t-}{|}^{p/2}\right)dt\right]\\ \; & =2D_{h}^{\prime }E\left[\underset{u\leq T}{\sup}{\left|R_{u}\right|}^{2}\int_{0}^{T}\left(2+|S_{t-}{|}^{p/2}\right)dt\right]=2D_{h}^{\prime }E\left[\underset{u\leq T}{\sup}{\left|R_{u}\right|}^{2}\int_{0}^{T}\left(2+|S_{t}{|}^{p/2}\right)dt\right]\\ \; & \leq 2D_{h}^{\prime }TE\left[\underset{u\leq T}{\sup}{\left|R_{u}\right|}^{2}\left(2+\underset{u\leq T}{\sup}{\left|S_{u}\right|}^{p/2}\right)\right]\\ \; & \leq 2D_{h}^{\prime }TE{\left(\underset{u\leq T}{\sup}{\left|R_{u}\right|}^{4}\right)}^{1/2}E{\left({\left(2+\underset{u\leq T}{\sup}{\left|S_{u}\right|}^{p/2}\right)}^{2}\right)}^{1/2}\\ \; & \leq 2\sqrt{2}D_{h}^{\prime }TE{\left(\underset{u\leq T}{\sup}{\left|R_{u}\right|}^{4}\right)}^{1/2}{\left(4+E\underset{u\leq T}{\sup}{\left|S_{u}\right|}^{p}\right)}^{1/2}<\infty . \end{array}$$

A similar argument gives $E\int_{0}^{T}\int_{R}{\left|K_{SR}\left(t,y\right)\right|}^{2}\bar{\nu }\left(dy\right)dt<\infty$. Thus, $H_{SR},$$K_{SR}\in M_{loc}^{2}\left(\hat{P},\bar{\nu },R\right)$, and therefore, $\int_{0}^{t}\int_{R}H_{SR}\left(s,y\right)q\left(ds\;dy\right)$ and $\int_{0}^{t}\int_{R}K_{SR}\left(s,y\right)q\left(ds\;dy\right)$, t∈T, are square-integrable martingales. Finally, since all three stochastic integrals in the decomposition of SR=S/B are square-integrable martingales, S/B is a square-integrable martingale. □

**Proof** **of Lemma 10.** By Theorem 2, the SDE (13) admits a unique càdlàg solution $S\in \Lambda^{4}\left(T\right)$. Moreover, Lemma 1 implies that for every q≥1, ${\left(R_{t}\right)}_{t\in T}\in \Lambda^{q}$. By the Hölder inequality, Proposition 2, and Lemma 1,

$$E\int_{0}^{T}r_{t-}^{2}R_{t}^{2}dt\leq E\int_{0}^{T}\underset{u\leq T}{\sup}{\left|r_{u}\right|}^{2}\cdot \underset{u\leq T}{\sup}R_{u}^{2}dt\leq T{\left(E\underset{u\leq T}{\sup}{\left|r_{u}\right|}^{4}\right)}^{1/2}{\left(E\underset{u\leq T}{\sup}R_{u}^{4}\right)}^{1/2}<\infty .$$

From ${\left|x\sigma \left(t,x\right)\right|}^{8}\leq C_{\sigma }^{\prime }{\left(1+|x\right|}^{8})$, we obtain $S_{t-}^{2}\sigma^{2}\left(t,S_{t-}\right)\leq {\left(C^{\prime }\right)}_{\sigma }^{1/4}\left(1+\right|S_{t-}{|}^{2})$, and thus,

$$E\int_{0}^{T}S_{t-}^{2}\sigma^{2}\left(t,S_{t-}\right)dt\leq {\left(C^{\prime }\right)}_{\sigma }^{1/4}T\left(1+E\underset{t\leq T}{\sup}{\left|S_{t}\right|}^{2}\right)<\infty .$$

In turn, ${\left(S_{t-}r_{t-}-\int_{R}{\tilde{k}}_{2}\left(t,S_{t-},y\right)\bar{\nu }\left(dy\right)\right)}^{2}\leq 2S_{t-}^{2}r_{t-}^{2}+2{\left(\int_{R}{\tilde{k}}_{2}\left(t,S_{t-},y\right)\bar{\nu }\left(dy\right)\right)}^{2}$. By the Hölder inequality,

$$E\int_{0}^{T}S_{t-}^{2}r_{t-}^{2}dt\leq TE\left[\underset{u\leq T}{\sup}{\left|S_{u}\right|}^{2}\underset{u\leq T}{\sup}{\left|r_{u}\right|}^{2}\right]\leq T{\left({E\sup|S|}^{4}\right)}^{1/2}{\left({E\sup|r|}^{4}\right)}^{1/2}<\infty .$$

Since $K^{\left(h\right)},K^{\left(k\right)}\in L^{8}\left(\bar{\nu }\right)$ and $\bar{\nu }$ is a finite measure, we also have $K^{\left(h\right)},K^{\left(k\right)}\in L^{q}\left(\bar{\nu }\right)$ for all 1≤q<8. Thus, $|{\tilde{k}}_{2}\left(t,S_{t-},y\right)|\leq K^{\left(k\right)}\left(y\right)\left(1+\right|S_{t-}\left|\right)$ implies

$$\begin{array}{cc} E\int_{0}^{T} & {\left(\int_{R}{\tilde{k}}_{2}\left(t,S_{t-},y\right)\bar{\nu }\left(dy\right)\right)}^{2}dt\leq E\int_{0}^{T}{\left(\int_{R}K^{\left(k\right)}\left(y\right)\bar{\nu }\left(dy\right)\right)}^{2}{\left(1+|S_{t-}|\right)}^{2}dt\\ \; & \leq 2{\left(\int_{R}K^{\left(k\right)}\left(y\right)\bar{\nu }\left(dy\right)\right)}^{2}E\int_{0}^{T}\left(1+|S_{t-}{|}^{2}\right)dt\\ \; & \leq 2{\left(\int_{R}K^{\left(k\right)}\left(y\right)\bar{\nu }\left(dy\right)\right)}^{2}T\left(1+E\underset{t\leq T}{\sup}{\left|S_{t}\right|}^{2}\right)<\infty . \end{array}$$

We also have

$$\begin{array}{cc} E & \int_{0}^{T}\int_{R}\left({\tilde{h}}_{2}^{4}\left(t,S_{t-},y\right)+{\tilde{k}}_{2}^{4}\left(t,S_{t-},y\right)\right)\bar{\nu }\left(dy\right)dt\\ \; & \leq \left(\int_{R}{\left(K^{\left(h\right)}\right)}^{4}\left(y\right)\bar{\nu }\left(dy\right)+\int_{R}{\left(K^{\left(k\right)}\right)}^{4}\left(y\right)\bar{\nu }\left(dy\right)\right)E\int_{0}^{T}\left(1+\right|S_{t-}{\left|\right)}^{4}dt\\ \; & \leq 8\left(\int_{R}{\left(K^{\left(h\right)}\right)}^{4}\left(y\right)\bar{\nu }\left(dy\right)+\int_{R}{\left(K^{\left(k\right)}\right)}^{4}\left(y\right)\bar{\nu }\left(dy\right)\right)E\int_{0}^{T}\left(1+|S_{t-}{|}^{4}\right)dt\\ \; & \leq 8\left(\int_{R}{\left(K^{\left(h\right)}\right)}^{4}\left(y\right)\bar{\nu }\left(dy\right)+\int_{R}{\left(K^{\left(k\right)}\right)}^{4}\left(y\right)\bar{\nu }\left(dy\right)\right)T\left(1+E\underset{t\leq T}{\sup}{\left|S_{t}\right|}^{4}\right)<\infty . \end{array}$$

Furthermore, $S_{t-}^{2}\leq \sup_{u\leq T}{\left|S_{u}\right|}^{2}$ implies $E\int_{0}^{T}S_{t-}^{2}dt\leq TE\sup_{t\leq T}{\left|S_{t}\right|}^{2}<\infty$. Using $R_{t}^{2}\leq \sup_{u\leq T}R_{u}^{2}$ and the Hölder inequality, we obtain

$$\begin{array}{cc} E\int_{0}^{T}R_{t}^{2} & \left(1+S_{t-}^{2}+S_{t-}^{2}\sigma^{2}\left(t,S_{t-}\right)+\int_{R}\left({\tilde{h}}_{2}^{2}\left(t,S_{t-},y\right)+{\tilde{k}}_{2}^{2}\left(t,S_{t-},y\right)\right)\bar{\nu }\left(dy\right)\right)dt\\ \; & \leq {\left(E\underset{t\leq T}{\sup}R_{t}^{4}\right)}^{1/2}\left(E\left[\left(\int_{0}^{T}\left(1+S_{t-}^{2}+S_{t-}^{2}\sigma^{2}\left(t,S_{t-}\right)\right.\right.\right.\right.\\ \; & {\left.\left.{\left.\left.+\int_{R}\left({\tilde{h}}_{2}^{2}\left(t,S_{t-},y\right)+{\tilde{k}}_{2}^{2}\left(t,S_{t-},y\right)\right)\bar{\nu }\left(dy\right)\right)dt\right)}^{2}\right]\right)}^{1/2}, \end{array}$$

which is finite since

$$\begin{array}{cc} E & {\left(\int_{0}^{T}\left(1+S_{t-}^{2}+S_{t-}^{2}\sigma^{2}\left(t,S_{t-}\right)+\int_{R}\left({\tilde{h}}_{2}^{2}\left(t,S_{t-},y\right)+{\tilde{k}}_{2}^{2}\left(t,S_{t-},y\right)\right)\bar{\nu }\left(dy\right)\right)dt\right)}^{2}\\ \; & \leq E{\left(\int_{0}^{T}\left(1+{\left(C^{\prime }\right)}_{\sigma }^{1/4}+2\left(\int_{R}{\left(K^{\left(h\right)}\right)}^{2}\left(y\right)\bar{\nu }\left(dy\right)+\int_{R}{\left(K^{\left(k\right)}\right)}^{2}\left(y\right)\bar{\nu }\left(dy\right)\right)\right)\left(1+|S_{t-}{|}^{2}\right)dt\right)}^{2}\\ \; & \leq E{\left(\left(1+{\left(C^{\prime }\right)}_{\sigma }^{1/4}+2\left(\int_{R}{\left(K^{\left(h\right)}\right)}^{2}\left(y\right)\bar{\nu }\left(dy\right)+\int_{R}{\left(K^{\left(k\right)}\right)}^{2}\left(y\right)\bar{\nu }\left(dy\right)\right)\right)T\left(1+\underset{t\leq T}{\sup}{\left|S_{t}\right|}^{2}\right)\right)}^{2}\\ \; & \leq 2{\left(1+{\left(C^{\prime }\right)}_{\sigma }^{1/4}+2\left(\int_{R}{\left(K^{\left(h\right)}\right)}^{2}\left(y\right)\bar{\nu }\left(dy\right)+\int_{R}{\left(K^{\left(k\right)}\right)}^{2}\left(y\right)\bar{\nu }\left(dy\right)\right)\right)}^{2}\\ \; & \cdot T^{2}\left(1+E\underset{t\leq T}{\sup}{\left|S_{t}\right|}^{4}\right)<\infty . \end{array}$$

By the growth inequalities established above, $E\int_{0}^{T}H\left(t\right)dt<\infty$. □

**Proof** **of Lemma 11.** The solution of (92) has the form (see, e.g., [23])

$$r_{t}=b+\left(r_{0}-b\right)e^{-at}+\sigma_{r}\int_{0}^{t}e^{-a(t-s)}dW_{s}.$$

Since $b+\left(r_{0}-b\right)e^{-at}$ is a continuous and deterministic function, there exists $C_{0,r}>0$ such that

(A8) $$\underset{0\leq t\leq T}{\sup}|r_{t}|\leq C_{0,r}+\underset{0\leq t\leq T}{\sup}\left|Z_{t}\right|,$$

where $Z_{t}=\sigma_{r}\int_{0}^{t}e^{-a(t-s)}dW_{s}$. Let $M_{t}=\int_{0}^{t}e^{as}dW_{s},\;t\in T$. Then *M* is a continuous martingale, $Z_{t}=\sigma_{r}e^{-at}M_{t},\;t\in T$, and ${\langle M\rangle }_{t}=\int_{0}^{t}e^{2as}ds=\frac{e^{2at}-1}{2a},\;t\in T$. Since $e^{-at}\leq 1$ for all t∈T, we have $\sup_{0\leq t\leq T}|Z_{t}|\leq \sigma_{r}\sup_{0\leq t\leq T}\left|M_{t}\right|$. For λ∈R, the process $M_{t}\left(\lambda \right)=\exp\left(\lambda M_{t}-\frac{1}{2}\lambda^{2}{\langle M\rangle }_{t}\right),\;t\in T$, is a positive local martingale with $M_{0}\left(\lambda \right)=1$ (see, e.g., [31] (Lemma 16.21)) and since ${\langle M\rangle }_{T}=\frac{e^{2aT}-1}{2a}$, M(λ) is a martingale with $EM_{t}\left(\lambda \right)=EM_{0}\left(\lambda \right)=1,\;t\in T$ (see, e.g., [31] (Theorem 16.23)). Let λ>0 and x>0. Then $\left\{\sup_{0\leq t\leq T}M_{t}>x\right\}\subset \left\{\sup_{0\leq t\leq T}M_{t}\left(\lambda \right)>e^{\lambda x-\frac{1}{2}\lambda^{2}{\langle M\rangle }_{T}}\right\}$. By Doob’s maximal inequality (see, e.g., [38] (Thm. (1.7))),

(A9) $$\begin{array}{c} \begin{array}{cc} Q\left(\underset{0\leq t\leq T}{\sup}M_{t}\left(\lambda \right)>e^{\lambda x-\frac{1}{2}\lambda^{2}{\langle M\rangle }_{T}}\right) & \leq Q\left(\underset{0\leq t\leq T}{\sup}M_{t}\left(\lambda \right)\geq e^{\lambda x-\frac{1}{2}\lambda^{2}{\langle M\rangle }_{T}}\right)\\ \; & \leq \frac{EM_{T}\left(\lambda \right)}{e^{\lambda x-\frac{1}{2}\lambda^{2}{\langle M\rangle }_{T}}}=\exp\left(-\lambda x+\frac{1}{2}\lambda^{2}{\langle M\rangle }_{T}\right),\;x,\lambda >0. \end{array} \end{array}$$

Since the right-hand side of (A9) is minimised at $\lambda^{*}=x/{\langle M\rangle }_{T}$, we obtain $Q\left(\sup_{0\leq t\leq T}M_{t}>x\right)\leq \exp\left(-\frac{x^{2}}{2{\langle M\rangle }_{T}}\right),\;x>0$. Applying the same reasoning for $-M_{t}$, we get $Q(\sup_{0\leq t\leq T}|M_{t}\left|>x\right)\leq 2\exp\left(-\frac{x^{2}}{2{\langle M\rangle }_{T}}\right),\;x>0$, and since

$$\underset{0\leq t\leq T}{\sup}|Z_{t}|\leq \sigma_{r}\underset{0\leq t\leq T}{\sup}\left|M_{t}\right|,$$

it follows that

$$Q\left(\underset{0\leq t\leq T}{\sup}\left|Z_{t}\right|>x\right)\leq 2\exp\left(-\frac{x^{2}}{2\sigma_{r}^{2}{\langle M\rangle }_{T}}\right),\;x>0.$$

By (A8), for every $x>C_{0,r}$,

(A10) $$\begin{array}{c} Q\left(\underset{0\leq t\leq T}{\sup}\left|r_{t}\right|>x\right)\leq Q\left(\underset{0\leq t\leq T}{\sup}\left|Z_{t}\right|>x-C_{0,r}\right)\leq 2\exp\left(-\frac{{\left(x-C_{0,r}\right)}^{2}}{2\sigma_{r}^{2}{\langle M\rangle }_{T}}\right),\;x>C_{0,r}. \end{array}$$

We extend this estimate to all x≥0. Let $c_{r}=\frac{1}{8\sigma_{r}^{2}{\langle M\rangle }_{T}}$. For $x\in [0,2C_{0,r}]$,

$$Q\left(\underset{0\leq t\leq T}{\sup}\left|r_{t}\right|>x\right)\leq 1\leq \frac{1}{m}e^{-c_{r}x^{2}}=e^{c_{r}{\left(2C_{0,r}\right)}^{2}}e^{-c_{r}x^{2}},$$

where $m=\min_{0\leq x\leq 2C_{0,r}}e^{-c_{r}x^{2}}=e^{-c_{r}{\left(2C_{0,r}\right)}^{2}}$. If $x\geq 2C_{0,r}$, then $x-C_{0,r}\geq \frac{x}{2}$ and by (A10),

$$Q\left(\underset{0\leq t\leq T}{\sup}\left|r_{t}\right|>x\right)\leq Q\left(\underset{0\leq t\leq T}{\sup}\left|Z_{t}\right|>x-C_{0,r}\right)\leq 2e^{-\frac{{\left(x-C_{0,r}\right)}^{2}}{2\sigma_{r}^{2}{\langle M\rangle }_{T}}}\leq 2e^{-\frac{{\left(x/2\right)}^{2}}{2\sigma_{r}^{2}{\langle M\rangle }_{T}}}=2e^{-c_{r}x^{2}}.$$

Thus, $Q(\sup_{0\leq t\leq T}|r_{t}\left|>x\right)\leq C_{r}e^{-c_{r}x^{2}},\;x\geq 0$, where $C_{r}=\max\left\{2,e^{c_{r}{\left(2C_{0,r}\right)}^{2}}\right\}$. □

## References

[1] Black F., Scholes M. (1973). The pricing of options and corporate liabilities. J. Polit. Econ..

[2] Brigo D., Mercurio F. (2007). Interest Rate Models-Theory and Practice: With Smile, Inflation and Credit.

[3] Dupire B. (1994). Pricing with a smile. Risk.

[4] Derman E., Kani I. (1994). Riding on a smile. Risk.

[5] Amster P., De Nápoli P., Zubelli J.P. (2009). Towards a generalization of Dupire’s equation for several assets. J. Math. Anal. Appl..

[6] Hamza K., Klebaner F.C. (2022). Expectation of local times and the Dupire formula. Stoch. Proc. Appl..

[7] Itkin A. (2020). Fitting Local Volatility: Analytic and Numerical Approaches in Black-Scholes and Local Variance Gamma Models.

[8] Andersen L., Andreasen J. (2000). Jump-Diffusion Processes: Volatility Smile Fitting and Numerical Methods for Option Pricing. Rev. Deriv. Res..

[9] Carr P., Geman H., Madan D.B., Yor M. (2004). From local volatility to local Lévy models. Quant. Finance.

[10] Bentata A., Cont R. (2015). Forward equations for option prices in semimartingale models. Finance Stoch..

[11] Deelstra G., Rayée G. (2012). Local Volatility Pricing Models for Long-Dated FX Derivatives. Appl. Math. Finance.

[12] Grzelak L.A., Borovykh N., van Weeren S., Oosterlee C.W. (2008). Incorporating an Interest Rate Smile in an Equity Local Volatility Model. https://ssrn.com/abstract=1295882.

[13] Ögetbil O., Ganesan N., Hientzsch B. (2022). Calibrating Local Volatility Models With Stochastic Drift And Diffusion. Int. J. Theor. Appl. Finance.

[14] Benhamou E., Rivoira A., Gruz A. (2008). Stochastic Interest Rates for Local Volatility Hybrids Models. https://ssrn.com/abstract=1107711.

[15] Margrabe W. (1978). The value of an option to exchange one asset for another. J. Finance.

[16] Alòs E., Coulon M. (2024). On the Optimal Choice of Strike Conventions in Exchange Option Pricing. Mathematics.

[17] Gatarek D., Jabłecki J. (2019). Towards a General Local Volatility Model for All Asset Classes. J. Deriv..

[18] Nowak P., Gatarek D. (2022). Application of Itô processes and Schwartz distributions to local volatility for Margrabe options. Stochastics.

[19] Nowak P., Gatarek D. (2026). Schwartz distributions approach to local volatility for asset exchange options in a Lévy-type stochastic integrals setting. Stochastics.

[20] Nowak P., Gatarek D. (2025). Distribution Approach to Local Volatility for European Options in the Merton Model with Stochastic Interest Rates. Entropy.

[21] Hambly B., Mariapragassam M., Reisinger C. (2016). A forward equation for barrier options under the Brunick & Shreve Markovian projection. Quant. Finance.

[22] Hainaut D., Leonenko N. (2021). Option pricing in illiquid markets: A fractional jump–diffusion approach. J. Comput. Appl. Math..

[23] Musiela M., Rutkowski M. (2005). Martingale Methods in Financial Modelling.

[24] Kulinich S., Kushnirenko A. (2014). Strong uniqueness for SDEs with jumps and random non-Lipschitz coefficients. Mod. Stoch. Theory Appl..

[25] Blanchard P., Brüning E. (2015). Mathematical Methods in Physics: Distributions, Hilbert Space Operators, Variational Methods, and Applications in Quantum Physics.

[26] Protter P.E. (2005). Stochastic Integration and Differential Equations.

[27] Øksendal B. (2008). Stochastic Partial Differential Equations Driven by Multi-parameter White Noise of Lévy Processes. Q. Appl. Math..

[28] Zhu J. (2010). A Study of SPDEs Wrt Compensated Poisson Random Measures and Related Topics. Ph.D. Thesis.

[29] Applebaum D. (2004). Levy Processes and Stochastic Calculus.

[30] Kunita H., Rao M.M. (2004). Stochastic Differential Equations Based on Lévy Processes and Stochastic Flows of Diffeomorphisms. Real and Stochastic Analysis. Trends in Mathematics.

[31] Kallenberg O. (1997). Foundations of Modern Probability.

[32] Billingsley P. (2012). Probability and Measure, Anniversary Edition.

[33] Schweizer M., Cont R. (2010). The minimal entropy martingale measure. Encyclopedia of Quantitative Finance.

[34] Miyahara Y. (2012). Option Pricing in Incomplete Markets: Modeling Based on Geometric Lévy Processes and Minimal Entropy Martingale Measures.

[35] Cover T., Thomas J. (1991). Elements of Information Theory.

[36] Frittelli M. (2000). The Minimal Entropy Martingale Measure and the Valuation Problem in Incomplete Markets. Math. Finance.

[37] Gut A. (2013). Probability: A Graduate Course.

[38] Revuz D., Yor M. (2005). Continuous Martingales and Brownian Motion.

